# Phosphorylation of iRhom2 Controls Stimulated Proteolytic Shedding by the Metalloprotease ADAM17/TACE

**DOI:** 10.1016/j.celrep.2017.09.074

**Published:** 2017-10-17

**Authors:** Miguel Cavadas, Ioanna Oikonomidi, Catarina J. Gaspar, Emma Burbridge, Marina Badenes, Inês Félix, Alfonso Bolado, Tianyi Hu, Andrea Bileck, Christopher Gerner, Pedro M. Domingos, Alex von Kriegsheim, Colin Adrain

**Affiliations:** 1 Membrane Traffic Lab, Instituto Gulbenkian de Ciência (IGC), Oeiras, Portugal; 2 Instituto de Tecnologia Química e Biológica (ITQB-NOVA), Oeiras, Portugal; 3 Edinburgh Cancer Research UK Centre, Institute of Genetics and Molecular Medicine, University of Edinburgh, Edinburgh, UK; 4 Institut für Analytische Chemie, Universität Wien, Währinger Strasse 38, 1090 Vienna, Austria

**Keywords:** ADAM metalloproteases, ADAM17/TACE, iRhom2, 14-3-3, MAP kinases, TNF, EGFR, ectodomain shedding

## Abstract

Cell surface metalloproteases coordinate signaling during development, tissue homeostasis, and disease. TACE (TNF-α-converting enzyme), is responsible for cleavage (“shedding”) of membrane-tethered signaling molecules, including the cytokine TNF, and activating ligands of the EGFR. The trafficking of TACE within the secretory pathway requires its binding to iRhom2, which mediates the exit of TACE from the endoplasmic reticulum. An important, but mechanistically unclear, feature of TACE biology is its ability to be stimulated rapidly on the cell surface by numerous inflammatory and growth-promoting agents. Here, we report a role for iRhom2 in TACE stimulation on the cell surface. TACE shedding stimuli trigger MAP kinase-dependent phosphorylation of iRhom2 N-terminal cytoplasmic tail. This recruits 14-3-3 proteins, enforcing the dissociation of TACE from complexes with iRhom2, promoting the cleavage of TACE substrates. Our data reveal that iRhom2 controls multiple aspects of TACE biology, including stimulated shedding on the cell surface.

## Highlights

• iRhom2 is phosphorylated in response to stimuli that activate the sheddase TACE
• Blocking iRhom phosphorylation represses TACE stimulated shedding
• Phosphorylated iRhom2 recruits 14-3-3 and dissociates from TACE, enabling shedding
• iRhom2 is thus a signal integrator and transducer of stimulated TACE shedding

## Graphical Abstract

**Figure:**
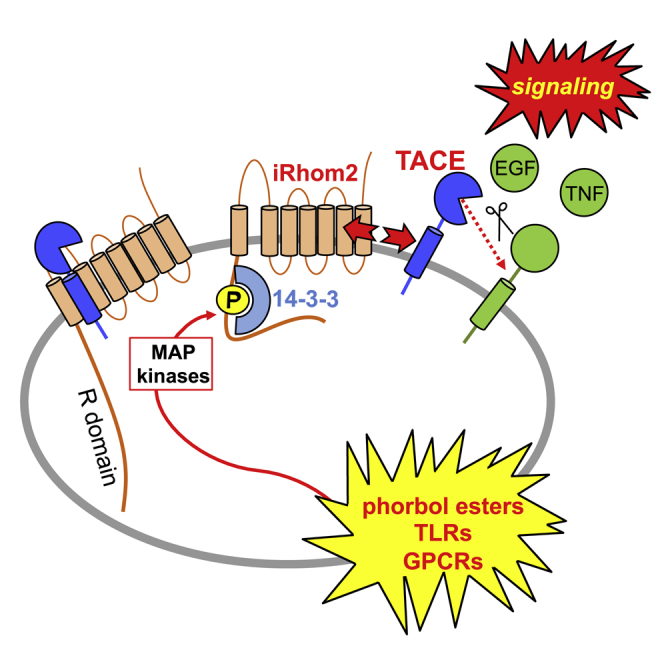

## Teaser

Cavadas et al. examine how the metalloprotease TACE is stimulated to shed its substrates, observing that iRhom2, a molecule essential for TACE trafficking, is phosphorylated in response to stimulants (PMA, TLRs, and GPCRs). iRhom phosphorylation requires MAPKs and recruits 14-3-3, which causes iRhom2/TACE dissociation, enabling TACE to cleave its substrates.

## Introduction

A major mechanism of cellular communication involves “shedding”: the stimulated proteolytic release of signaling molecules from the plasma membrane. The TNF-α-converting enzyme (TACE), also called ADAM17 (a disintegrin and metalloprotease-17), is a prominent sheddase with more than 80 cellular substrates (Gooz, 2010). Mutant mouse studies emphasize the essential role of TACE in inflammation: it sheds the inflammatory cytokine, tumor necrosis factor (TNF) (Horiuchi et al., 2007). TACE also plays an essential physiological role in growth factor signaling, by shedding multiple activating ligands of the epidermal growth factor receptor (EGFR), a receptor important for epithelial development, homeostasis, and cancer (Peschon et al., 1998).

TACE is synthesized as a catalytically inactive precursor in the endoplasmic reticulum (ER). To be proteolytically active, TACE must undergo a maturation step, which occurs in the *trans-*Golgi network, where pro-protein convertases cleave off TACE’s inhibitory N-terminal prodomain, rendering it basally active (Schlöndorff et al., 2000). The work of several groups, including ours, recently identified polytopic membrane proteins called iRhoms as essential regulators of TACE maturation (Adrain et al., 2012, Siggs et al., 2012, Christova et al., 2013). In iRhom null cells, TACE is retained in the ER, fails to undergo prodomain removal, and is consequently proteolytically inactive.

As overexpressed iRhoms are predominantly ER localized (Zettl et al., 2011), the current working hypothesis is that iRhoms mediate the ER-to-Golgi trafficking of TACE (Adrain and Freeman, 2012). However, several observations are incongruent with this model: first, cross-linking experiments show that iRhom binds efficiently to mature TACE, indicating that the molecules still interact following prodomain removal in the *trans-*Golgi network (Adrain et al., 2012). Second, endogenous iRhom2 contains endoglycosidase H-insensitive glycans, indicating that it traffics beyond the ER (Adrain et al., 2012). Moreover, overexpressed iRhoms localize to the plasma membrane (Maney et al., 2015).

The cell surface sheddase activity of TACE is subject to another important layer of regulation: stimulation by various signaling pathways. TACE stimulation is involved in inflammation, tissue damage, and cancer; stimulatory agents include the phorbol ester phorbol 12-myristate 13-acetate (PMA) (Arribas et al., 1996), cytokine receptors (Hall and Blobel, 2012),Toll-like receptors (Brandl et al., 2010), and G protein-coupled receptors (Prenzel et al., 1999, Wetzker and Böhmer, 2003). Many stimuli that activate TACE converge on the cytoplasm, activating kinases, including members of the MAP (mitogen-activated protein kinase) kinase family, that control release of several endogenous TACE substrates, including TNF and EGFR ligands (Díaz-Rodríguez et al., 2002, Rousseau et al., 2008, Xu and Derynck, 2010, Scott et al., 2011, Sommer et al., 2016). There is an emerging consensus that TACE-activating stimuli enforce rearrangements in the ectodomain of TACE, resulting in enhanced TACE substrate cleavage (Le Gall et al., 2010). This process is negatively regulated by cell surface protein disulfide isomerases, and positively regulated by the externalization of phosphatidylserine (PS) to the outer leaflet of the plasma membrane (Düsterhöft et al., 2013, Sommer et al., 2016).

A complication in envisaging how stimuli are transduced to TACE is the conflicting evidence for the importance of the TACE cytoplasmic tail. The cytoplasmic tail of TACE is phosphorylated in response to shedding stimulants, but there is no consensus concerning the impact of these phosphorylation events (Fan and Derynck, 1999, Díaz-Rodríguez et al., 2002, Fan et al., 2003, Soond et al., 2005, Xu and Derynck, 2010, Scott et al., 2011, Xu et al., 2012). Indeed, several studies have shown that the TACE cytoplasmic tail is not required for TACE stimulation (Le Gall et al., 2010, Hall and Blobel, 2012), suggesting that another transmembrane protein containing a cytoplasmic tail transduces cytoplasmic stimulatory signals to the extracellular protease domain of TACE.

**Figure 1:**
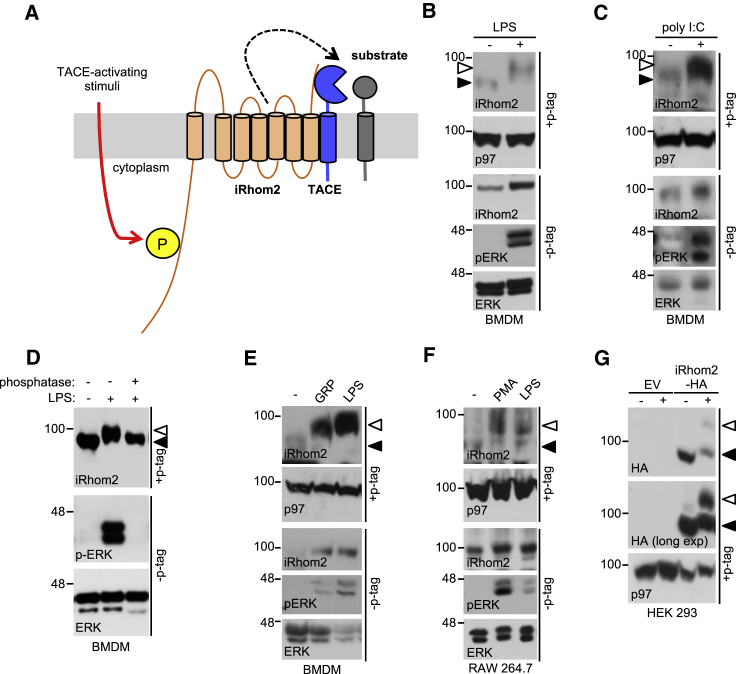
iRhom2 Is Phosphorylated in Response to TACE-Activating Stimuli (A) Schematic envisaging iRhom2 phosphorylation as a signal integrator for TACE-activating stimuli. (B and C) Phos-tag gels show that endogenous iRhom2 is phosphorylated in bone marrow-derived macrophages (BMDMs) stimulated with LPS (B) and (C) poly(I:C). (D) The mobility shift in iRhom2 is reverted by phosphatase treatment. (E) Endogenous iRhom2 is phosphorylated in BMDMs stimulated with the G protein-coupled receptor ligand gastrin-releasing peptide (GRP). (F) Endogenous iRhom2 is phosphorylated in response to PMA and LPS in RAW264.7 macrophages. (G) HA-tagged mouse iRhom2, stably expressed in HEK293ET cells, is phosphorylated in response to PMA. Throughout, a white arrowhead denotes phosphorylated iRhom2 and a black arrowhead non-phosphorylated iRhom2. p97 immunoblots are a loading control and pERK (phosphorylated ERK) a positive control for stimulation. Here and throughout, cells were stimulated for 15 min with LPS (1 μg/mL), GRP (200 ng/mL), and PMA (1 μM) and for 60 min with poly(I:C) (1 μg/mL). See also Figure S1.

iRhom2 has a long N-terminal cytoplasmic tail that is predicted to be intrinsically disordered, decorated with signatures of a signaling hub, including predicted 14-3-3 binding sites, predicted ERK (extracellular signal-regulated kinase) kinase docking sites (Roux and Blenis, 2004) (Figure S1A), and multiple potentially phosphorylated residues (Figure 1A; Table S1). Recently, Maney et al. (2015) showed that TACE activity was enhanced in cells expressing mutants of iRhom truncated within their cytoplasmic tails, but the mechanistic basis of the phenomenon was unexplored. Additional evidence for a regulatory role of the iRhom2 N terminus comes from the identification of gain of function mutations in the N terminus of human iRhom2 that enhance TACE sheddase activity (Maney et al., 2015) and cause tylosis with esophageal cancer, a keratinocyte hyperproliferative condition (Blaydon et al., 2012).

Here, we identify that phosphorylation of the iRhom2 cytoplasmic tail plays a central and essential role in the regulation of TACE stimulation.

## Results

### iRhom2 Is Phosphorylated in Response to Physiological TACE-Activating Stimuli

To test the hypothesis that iRhom2 is an essential co-factor for TACE stimulation (Figures 1A and S1A), we analyzed iRhom2 phosphorylation in response to a panel of physiological TACE-activating stimuli. To detect phosphorylation of iRhom2, we supplemented SDS-PAGE gels with Phos-tag, a compound that slows the electrophoretic mobility of phosphorylated proteins. As shown in Figures 1B and 1C, treatment of cells with the Toll-like receptor ligands lipopolysaccharide (LPS) and poly(I:C), stimuli that provoke the shedding of TNF, induced phosphorylation of endogenous iRhom2 in primary murine bone marrow-derived macrophages. Phosphatase treatment of lysates from LPS-stimulated macrophages confirmed that the slower migration of iRhom2 was indeed caused by phosphorylation (Figure 1D). We next looked at activation of a G protein-coupled receptor, a pathway linked to EGFR transactivation (Wetzker and Böhmer, 2003). We found that the gastrin-releasing peptide receptor, which is implicated in EGFR-associated cancers, also triggered iRhom2 phosphorylation (Figure 1E). The phorbol ester PMA, a stimulus commonly used to trigger TACE-mediated substrate shedding, also induced endogenous iRhom2 phosphorylation in RAW264.7 macrophages (Figure 1F) and HEK293ET cells stably expressing HA-tagged mouse iRhom2 (Figure 1G). In summary, iRhom2 is phosphorylated in response to range of physiological stimuli known to rapidly induce TACE sheddase activity.

### iRhom2 Phosphorylation Is Dependent on MAP Kinases

**Figure 2:**
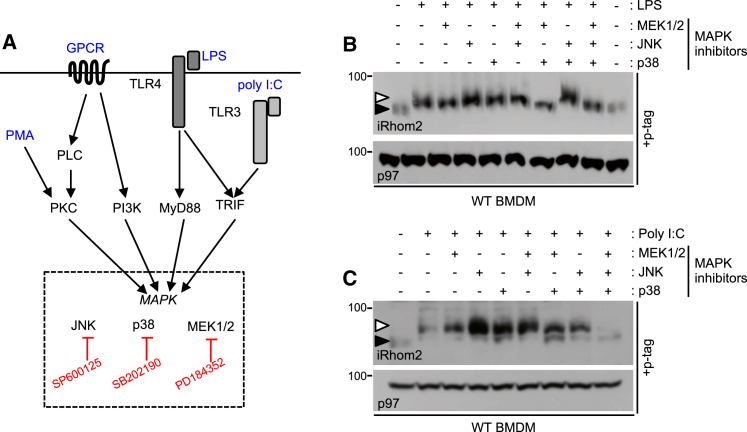
iRhom2 Phosphorylation Depends on MAP Kinases Downstream of Toll-like Receptor Signaling (A) Schematics illustrating how signaling downstream of TLR3/4, GPCRs, and PMA converges on MAPK activation. Inhibitors are highlighted in red. Adapted from Goldsmith and Dhanasekaran, 2007, Steinberg, 2008, and Oda and Kitano (2006). (B) A combination of ERK- and p38-MAPK inhibitors prevent endogenous iRhom2 phosphorylation in LPS-stimulated BMDMs. (C) MAPK inhibitors (MEK1/2, JNK, and p38) prevent endogenous iRhom2 phosphorylation in poly(I:C)-stimulated BMDM. Throughout, cells were pre-treated for 30 min with inhibitors (2.5 μM PD184352 [MEK1/2 inhibitor], 5 μM SP600125 [JNK inhibitor], 1 μM SB 202190 [p38 inhibitor]) followed by LPS (15 min) or poly(I:C) (60 min) treatment.

We next addressed which kinases were potentially involved in iRhom2 phosphorylation. The major part of iRhom2 that is exposed to the cytoplasm, accessible to kinases, is its extended N-terminal cytoplasmic tail (Figure 1A). As MAP kinases can be activated by the TACE stimuli (Díaz-Rodríguez et al., 2002, Yamamoto et al., 2002, Scott et al., 2011, Czepielewski et al., 2012) that we here show trigger iRhom2 phosphorylation (Figure 1), we focused on this kinase family. LPS activation of Toll-like receptor-4 converges on MAP kinases (Figure 2A). To examine the potential role of these kinases in iRhom2 phosphorylation, we used a pool of inhibitors targeting MEK1/2, whose downstream kinase targets are ERK1/2, plus inhibitors for JNK or p38 (Figures 2A and 2B). Although no single inhibitor blocked phosphorylation, a combination of p38 and MEK1/2 inhibitors abolished LPS-induced phosphorylation of endogenous iRhom2 (Figure 2B). In contrast, inhibitors of all three MAPK were required to block iRhom2 phosphorylation in response to Toll-like receptor-3 activation by poly(I:C) (Figure 2C). We conclude that MAP kinases are required for phosphorylation of endogenous iRhom2 in response to Toll-like receptor activation, stimuli that promote TNF shedding.

### Rapid Stimulation of TACE Shedding Requires Phosphorylation of iRhom2

In contrast to the slow rate of TACE trafficking from the ER to the Golgi (Schlöndorff et al., 2000), TACE stimulation occurs with rapid kinetics, potentially acting mechanistically to control rearrangements in the extracellular domains of TACE or the access of TACE’s active site to substrates (Le Gall et al., 2010). We next investigated the impact of iRhom2 phosphorylation on its ability to regulate TACE activity. Notably, several high throughput phosphoproteomic studies detected phosphorylated residues (Table S1) within the iRhom2 cytoplasmic tail, triggered in response to LPS and to G protein-coupled receptor agonists known to stimulate TACE activity (Christensen et al., 2010, Weintz et al., 2010). As these phosphorylation events were not confirmed biochemically, nor was their functional relevance explored, we examined this in depth.

**Figure 3:**
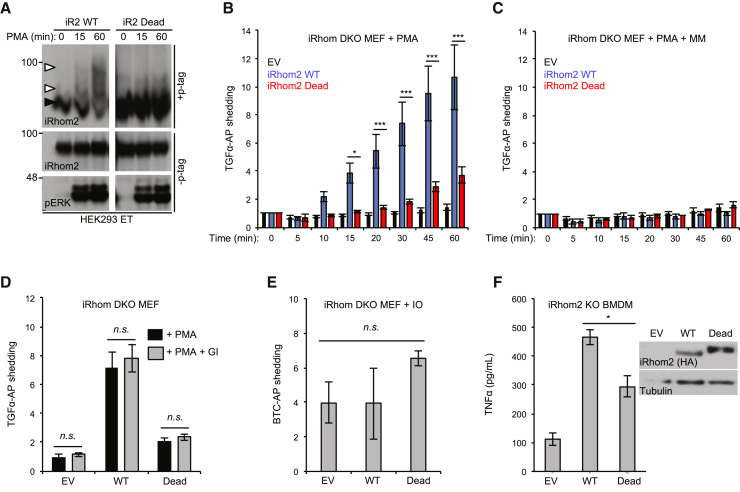
iRhom2 Cytoplasmic Tail Phosphorylation Is Required for Rapid Induction of TACE-Dependent Shedding (A) iRhom2 Dead mutant stably expressed in HEK293ET cells is not phosphorylated in response to PMA. (B) PMA-stimulated TGF-α-AP shedding requires iRhom2 phosphorylation. iRhom1/2 DKO MEFs were transduced with iRhom2 WT, iRhom2 Dead mutant, or empty vector (EV) retrovirus. (C) Marimastat (MM; 5 μM) was used to inhibit shedding by metalloproteases. (D) The ADAM10-specific inhibitor GI254023X (GI; 1 μM, 60 min) did not affect TGF-α-AP shedding in rescue assays in iRhom DKO MEFs expressing WT iRhom2 versus the Dead mutant or empty vector. (E) Ionomycin (IO; 2.5 μM, 60 min) stimulated shedding of betacellulin-AP (BTC-AP) was unaffected by iRhom2 phosphorylation. (F) iRhom2 KO BMDMs transduced with iRhom2 Dead retrovirus are defective in the shedding of TNF after 3 hr of LPS stimulation. Data are presented as mean ± SEM. See also Table S1.

iRhom1/iRhom2 double-knockout (DKO) mouse embryonic fibroblasts (MEFs) are devoid of TACE activity and cannot support stimulated TACE shedding (Christova et al., 2013). To examine the functional impact of blocking iRhom2 phosphorylation on the regulation of TACE, we constructed a compound mutant in which all 15 putative phosphorylation sites identified by the phosphoproteomic screens (Table S1) were mutated to alanine to block the ability of the protein to be phosphorylated. This phosphorylation-dead mutant (hereafter referred to as iRhom2 Dead for simplicity) could not be phosphorylated in response to PMA, indicating that the relevant sites were among the engineered mutations (Figure 3A).

To examine the ability of the iRhom2 Dead mutant to rescue TACE stimulation, we assessed its capacity to support shedding of the EGFR ligand transforming growth factor alpha fused to alkaline phosphatase (TGF-α-AP) into culture medium (Sahin et al., 2004). A time-course analysis of PMA-stimulated TGF-α-AP shedding revealed that, as expected (Le Gall et al., 2010), induction of TACE shedding occurred rapidly, within 5–20 min (Figure 3B). Notably, although TGF-α-AP shedding was rescued by expression of wild-type (WT) iRhom2, the Dead mutant was significantly impaired in its ability to rescue shedding in iRhom DKO MEFs, at all time points (Figure 3B). At the later time points, chronic exposure of cells to PMA may activate iRhom-independent shedding pathways, explaining the incomplete loss of shedding in the Dead mutant. Importantly, the effect observed was specific to TACE: TGF-α-AP shedding was blocked by the metalloprotease inhibitor Marimastat (MM) (Figure 3C) but not by the ADAM10-selective inhibitor GI254023X (Ludwig et al., 2005) (Figure 3D). Moreover, ionomycin (IO)-triggered shedding of alkaline-phosphatase fused to betacellulin (BTC), a substrate of ADAM10, the metalloprotease closely related to TACE, was unaffected by the iRhom2 Dead mutant (Figure 3E). In summary, our results reveal that iRhom2 phosphorylation is essential for the rapid activation of TACE.

We validated our findings using an alternative and physiologically relevant paradigm of TACE activation. In contrast to models of TACE shedding that rely on rapid cleavage of pre-existing substrate, in macrophages, the shedding of TNF is biologically distinct: it first requires TNF transcription and translation, in response to Toll-like receptor activation. As iRhom2 is necessary and sufficient for TACE maturation in macrophages (Adrain et al., 2012), we used retrovirus to transduce iRhom2 WT, or the Dead mutant, into primary macrophages of iRhom2 knockout (KO) mice. Similar to the MEF experiments, expression of the iRhom2 Dead mutant was less efficient in restoring LPS-triggered TNF shedding than WT iRhom2 (Figure 3F). In summary, phosphorylation of iRhom2 is crucial for the rapid stimulation of TACE activity by phorbol esters and for the more delayed release of endogenous TNF following Toll-like receptor stimulation.

### iRhom2 Phosphorylation at Ser 83 Recruits 14-3-3 Proteins, Controlling TACE Stimulation

**Figure 4:**
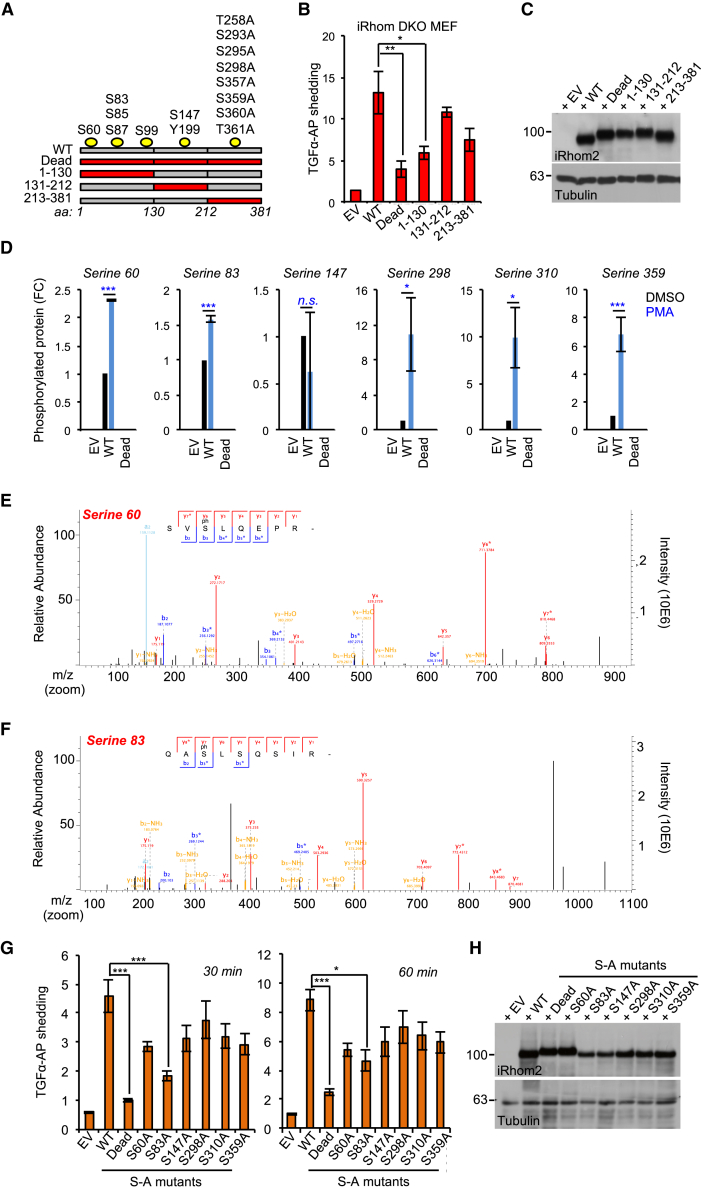
Phosphorylation of iRhom2 at Serine 83 Is Essential for TACE Shedding Activity (A) Schematic of the putative iRhom2 phosphorylated residues that were mutated to alanine. (B) The putative phosphorylated residues located within amino acids 1–130 of iRhom2 are required for TACE shedding of TGF-α-AP in rescue assays in iRhom1/2 DKO MEFs stably expressing the specified mutants. PMA (60 min) was used to stimulate shedding. EV, empty vector. (C) The iRhom2 non-phosphorylatable mutants (used in B) had equivalent expression levels in iRhom1/2 DKO MEFs. (D) Mass spectrometry analysis identified six phosphorylation sites in mouse iRhom2-HA, stably expressed in HEK293ET cells. Five of those identified are PMA inducible (15 min PMA). FC, fold change relative to WT cells treated with DMSO. n = 2, Student’s t test. (E and F) Mass spec analysis of iRhom2 phosphorylation at S60 (E) or S83 (F). (G) iRhom1/2 DKO MEFs transduced with iRhom2 retrovirus encoding the indicated serine-to-alanine (S/A) single-point mutations PMA (30 and 60 min) was used to stimulate shedding of TGF-α-AP. (H) The iRhom2 S/A non-phosphorylatable mutants (used in G) had equivalent expression levels in iRhom1/2 DKO MEFs. Data are presented as mean ± SEM.

Proteins are often phosphorylated at multiple positions, sometimes by several kinases (Cohen, 2000). To learn more about which residues within iRhom2 promote TACE sheddase activity, we deconvoluted the iRhom2 Dead compound mutant into three separate mutant clusters: 1–130, 131–212, and 213–381, on an otherwise WT, full-length iRhom2 background (Figure 4A). In TGF-α-AP-shedding assays in DKO MEFs, expression of the 1–130 mutant, which contains the N-terminal five potential phosphorylation sites, still failed to restore TACE activity, indicating that it contained the functionally relevant phosphorylation sites (Figure 4B). The 131–212 and 213–381 mutants exhibited a reduced, although not statistically significant, ability to support shedding compared with WT iRhom2, although all mutants were expressed at equivalent levels (Figure 4C).

To obtain deeper insights into the specific residues within the iRhom2 cytoplasmic tail that were phosphorylated in response to PMA in our model, we performed mass spectrometry on immunoprecipitates from HEK293ET cells expressing HA-tagged WT mouse iRhom2 versus the iRhom2 Dead mutant. This revealed that iRhom2 was phosphorylated at multiple serines upon PMA stimulation (Figure 4D), including two residues, S60 (Figure 4E) and S83 (Figure 4F), that mapped within the 1–130 mutant cluster that significantly impaired PMA-induced shedding (Figure 4B). When we examined each identified phosphorylation site individually, we found that only blocking phosphorylation at S83 significantly impaired shedding (Figure 4G). In contrast, the other mutants including S60A, impaired shedding only modestly, indicating that, at least when scrutinized individually, these residues play a minor role in shedding (Figures 4G and 4H).

**Figure 5:**
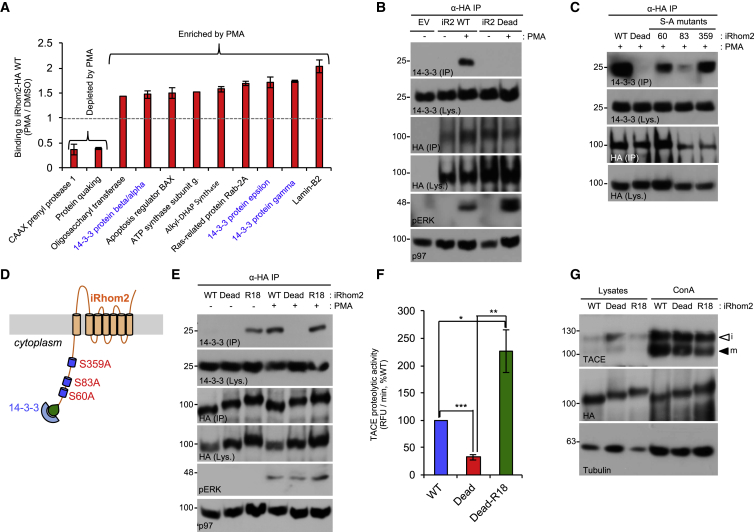
Phosphorylation-Dependent Recruitment of 14-3-3 to iRhom2 Induces Increased TACE Activity (A) iRhom2-interacting proteins with altered binding upon PMA stimulation, detected by mass spectrometry, in iRhom2-HA immunoprecipitates; 14-3-3 proteins are highlighted in blue. See Figure S1B for a more comprehensive view of this dataset (n = 2). (B) Validation of PMA-inducible 14-3-3 binding to iRhom2. HEK293ET cells expressing an empty vector (EV) control, WT, or iRhom2-Dead-HA were stimulated (PMA, 30 min). iRhom2-HA was immunoprecipitated and endogenous 14-3-3 binding detected with a pan-14-3-3 antibody. (C) S83 is the major phosphorylated residue that recruits 14-3-3 proteins. HEK293ET cells expressing iRhom2-HA serine-to-alanine (S/A) mutants at the three putative 14-3-3 binding sites (S60, S83, and S359) were stimulated with PMA (30 min), iRhom2-HA was immunoprecipitated and endogenous 14-3-3 binding detected. (D) Schematic of the R18-iRhom2 Dead mutant. The R18 peptide (PHCVPRDLSWLDLEANMCLP) was fused to the N terminus of iRhom2 Dead-HA mutant, to confer phosphorylation independent binding of 14-3-3 proteins. (E) The R18-iRhom2 Dead-HA mutant binds constitutively to endogenous 14-3-3 proteins. HEK293ET cells expressing the indicated plasmids were stimulated (PMA, 30 min). (F) 14-3-3 binding induces TACE cell surface proteolytic activity. HEK293ET cells overexpressing the indicated plasmids were left untreated. TACE proteolytic activity on the cell surface was determined by measuring its ability to cleave a fluorogenic substrate. RFU, relative fluorescent units. Student’s t test. (G) TACE maturation is not affected by 14-3-3 binding. Lysates from iRhom1/2 DKO MEFs stably expressing the indicated plasmids were ConA enriched and immunoblotted for TACE. Data are presented as mean ± SEM. See also Figure S1.

To examine the functional impact of iRhom2 phosphorylation, we performed mass spectrometry analysis to determine whether the cohort of iRhom2-interacting proteins changed upon phosphorylation. These experiments revealed 11 proteins, whose binding to iRhom2 was altered upon PMA stimulation, including several 14-3-3 protein isoforms, that function as molecular switches, whose recruitment is often contingent on phosphorylation of their binding proteins (Figures 5A and S1B). The binding of 14-3-3 proteins to iRhom2 appeared plausible because 14-3-3 recruitment motifs (Madeira et al., 2015) are found at phosphoserines S60, S83, and S359 within the iRhom2 cytoplasmic tail (Figure S1A). Co-immunoprecipitation experiments confirmed that 14-3-3 proteins were recruited to iRhom2 upon PMA stimulation (Figure 5B) dependent on phosphorylation at S83 (Figure 5C). As S83 is important for shedding (Figure 4G), this highlights a connection between the phosphorylation-dependent recruitment of 14-3-3 and TACE stimulation.

To test directly the role of 14-3-3 proteins in TACE stimulation, we constructed a mutant in which the well-characterized 14-3-3 recruitment motif R18 (Masters and Fu, 2001) was added to the N terminus of the cytoplasmic tail of the iRhom2 Dead mutant (Figure 5D). Interestingly, this mutant, which bound to 14-3-3 proteins constitutively in the absence of PMA stimulation (Figure 5E), exhibited a significantly increased capacity to facilitate TACE shedding activity (Figure 5F), without increasing the pool of mature TACE (Figure 5G). Together, our data confirm that 14-3-3 protein recruitment is central to the mechanism whereby iRhom2 phosphorylation at S83 controls TACE stimulation.

### iRhom2 Phosphorylation Does Not Control iRhom/TACE Trafficking to the Cell Surface

**Figure 6:**
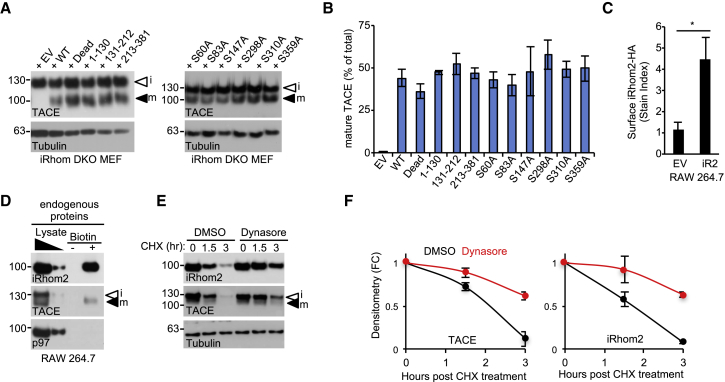
iRhom2 Phosphorylation Regulates TACE Beyond the ER (A) TACE maturation is not affected by iRhom2 phosphorylation in iRhom1/2 DKO MEFs stably expressing the indicated iRhom2 mutants. (B) Densitometric scans illustrating the proportion of mature TACE in iRhom DKO MEFs expressing the indicated iRhom2 mutants. (C) Flow cytometric detection of overexpressed cell surface iRhom2-HA in non-permeabilized RAW264.7 cells. Student’s t test. EV, empty vector. (D) Cell surface biotinylation assays detecting endogenous cell surface iRhom2 in RAW264.7 macrophages. (E) Consistent with cell surface localization, endogenous iRhom2 degradation upon treatment with a protein synthesis inhibitor (CHX, 50 μg/mL) is rescued by the inhibitor of dynamin-dependent endocytosis dynasore (80 μM, co-treatment with CHX). (F) Densitometry of (D). Data are presented as mean ± SEM. See also Figure S2 and Table S2.

The recruitment of 14-3-3 proteins can exert distinct effects on the trafficking fate of membrane proteins, including facilitating their exit from the ER, or regulating their endocytosis (O’Kelly et al., 2002, Gabriel et al., 2012). To investigate whether 14-3-3 protein recruitment to phosphorylated iRhom2 affected TACE maturation (which requires its progression into the *trans-*Golgi apparatus) or iRhom2 trafficking itself, we began by examining the ability of iRhom2 phosphorylation mutants to promote TACE trafficking to the *trans-*Golgi apparatus, where the prodomain of TACE is normally cleaved off by furin (Schlöndorff et al., 2000, Adrain et al., 2012). Notably, TACE maturation was not impaired in iRhom DKO MEFs expressing a panel of non-phosphorylatable mutants (Figures 6A and 6B), confirming that iRhom2 phosphorylation does not control TACE trafficking from the ER to the *trans-*Golgi.

Our observations indicate that iRhom2 fulfills two important, but separable, functions: its canonical role in the anterograde trafficking of TACE, versus a distinct function, dependent on iRhom2 phosphorylation, that controls the rapid stimulation of TACE activity. Assuming that the effect elicited by iRhom2 phosphorylation impinges directly on TACE, the corollary is that iRhom2 should be found on the cell surface, where TACE exerts its sheddase activity. Immunofluorescent experiments describe iRhom2 to be an intracellular, predominantly ER-localized protein (Zettl et al., 2011, Maney et al., 2015, Grieve et al., 2017). However, two recent studies have found that overexpressed forms of iRhom localize to the cell surface (Maney et al., 2015, Grieve et al., 2017). We confirmed this result in flow cytometry experiments, in unpermeabilized RAW267.4 murine macrophages stably expressing HA-tagged iRhom2 (Figure 6C). As overexpression can risk artifactual spillover of membrane proteins into other compartments, we investigated this phenomenon more stringently, using a cell-impermeable biotinylation reagent to detect endogenous iRhom2 on the cell surface (Figure 6D). Inhibiting dynamin-dependent endocytosis using dynasore blocked iRhom2 degradation (Figures 6E and 6F), supporting the hypothesis that, like TACE (Lorenzen et al., 2016), the stability of iRhom2 is controlled by the endolysosomal system.

Our observations suggest that iRhom2 hence has the potential to regulate TACE throughout the secretory pathway. However, the amounts of cell surface iRhom2 and TACE appeared equivalent in cells expressing WT versus the Dead mutant (Figures S2A–SC), suggesting that iRhom2 phosphorylation does not control the trafficking of iRhom2 or TACE to the cell surface. Moreover, the rate of PMA-triggered loss of TACE from the cell surface was not significantly different (Figures S2A and S2B), and dynasore did not rescue shedding in the iRhom2 Dead mutant, confirming that defective endocytosis or sorting to the lysosome could not account for the TACE shedding defect (Figure S2D).

An important implication of these results is that the phosphorylation mutants of iRhom2 are not misfolded, because the iRhom2 Dead mutant was fully capable of supporting the trafficking of itself and TACE throughout the secretory pathway. By contrast, if iRhom2 phosphorylation mutants were misfolded, their retention in the ER and degradation by ER-associated degradation (ERAD) would cause the loss of their essential TACE trafficking function, blocking TACE maturation. Further evidence that the iRhom2 Dead mutant is properly folded came from mass spectrometry analysis of iRhom2 interacting proteins, revealing that, apart from 14-3-3 proteins (as anticipated), the majority (>93%) of proteins that interact with WT iRhom2 also bound the iRhom2 Dead mutant (Table S2; Figures S1C and S1D). Hence, blocking iRhom2 phosphorylation causes a specific defect in the biology of TACE stimulation, without affecting the biogenesis or trafficking of iRhom2 and TACE.

### iRhom2 Phosphorylation Enhances TACE Activity Independently of Substrate Recruitment by Promoting Dissociation of TACE from iRhom2

**Figure 7:**
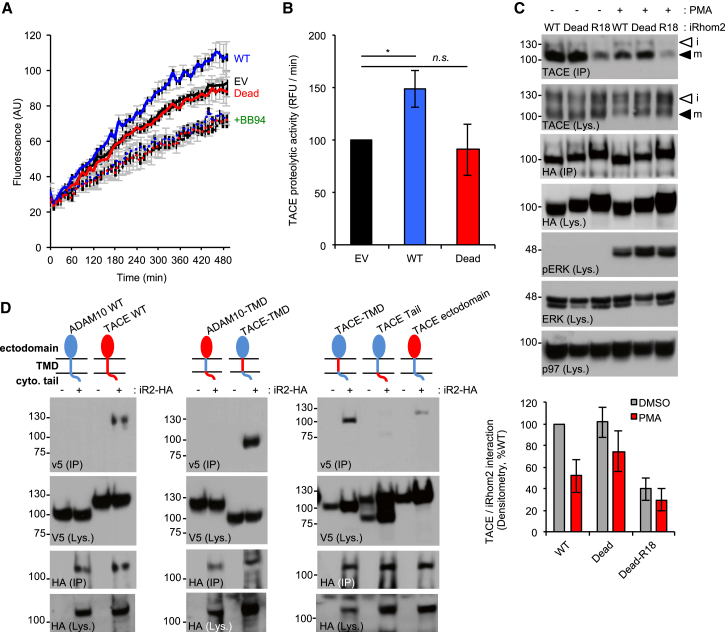
iRhom2 Phosphorylation Regulates TACE Proteolytic Activity at the Cell Surface Independently of Substrate Delivery (A) iRhom2-HA WT overexpression in HEK293ET cells, but not iRhom2-HA Dead, increases the ability of TACE to cleave a fluorogenic substrate added to the culture media of PMA-stimulated cells. The metalloprotease inhibitor Batimastat (BB94) demonstrates the component of TACE activity conferred by iRhom2 overexpression. AU, arbitrary units. (B) TACE cell surface proteolytic activity expressed as the rate of peptide cleavage (relative fluorescent units [RFU] per minute). Student’s t test. (C) 14-3-3 binding induces dissociation of the iRhom2/TACE complex. HEK293ET cells expressing the indicated plasmids were stimulated (PMA, 30 min). Dissociation of iRhom2/TACE was assessed by α-HA immunoprecipitates. Bottom: densitometric quantification of the interaction between TACE and iRhom2, as a percentage of binding to iRhom2 WT under non-stimulated conditions. (D) Plasmids encoding WT V5 tagged TACE, ADAM10, or the indicated domain swap chimeras of TACE (red) and ADAM10 (blue) were transfected into HEK293ET together with iRhom2-HA (+) or empty vector (−). Binding of TACE, ADAM10, or chimeric constructs to iRhom2-HA was detected using anti-V5 antibody on anti-HA IPs. Data are presented as mean ± SEM. See also Figure S3.

As previously proposed (Lemberg and Adrain, 2016), our model implies that iRhom2 remains associated with TACE at the cell surface. As it has been suggested that stimulation controls exposure of TACE’s active site (Le Gall et al., 2010), we hypothesized that iRhom2 phosphorylation may be the mechanism that facilitates TACE stimulation by exposing the TACE proteolytic site to its substrates. To test this, we used an assay using a fluorogenic TACE substrate added to the media of PMA-treated cells. The assay specifically measured TACE activity upon iRhom2 overexpression, because a TACE-specific short hairpin RNA (shRNA) abrogated peptide cleavage, whereas knockdown of ADAM10, TACE’s closest relative, did not affect peptide hydrolysis (Figures S3A and S3B). Moreover, the assay was sensitive to inhibition by the metalloprotease inhibitor BB94, but not GI254023X, which has a 100-fold preference for ADAM10 over TACE (Figures S3C and S3D). Compared with cells expressing WT iRhom2, TACE activity in cells expressing the iRhom2 Dead mutant were defective in stimulated TACE proteolytic activity (Figures 7A and 7B). This is significant because it rules out the possibility that iRhom2 phosphorylation is required for the recruitment of transmembrane TACE substrates.

To assess the basis for how phosphorylation of iRhom2 rendered TACE more accessible to substrates in more detail, we focused on the iRhom2 Dead mutant containing the “R18” 14-3-3 recruitment motif, which constitutively binds to 14-3-3 (Figure 5E) and rescues the loss of function phenotype associated with the iRhom2 Dead mutant (Figure 5F). Similar to a recent observation that iRhom phosphorylation promotes dissociation of TACE from iRhom2 on the cell surface (Grieve et al., 2017), we observed substantially reduced binding of mature TACE to the R18-iRhom2 Dead mutant (Figure 7C). We also observed a trend suggesting that the Dead mutant retained TACE more efficiently than WT iRhom2 in response to PMA stimulation (Figure 7C).

We next focused on the mechanism whereby 14-3-3 recruitment triggers dissociation of TACE from iRhom2. As Grieve et al. (2017) proposed that the cytoplasmic tail of iRhom2 was specifically required for the binding to mature TACE, we examined which portions of TACE are needed for interaction with iRhom2 in co-immunoprecipitations. Significantly, we found that the TACE transmembrane domain was the principal determinant of binding to iRhom2: replacing the ADAM10 transmembrane domain with that of TACE conferred binding of this chimeric construct to iRhom2 (Figure 7D). By contrast, an ADAM10 chimera containing the TACE cytoplasmic tail could not bind to iRhom2, implying, albeit indirectly, that the iRhom2 tail does not play a major role in binding to the TACE tail (Figure 7D). Together, we conclude that iRhom2 phosphorylation recruits 14-3-3 proteins, enforcing dissociation of iRhom from TACE, via a mechanism dependent on transmembrane domain interactions, and rendering the active site of TACE more accessible to its substrates.

## Discussion

Our data highlight the important regulatory role of the iRhom cytoplasmic tail and suggests that it is an important signaling hub. It is interesting to note that the iRhom2 cytoplasmic tail contains predicted disordered segments (Figure S1A). Such regions are often rich in protein interaction surfaces, including 14-3-3 binding sites, and are highly sensitive to input from signaling pathways, including phosphorylation-induced conformational changes (Bozoky et al., 2013).

During the revision of our manuscript, Grieve et al. (2017) published findings that reach similar conclusions to ours, although our data differ from, and extend, those observations in several ways. Our work establishes a physiological context in which iRhom phosphorylation occurs, placing it within the settings of infection (activation of Toll-like receptors by bacteria and viruses) and cancer (EGFR transactivation by G protein-coupled receptors). Our objective identification of the phosphorylation sites within iRhom2 is complementary to Grieve et al. (2017): whereas our work maps the phosphorylated residues induced upon TACE stimulation in mouse iRhom2, Grieve et al. (2017) documented basal phosphorylation of residues within human iRhom2.

Grieve et al. (2017) propose that the cytoplasmic tail of iRhom2 interacts with mature TACE. Arguing against this, our data show that the transmembrane domain of TACE is necessary and sufficient for binding to iRhom2, whereas the TACE cytoplasmic tail is not. Moreover, although the TACE cytoplasmic tail is dispensable for stimulation, its transmembrane domain cannot be substituted (Le Gall et al., 2010); single-point mutants within TACE transmembrane domain impair stimulated shedding (Li et al., 2017). Our interpretation is that recruitment of 14-3-3 proteins to the iRhom2 tail triggers a cue that is transduced through iRhom2, to weaken transmembrane interactions between TACE and iRhom2, releasing TACE from the complex, facilitating its access to substrates.

It is important to relate our observations to the mechanism of TACE stimulation proposed by Sommer et al. (2016), who showed that the membrane proximal domain of TACE has an affinity for the phospholipid PS. TACE-activating stimuli trigger PS externalization; the TACE membrane proximal domain binds PS, provoking a conformational change, drawing TACE in closer juxtaposition to its substrates (Sommer et al., 2016). Notably, as blocking this PS-dependent mechanism impairs the cleavage of transmembrane TACE substrates, but does not affect peptide substrate hydrolysis (Sommer et al., 2016), it implies that our iRhom-dependent mechanism is distinct from this. An additional potential mechanism that has been proposed to explain the stimulation mechanism involves the stimulus-induced dissociation of the metalloprotease inhibitor TIMP3 from TACE. However, as the extent of TACE stimulation is identical in TIMP3 null cells as in WT cells (Le Gall et al., 2010), we consider this possibility unlikely.

There is evidence that iRhoms can govern the substrate specificity of TACE (Maretzky et al., 2013), and Grieve et al. (2017) noted the possibility that phosphorylation of iRhom2 could influence substrate selection. We do not rule out the possibility that some of the multiple phosphorylation sites within iRhoms may influence substrate recruitment. However, because our phosphorylation-defective iRhom2 Dead mutant cannot support TACE cleavage of a peptide substrate, it suggests that a failure to recruit transmembrane substrate to TACE is not the basis of the defect.

Our observation that iRhom2 is at the core of the cell surface shedding regulatory machinery reconciles the ambiguity that TACE stimulation involves p38 and ERK kinases (Díaz-Rodríguez et al., 2002, Fan et al., 2003, Scott et al., 2011, Soond et al., 2005, Xu et al., 2012, Fan and Derynck, 1999), yet its tail is dispensable for stimulation (Le Gall et al., 2010, Hall and Blobel, 2012). Consistent with a potential role for MAP kinases, the cytoplasmic tail of iRhom2 contains two predicted ERK-D boxes (Figure S1A). Similarly, our immunoprecipitation and mass spectrometry experiments (Figure S4) detected the binding of kinases to iRhom1 and iRhom2, including ERK1, ERK2, and p38α, as well as binding of RSK1 and RSK2 to iRhom2. However, we cannot conclude that direct phosphorylation of iRhom2 by MAP kinases is key to the TACE stimulation mechanism. From our analyses, the single most important residue in iRhom2 for stimulated shedding is serine 83, which harbors the site [K/R]-XX-[S/T] recognized by the AGC kinase family members, such as RSK kinases, that are activated downstream of ERK/p38 (Roux and Blenis, 2004, Pearce et al., 2010). iRhom2 is phosphorylated in multiple residues; presumably several phosphorylation sites collectively make additive contributions, as demonstrated by the iRhom2 Dead mutant, whose function is more profoundly blocked than the S83A point mutant. Overall, we do not exclude the possibility that phosphorylation of iRhom2 by several kinase families control stimulated TACE shedding.

Notably, our mass spectrometry experiments identified that several residues within iRhom2, including S83, exhibit a degree of basal phosphorylation in the absence of stimulation (Figure 4D). This raises the possibility that the basal activity of TACE may also be regulated by iRhom2 phosphorylation.

Our observation that iRhom2 is phosphorylated is an example of the dynamic regulation of rhomboid like proteins in the secretory pathway by signaling pathways. A prior belief, from studies that demonstrated that expression of rhomboid proteases prefigures signaling (Wasserman and Freeman, 1998), was that rhomboid proteins are not subject to post-transcriptional regulation. However, our work reveals that iRhoms are highly regulated molecules. Interestingly, there are many parallels between the regulation of iRhoms by their cytoplasmic tails and the regulation of the important chloride channel, cystic fibrosis transmembrane conductance regulator (CFTR). Like iRhom, CFTR contains a cytoplasmic loop called the “R domain” (regulatory domain) that is highly disordered, contains sites for phosphorylation and 14-3-3 binding, and plays a key role in regulating CFTR ion channel activity (Bozoky et al., 2013). Because of these striking similarities, we propose to name the cytoplasmic tail of iRhoms the regulatory (R) domain (Figure S5). Interestingly, the CFTR R domain has been proposed to act as signaling hub not only for pathways that regulate CFTR but as a general signaling platform. It will be interesting to determine whether the same applies for iRhoms.

The discovery that endogenous iRhom2 localizes to the cell surface, and that its phosphorylation is required for shedding, requires rethinking of the model for iRhom function. Rather than a trafficking role restricted to the ER-to-Golgi transport of TACE, the data now support a model whereby iRhom and TACE traffic to the cell surface as a complex, allowing iRhom to fulfill a role in sensing and transducing shedding stimuli to TACE (Figure S5). Rather than acting as a TACE trafficking factor, iRhom’s role could be regarded as analogous to the relationship between the accessory subunits of multi-membrane protein complexes, for example, gamma secretase, the T cell receptor, or MHC class I. Similar to the examples above, loss of iRhom results in the ER retention of TACE. Additionally, iRhom could be viewed as an allosteric regulator of the TACE complex.

## Experimental Procedures

### Mice

Experiments with mice were performed in accordance with protocols approved by the IGC Ethics Committee and the National Entity Direção Geral de Alimentação e Veterinária, in accordance with the Portuguese (Decreto-Lei no. 113/2013) and European (directive 2010/63/EU) legislation related to housing, husbandry, and animal welfare. iRhom2^−/−^ (KO) mice were previously reported (Adrain et al., 2012).

### Phos-tag Gels and Protein Dephosphorylation

Phos-tag gels were performed as described in Supplemental Experimental Procedures.

### Mass Spectrometry Analysis of iRhom2 Phosphorylation and Phosphorylation-Dependent Interactome

HEK293ET cells expressing empty vector, iRhom2-HA WT, or iRhom2 Dead-HA were serum-starved overnight and stimulated with PMA (1 μM) for 15 min. Immunoprecipitations, sample digestion, and mass spectrometry are described in Supplemental Experimental Procedures.

### Cell Surface Biotinylation

Biotinylation was performed as previously described for bone marrow-derived macrophages (BMDMs) (Adrain et al., 2012) with small modifications, detailed in Supplemental Experimental Procedures.

### Flow Cytometry

RAW264.7 macrophages (1 × 10^6^) expressing empty vector or iRhom2-HA were detached with a cell scraper, Fc-blocked, and stained with an anti-HA primary antibody and Alexa 633-labeled secondary antibody. HEK293ET cells expressing iRhom2-HA WT or iRhom2 Dead-HA were stained with a phycoerythrin-conjugated anti-TACE primary antibody or isotype control phycoerythrin-conjugated IgG1. Analysis details are provided in Supplemental Experimental Procedures.

### TACE Enzymatic Assay on Live Cells

The TACE enzymatic assay used an internally quenched fluorogenic peptide based on the TNF cleavage site. Full experimental details are provided in Supplemental Experimental Procedures.

### Shedding Assays

MEFs were transfected with plasmids encoding alkaline phosphatase-tagged EGFR ligands TGF-α and BTC (Sahin et al., 2006), as described in Supplemental Experimental Procedures.

### Statistical Methods

Unless otherwise stated, all experiments were performed at least three times, and statistical significance was determined using one-way ANOVA with Tukey’s multiple comparison testing. Asterisks indicate statistical significance: ^∗^p < 0.05, ^∗∗^p < 0.01, and ^∗∗∗^p < 0.001.

## Author Contributions

M.C., I.O., A.K., and C.A. designed experiments. M.C., I.O., E.B., C.J.G., C.G., M.B., T.H., A. Bolado, A. Bileck, A.K., I.F., and P.M.D. carried out experiments and interpreted results. M.C., I.O., and C.A. contributed to the overall design of the study. M.C. and C.A. wrote and all authors edited the manuscript. C.A. supervised the project.

## Supplemental Information

Document S1. Supplemental Experimental Procedures, Figures S1–S5, and Tables S1 and S2

Document S2. Article plus Supplemental Information
