# Supplementary material for: Phosphorylation of iRhom2 Controls Stimulated Proteolytic Shedding by the Metalloprotease ADAM17/TACE
Source: Cell Rep. 2017 Oct 17;21(3):745–57. doi: 10.1016/j.celrep.2017.09.074 (PMC5656746; doi:10.1016/j.celrep.2017.09.074)
Supplement: Document S1. Supplemental Experimental Procedures, Figures S1–S5, and Tables S1 and S2 [file mmc1.pdf]

Cell Reports, Volume *21*

Supplemental Information

Phosphorylation of iRhom2 Controls Stimulated Proteolytic Shedding by the Metalloprotease ADAM17/TACE

Miguel Cavadas, Ioanna Oikonomidi, Catarina J. Gaspar, Emma Burbridge, Marina Badenes, Inês Félix, Alfonso Bolado, Tianyi Hu, Andrea Bileck, Christopher Gerner, Pedro M. Domingos, Alex von Kriegsheim, and Colin Adrain

Supplemental Figures

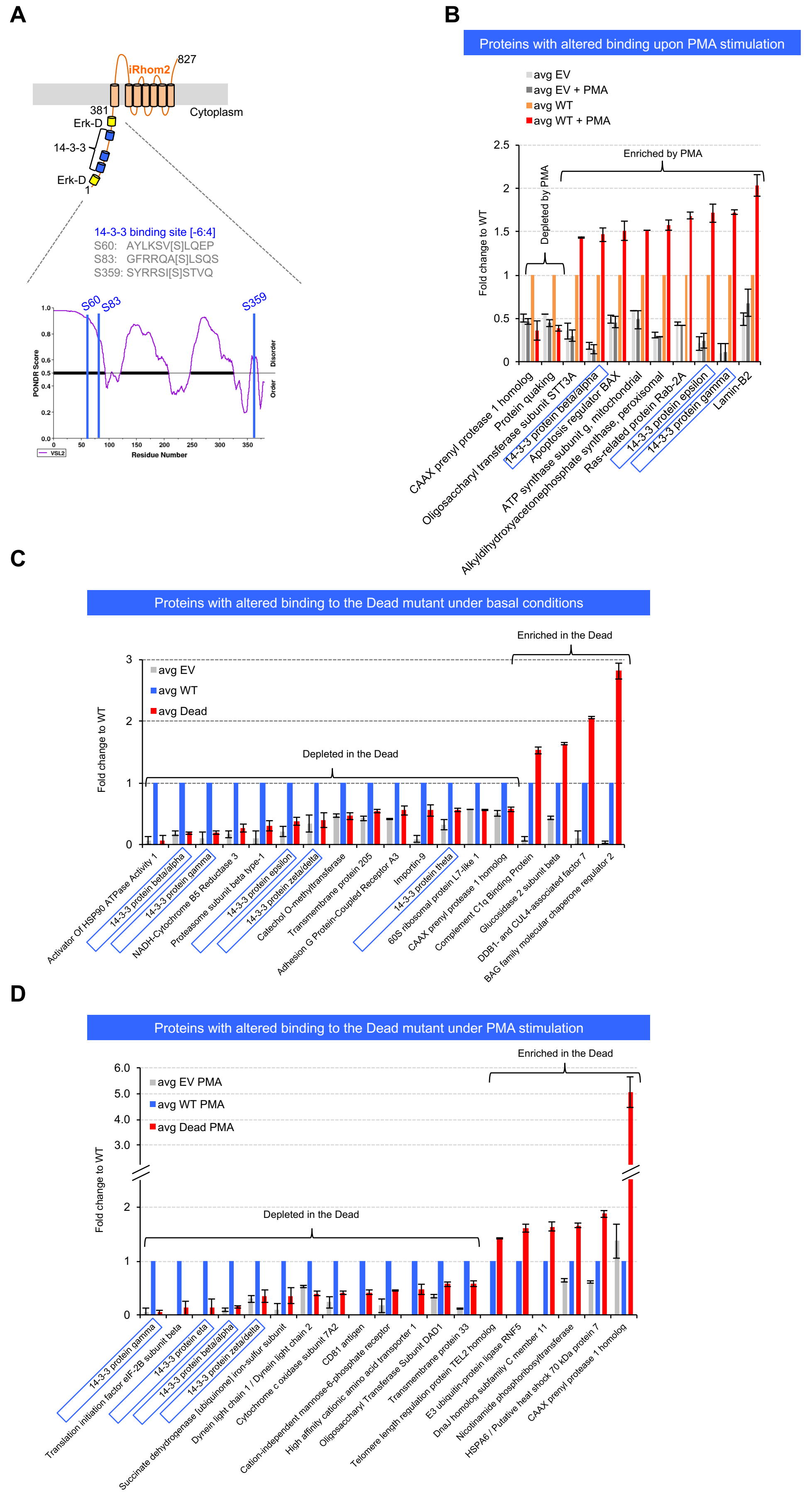

Figure S1. Features within the iRhom2 N-terminus and PMA-stimulated iRhom2-interacting proteins identified by immunoprecipitation/mass spectrometry. Related to Figures 1 and 5. (**A**) The iRhom2 N-terminus has a predicted highly disordered character and contains two predicted ERK docking sites (ERK-D domains), indicated in yellow (aa 19-29 and 334-345). The iRhom2 N-terminus also contains three predicted 14-3-3 binding sites, indicated in blue. Several large-scale phosphoproteomic studies have identified phosphopeptides within the iRhom2 N-terminus, (see Experimental procedures and Table S1 for the specific residues). Several phosphopeptides have been identified, but the kinases responsible are unknown, nor are the functional implications. Details of the prediction algorithms used to characterize features in the iRhom N-terminus are described in Experimental Procedures. (**B**) iRhom2-interacting proteins with altered binding upon PMA stimulation. 14-3-3 proteins are highlighted by a blue box. αHA immunoprecipitates from HEK293 ET cells expressing HA tagged iRhom2-WT or empty vector (EV), were subjected to mass-spec analysis. Interacting proteins whose binding to iRhom2 WT was statistically significantly increased (t-test) over background binding to the immunoprecipitation beads are shown. To increase the stringency, only changes greater than 40% were considered. The same criterion was used to define the interacting proteins whose binding was altered upon PMA stimulation. Proteins with altered binding to the iRhom2 Dead mutant, compared to WT iRhom2 WT, under basal (**C**) and PMA stimulated (**D**) conditions. 14-3-3 proteins are highlighted by a blue box. Anti-HA immunoprecipitates from HEK293 ET cells expressing HA tagged iRhom2 WT or iRhom2 Dead, or empty vector (EV), were subjected to mass-spec analysis. Interacting proteins whose binding to the iRhom2 Dead mutant was enriched or depleted over the WT are shown (using the same criteria as described in the legend of Figure S1B, only hits that were statistically significant and changed at least 40% were considered). Of note, some proteins that were not detected in the WT above the EV levels, were highly enriched in the iRhom2 Dead mutant immunoprecipitates. To identify these hits, datasets were interrogated for proteins with increased binding to the iRhom2 Dead compared to the WT and EV. Throughout, data are presented as mean ± s.e.m. n=2.

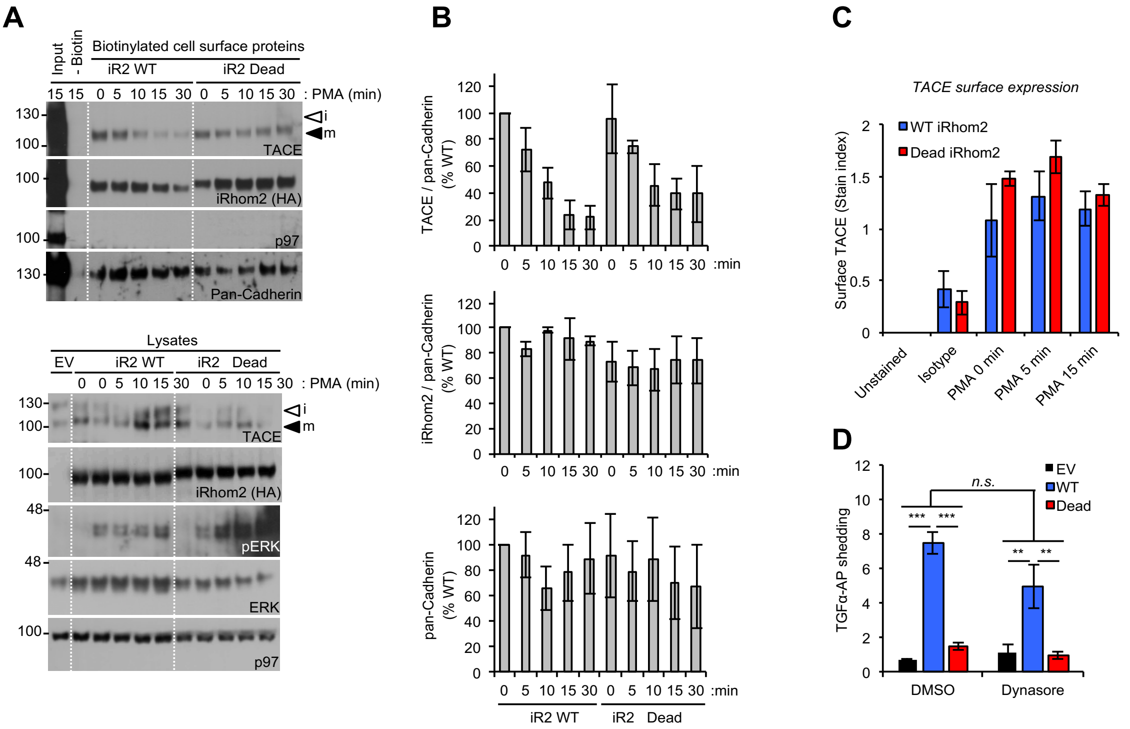

Figure S2. Cell surface expression and PMA induced internalization of TACE are not affected by iRhom2 phosphorylation. Related to Figure 6. (**A**) Biotinylation assays, show that iRhom2 phosphorylation did not affect PMA (1 μM) induced internalization of TACE, in HEK293 ET cells stably expressing iRhom2-HA WT or Dead mutant. (**B**) Densitometry of TACE, iRhom2 and pan-Cadherin bands from 4 replicates of the experiment shown in (A). (**C**) Flow cytometry of non-permeabilized HEK293 ET overexpressing iRhom2-HA WT and Dead constructs to assess cell surface levels of TACE. Cells were stimulated with PMA (1 μM for the indicated periods). (**D**) Defective TGFα-AP shedding in DKO MEFs expressing the iRhom2 Dead mutant is not rescued by the dynamin-dependent endocytosis inhibitor, dynasore. *p<0.05, **p<0.01, ***p<0.001; one-Way ANOVA followed by Tukey's multiple comparisons test. Data are shown as mean ± s.e.m, of at least 3 independent experiments.

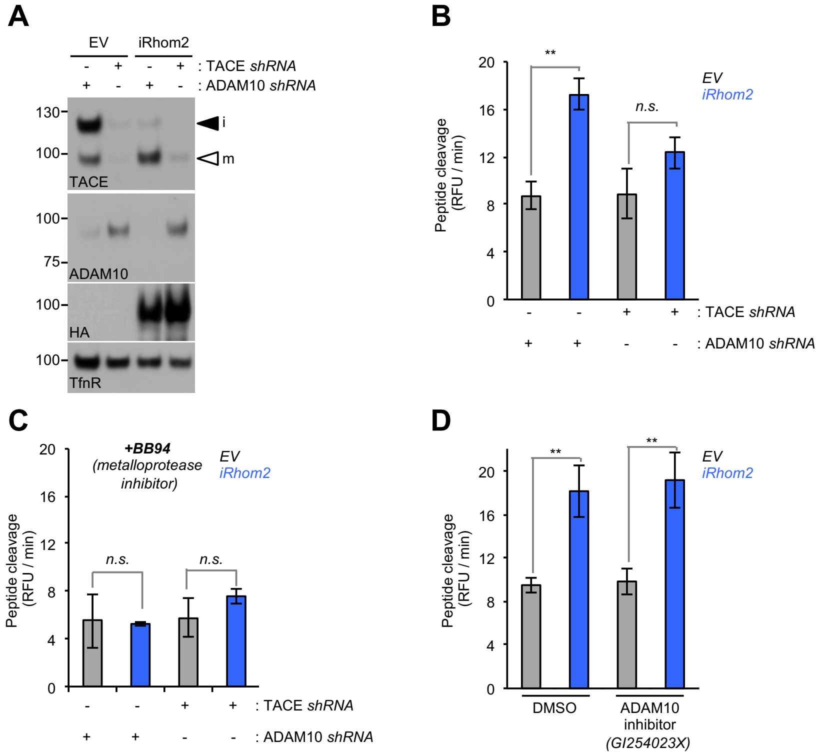

Figure S3. TACE, but not ADAM10, is required for iRhom2-dependent cleavage of a fluorogenic TACE peptide substrate on the cell surface. Related to Figure 7. (**A**) The knockdown of TACE and ADAM10 by lentivirus-delivered shRNA, in HEK293 ET cells expressing empty vector or overexpressing iRhom2-HA, was confirmed by western blotting of ConA enriched cell lysates. The transferrin receptor (TfnR) was used as loading control. (**B**) iRhom2-HA WT overexpression in HEK293 ET cells increases the cleavage of a fluorogenic TACE peptide substrate. The effect is unimpaired by shRNA-mediated knockdown of ADAM10 but is abrogated by knockdown of TACE. (**C**) The metalloprotease inhibitor Batimastat (BB94) inhibits the increased peptide hydrolysis caused by iRhom2-HA overexpression. (**D**) The ADAM10-selective inhibitor GI254023X had no effect on iRhom2 induced peptide cleavage. In B-D, the peptide was added to the culture media of PMA (1 μM) stimulated cells in the presence of BB94 (10 μM) or GI254023X (1 μM) where indicated. Peptide cleavage was expressed as the rate of peptide cleavage (RFU/minute). Data are presented as mean ± s.e.m. **P<0.01, *n.s.* not significant; student's t-test. n =3 independent experiments.

A

	Kinase motif	Kinase group	Kinase family
mouse S60	MLPERRKNPAYLKSV**S**LQEPRGRWQEGAEKR		
	S/T-X-X-E	CMGC	CK2
	K/R-X-X-S/T	AGC	
mouse S83	WQEGAEKRPGFRRQA**S**LSQSIRKSTAQWFGV		
	S-X-X-X-S	CMGC	GSK
	R-X-X-S/T	CAMK	CAMK2
	K/R-X-X-S/T	AGC	
mouse S147	LELPSQEVPSFQGTE**S**PKPCKMPKIVDPLAR		
	S/T-P-K/R	CMGC	CDK
	K/R-X-X-S/T	AGC	
mouse S298	SASYFRGVPHSASPV**S**PDGVHIPLKEYSGGR		
	S-X-X-S/T	CK1	CK1
	P-X-S/T-P	CMGC	MAPK
mouse S310	SPVSPDGVHIPLKEY**S**GGRALGPGTQRGKRI		
	K/R-X-X-S/T	AGC	
mouse S359	GVVGNWLNRSYRRSI**S**STVQRQLESFDSHRP		
	R-X-X-S/T	CAMK	CAMK2
	R-R/S/T-X-S/T-X-S/T	AGC	AKT

B

Kinase group	AGC			CMGC		
Kinase family	RSK		GRK	MAPK	MAPK	MAPK
Protein	RSK1	RSK2	GPCR kinase 6	ERK1	ERK2	p38α
Gene	RPS6KA1	RPS6KA3	GRK6	MAPK3	MAPK1	MAPK14
EV	-	-	-	-	-	-
iR1	+	-	-	+	+	+
iR2	+	+	+	+	+	+

Figure S4. iRhom2 interacts with MAPK and AGC-family kinases. Related to Discussion. (**A**) Kinase motifs present at each phosphorylation site identified in PMA-stimulated HEK293 ET iRhom2-HA expressing cells. (**B**) Kinases interacting with iRhom1 and iRhom2. Data was obtained from mass-spec analysis of immunoprecipitations from HEK293 ET cells expressing HA-tagged iRhom1, iRhom2 versus empty vector (EV) as negative control for binding.

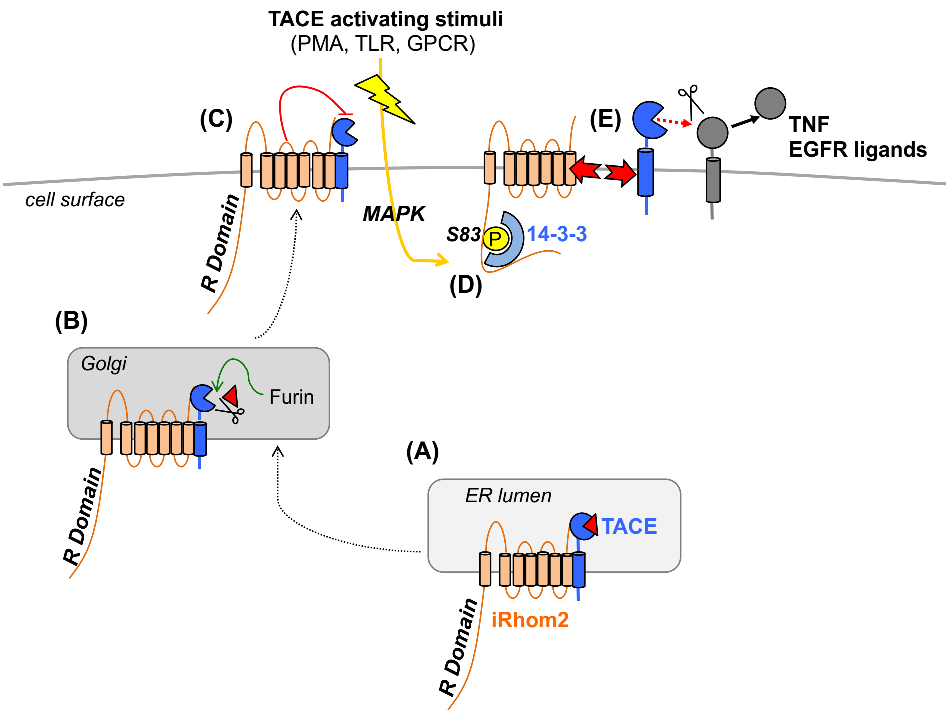

Figure S5. iRhom2 regulates TACE via two distinct mechanisms. Related to Discussion. The schematic depicts iRhom2 with an extended, disordered N-terminal cytoplasmic tail. In light of its important regulatory properties, we propose to name this module the regulatory domain (R domain). Our previous work (Adrain et al., 2012) showed that iRhom2 is essential for the trafficking of TACE from the ER (**A**) to the *trans*-Golgi apparatus, where the proprotein convertase Furin cleaves off TACE's inhibitory prodomain, rendering TACE basally active (**B**). iRhom2 and TACE then traffic to the cell surface (**C**) where the interaction with iRhom2 ensures that TACE has only basal proteolytic activity because of reduced access to its substrates. Under stimulation conditions (e.g., PMA, GPCR activation, or TLR signaling) kinases phosphorylate iRhom2 within its R domain (**D**), which triggers the recruitment of 14-3-3 proteins to the iRhom2/TACE complex. 14-3-3 binding to the R domain of iRhom2 leads to dissociation of the transmembrane helices of the iRhom2/TACE complex (**E**). This enables increased TACE cleavage of its substrates, potentially mediated by increased exposure of TACE to its substrates upon detachment from iRhom2; dissociation may also induce conformational changes in TACE's extracellular domains that increases its capacity to cleave substrates.

Supplemental Experimental Procedures

Features within the iRhom2 N-terminus

Putative 14-3-3 binding sites were determined using the online 14-3-3-Pred tool (Madeira et al., 2015) (see http://www.compbio.dundee.ac.uk/1433pred/run/job=s2948603276). Putative phosphorylated residues on iRhom2 from large scale phosphoproteomics studies were retrieved using the online Phosphosite tool (see http://www.phosphosite.org/proteinAction.do?id=10848&showAll-Sites=true for a detailed overview). Analysis of disordered regions in the iRhom2 N-terminus were performed with the prediction algorithm PONDR (Obradovic et al., 2005) (http://www.pondr.com/).

Reagents

The following reagents were used: 1,10-phenanthroline (Sigma), Batimastat (BB94, Calbiochem), Marimastat (MM, Calbiochem), GI254023X (GI, Sigma), PD184352 (Sigma), Dynasore (Calbiochem), SB202190 (Calbiochem), SP600125 (Calbiochem), Lipopolysaccharide, E. coli 055:B5 (sc-221855A, Santa Cruz), poly(I:C) (11C21-MM, InvivoGen), Phorbol 12-myristate 13-acetate (PMA, Sigma), Ionomycin (IO, Cayman), Gastrin Releasing Peptide (GRP, mouse, Phoenix Pharmaceuticals), Cycloheximide (sc-3508, Santa Cruz), 1-Step™ PNPP Substrate (PIER37621, Termo Fisher), TACE fluorogenic peptide (ANASPEC, 72085), Alkaline Phosphatase (MBIEF0651, Fermentas), Phos-tag Acrylamide (AAL-107, Wako Chemicals), Dithiobis (succinimidyl proprionate) (DSP, 10731945, Pierce), Sulfo-NHS-LC-Biotin (Thermo Scientific, 21335), Neutravidin agarose resin (Thermo Scientific, 29200), mouse TNF ELISA (558534, BD Biosciences), Concanavalin A Agarose (786-216, G-biosciences), cell viability stain 7AAD (420403, Biolegend).

Antibodies

The following antibodies were used: TACE (Ab39162, Abcam), alpha-Tubulin (sc-8035, Santa Cruz), phospho-ERK (9102, Cell Signalling Technology), total-ERK (9106, Cell Signalling Technology), p97 (Thermo, MA1-21412), HA-HRP (clone 3F10, Roche), V5-HRP (R961-25, Life Technologies). 14-3-3 (pan-specific, 8312, Cell Signalling Technology), ADAM10 (19026, Millipore), Transferrin receptor (13-6800, Life Technologies). Anti-iRhom2 polyclonal antibodies specific to the mouse iRhom2 N-terminus (amino acids 1-373) or raised against the iRhom homology domain, were previously described (Adrain et al., 2012). For immuno-precipitations, magnetic beads were used: anti-HA (15222405, Pierce), MagnaBind Goat Anti-Mouse IgG Beads (2135, 4, Thermo Scientific), MagnaBind Goat Anti-Rabbit IgG Beads (21356, Thermo Scientific). The following antibodies were used specifically for flow cytometry: Fc-block (clone 2.4G2, BD Biosciences), anti-HA (Ab9110, Abcam), Alexa 633-labeled secondary antibody (A-21070, Invitrogen), phycoerythrin conjugated anti-TACE (FAB9301P, R&D Systems), or isotype control IgG1-Phycoerythrin conjugated (IC062P, R&D Systems).

Cell culture

HEK293 ET, RAW264.7 and iRhom$1^{-/-}$ $2^{-/-}$ double KO (DKO) MEFs (previously described in Christova et al., 2013), untransformed and stably expressing iRhom2, were maintained under standard conditions.

BMDM were isolated from 12-16 weeks old WT and iRhom2$^{-/-}$ (iRhom2 KO) mice, and cultured as previously described (Adrain et al., 2012).

Retroviral transduction

HEK293 ET packaging cells (1 x 10^6) were transfected with pCL (-Eco, or 10A1) packaging plasmids (Naviaux et al., 1996) plus pM6P.BLAST empty vector (kind gift of F. Randow, Cambridge, UK) or pM6P containing mouse iRhom2 (WT or phospho-mutants) cDNA fused to a C-terminal HA tag. Cells transduced with the viral supernatant supplemented with polybrene 8 μg/mL, and selected with blasticidin (5 μg/mL) to generate stable cell lines. To transduce primary bone marrow-derived macrophages (BMDM) from iRhom2 KO mice, retroviruses were prepared as follows: HEK 293ET cells (24 x 10^6) were transfected with pMD-VSVG envelope plasmid, pMD-OGP helper plasmid (gift of Richard Mulligan), and empty vector pM6P.BLAST, or pM6P containing the iRhom2 (WT or non-phosphorylatable mutants) cDNA fused to a C-terminal HA tag. The viral supernatants were concentrated 300-fold using ultracentrifugation (90,000 g) at 4°C for 4h, followed by re-suspension in 0.1 % BSA in PBS. Cells were transduced with the concentrated virus, supplemented with 8 μg/ml polybrene. For the production of TACE and ADAM10 shRNA lentivirus, viral particles were concentrated 150-fold by ultracentrifugation of supernatants from HEK293 ET packaging cells transfected with pLVTHM-GFP plasmids encoding shRNAs specific to human ADAM10 or TACE (gift of Andreas Ludwig) plus pMD-VSVG and psPAX2.

Phos-tag gels and protein de-phosphorylation

All buffers used during sample preparation were devoid of EDTA (and 1,10-phenanthroline) to prevent chelation of the manganese ions required for phospho-protein binding to the Phos-tag acrylamide. Phosphate containing reagents were excluded to prevent competitive binding to the Phos-tag acrylamide. Cells were washed twice with ice-cold phos-tag wash buffer (10 mM Tris-HCl pH 7.4, 100 mM NaCl) before lysis in T-X100 lysis buffer supplemented with protease inhibitor cocktail, and phosphatase inhibitors (1.5 mM sodium orthovanadate, 10 mM sodium fluoride). Samples were run on 6 or 8 % acrylamide Tris-Glycine gels (prepared according to manufacturer instructions) supplemented with 5 μM Phos-tag acrylamide and 1 mM manganese chloride. Gels were run at 80 volts for approximately 5 hours (until the 75 KDa marker was at the bottom of the gel), and incubated in transfer buffer supplemented with 1 mM EDTA for 20 minutes to chelate manganese, followed by a second wash in transfer buffer. From this point onwards standard western blot protocols for transfer and antibody incubation were used. Where indicated, protein lysates (120 μg) were de-phosphorylated according to the manufacturer's instructions using 40 units of alkaline phosphatase and incubated at 37ºC for 1 hour prior to electrophoresis.

Flow Cytometry

After washing, the viability stain 7AAD was added and the samples were analyzed using a Cyan ADP analyzer (Beckman Coulter) and the FlowJo software, version 10.2. Cells were first gated based on size (SS vs FS) and then on singularity (Pulse Width vs FS). This was followed by 7AAD exclusion to gate for live cells (FL2 vs FL4). Live cells were gated on expression of HA or TACE (FL2 vs FL8). Unstained (Fc-blocked cells) were used as negative population. The staining index was calculated as: (fluorescence

intensity test population – fluorescence intensity negative population) / (2 x SD negative population) (Maecker et al., 2004).

TACE enzymatic assay on live cells

HEK293ET cells expressing empty vector, iRhom2-HA WT or iRhom2 Dead-HA were plated on 24 well plates ($1x10^6$ cells). The next day cells were washed in Optimem; for experiments involving PMA stimulation (Figure 7A,B) cells were starved for 6 hours in Optimem supplemented with 25 mM Hepes, the media was then changed to Optimem (25 mM Hepes) containing 0.75 µL of the fluorogenic TACE substrate and the drug treatments. Fluorescence was measured on a Victor 3 plate reader over 8 hours, at 37ºC, according to manufacturer instructions (ANASPEC SensoLyte 520 TACE Activity Assay Kit, 72085). TACE activity (Figure 5F, 7B), expressed as change in raw fluorescence units (RFU) per minute was calculated using linear regression fit of the raw fluorescence data. For all experimental conditions, TACE activity was background corrected for non-metalloprotease dependent cleavage of the peptide using BB94 treated samples. Time resolved fluorescence (AU, arbitrary units) shown in Figure 7A, is expressed as RFU for each experimental condition divided by the sum of the total RFU for each experiment; this step is a mathematical processing of raw data commonly used to minimize variability in absolute intensity values from experiment to experiment, and has been previously described for time-resolved luminescence from luciferase experiments (Bruning et al., 2012; Cavadas and Cheong, 2014).

Shedding assays

MEFs (3×10^5 in 6 well-plates) were transfected with 1 µg cDNA of TGF ✔-AP or BTC-AP using 3 µL Fugene. One day following transfection, cells were washed 3 times with serum free media before incubation for 1 hour in 1 mL Optimem (for basal shedding), followed by 1 hour with 1 mL Optimem containing 1 µM PMA or 2.5 µM ionomycin (for stimulated shedding), in the presence of metalloprotease inhibitors (BB94) and ADAM10 inhibitor (GI) where indicated. Supernatants from each incubation step were collected and incubated with the AP substrate p-nitrophenyl phosphate at room temperature. AP activity measured using a 96-well plate spectrophotometer (405 nm). Results are presented as PMA or IO "Stimulated shedding" calculated by dividing the PMA or IO-stimulated AP shedding by the constitutive shedding, as described previously (Sahin et al., 2006; Zheng et al., 2002).

Cloning of iRhom2-HA phospho-mutant constructs, R18-Dead mutant and TACE/ADAM10 domain swaps

The mouse iRhom2 Dead mutant fused to a C-terminal HA tag was inserted into empty vector pM6P.BLAST. This iRhom2 Dead DNA containing the 15 alanine mutated residues (Figure 4A) was prepared by gene synthesis (Gene Art, Life Technologies). To generate the 1-130, 131-212, 213-381, PCR was used to amplify the relevant fragments from the WT or iRhom2 Dead constructs, and Gibson cloning was used to assemble the final construct. Single serine to alanine phospho-mutants (Figure 4B,C,G,H) were generated by site-directed mutagenesis using KOD polymerase. To generate the R18-iRhom2 Dead mutant (Figure 5D-G), the R18 peptide sequence from the pSCM138 plasmid (Masters and Fu, 2001) was inserted into the N-terminus of the iRhom2 Dead mutant using Gibson cloning. TACE and ADAM10 V5 tagged

pcDNA6 constructs (Christova et al., 2013) were used to generate domain swap mutants of the ectodomain, transmembrane domain and cytoplasmic tails (Figure 7 D,E). DNA sequencing was used to confirm correct assembly of all constructs.

Co-immunoprecipitations

HEK293ET cells stably expressing empty vector, iRhom2-HA WT, Dead, phosphomutants, or the R18 mutant, were serum starved and stimulated with PMA (1 μM) for the indicated times (Figs. 5,7) before anti-HA immunoprecipitations. Alternatively, HEK293 ET cells were transfected with TACE/ADAM10 domain swap mutants and an empty vector or iRhom2-HA WT (Figure 7). Full details are provided in supplemental experimental procedures.Twenty-four hours after transfection cells were lysed for 10 minutes on ice in TX-100 lysis buffer (1% Triton X-100, 150 mM NaCl, 50 mM Tris-HCl, pH 7.4) containing complete protease inhibitor cocktail (Roche), 10 mM 1,10-phenanthroline (to inhibit TACE autoproteolysis) and phosphatase inhibitors 10 mM sodium fluoride and 1.5 mM sodium orthovanadate. Post-nuclear supernatants were pre-cleared with unconjugated magnetic beads for 90 minutes with rotation, followed by overnight incubation with anti-HA magnetic beads. Beads were washed for 10 minutes, 4 times, at 4ºC with Triton X-100 lysis buffer (with 1,10-phenanthroline and phosphatase inhibitors) supplemented with NaCl to 300 mM. Samples were eluted with 1.5 X sample buffer and incubated at 65ºC for 15 minutes before loading.

Mass spectrometry

Cells were washed twice with ice-cold PBS, and lysed for 10 minutes on ice in TX-100 lysis buffer containing complete protease inhibitor cocktail and phosphatase inhibitors. Post-nuclear supernatants were pre-cleared with unconjugated magnetic beads for 90 minutes with rotation, followed by 90 minutes incubation with anti-HA magnetic beads at 4ºC. Beads were washed for 10 minutes, 4 times, at 4ºC with 150 mM NaCl, 50 mM Tris-HCl, pH 7.4, supplemented with phosphatase inhibitors. For the data in Table 1, Table S1, Figure 4, Figure 5, Figure S1 and Figure S3, collected in Edinburgh, the following protocol was used. Immunoprecipitated samples were subjected to an on-beads digestion, peptide purification and mass spectrometry analysis as previously described (Turriziani et al., 2014). Briefly, the proteins bound to the beads were digested with mass spectrometry-grade porcine trypsin (Promega) overnight at room temperature. The proteolytic peptides were reduced and alkylated with DTT and Iodoacetamide, desalted and analysed on a Q-Exactive+ mass spectrometer coupled to a nano uHPLC (both Thermo Fisher Scientific). Analysis of phosphorylation sites were performed with MaxQuant software version 1.5.5.1, using human and mouse databases, Carbamylation as fixed and phospho (STY), N-terminal acetylation (protein) and oxidation (M) as variable modifications. The abundance of the different phosphopeptides was determined as the ratio obtained by dividing the intensity of the phosphopeptides by the intensity of the corresponding protein in each sample. N=2 biological replicates each. Data presented in Figure S3B, was collected from the mass-spec data from Edinburgh, and an additional set of experiments (2 biological replicates) performed in Vienna in iRhom2 immunoprecipitates, prepared as described above, with the addition of a cell permeable crosslinker (DSP).

For the data collected in Vienna (Figure S3B), a slightly different protocol was used, in brief, immunoprecipitates were enzymatically digested on 3 kD MWCO filters (Pall Austria Filter GmbH) using

an adaption of the FASP protocol as described previously (Bileck et al., 2014; Slany et al., 2016). After pre-concentration of the samples, protein reduction and alkylation was performed, then trypsin was added and incubated at 37°C for 18h. The digested peptide samples were dried and stored at -20°C until further LC-MS/MS analyses. Reconstitution of dried peptide samples was achieved by adding 5 µl 30% formic acid (FA) containing 10 fmol each of 4 synthetic standard peptides and further dilution with 40 µl mobile phase A (98% H_2O, 2% ACN, 0.1% FA), as described previously (Bileck et al., 2014; Wiśniewski et al., 2009). LC-MS/MS analyses were performed using a Dionex Ultimate 3000 nano LC-system coupled to a QExactive orbitrap mass spectrometer equipped with a nanospray ion source (Thermo Fisher Scientific). For LC-MS/MS analysis, 5µl of the peptide solution were loaded and pre-concentrated on a 2cm x75µm C18 Pepmap100 pre-column (Thermo Fisher Scientific) at a flow rate of 10 µl/min using mobile phase A. Following this pre-concentration, peptides were eluted from the pre-column to a 50cm x75µm Pepmap100 analytical column (Thermo Fisher Scientific) at a flow rate of 300nl/min and further separation was achieved using a gradient from 7% to 40% mobile phase B (80% ACN, 20% H_2O, 0.1% FA) over 85min including column washing and equilibration steps. For mass spectrometric analyses, MS scans were accomplished in the range from m/z 400-1400 at a resolution of 70000 (at m/z =200). Subsequently, data-dependent MS/MS scans of the 8 most abundant ions were performed using HCD fragmentation at 30% normalized collision energy and analyzed in the orbitrap at a resolution of 17500 (at m/z =200). Protein identification was achieved using Proteome Discoverer 1.4 (Thermo Fisher Scientific, Austria) running Mascot 2.5 (Matrix Science). Therefore, raw data were searched against the human proteome in the SwissProt Database (version 11/2015 with 20.193 entries) with a mass tolerance of 50 ppm at the MS1 level and 100 mmu at the MS2 level, allowing for up to two missed cleavages per peptide. Further search criteria included carbamidomethylation as fixed peptide modification and methionine oxidation as well as protein N-terminal acetylation as variable modifications.

Cell Surface Biotinylation

Biotinylation was performed as previously described for BMDM (Adrain et al., 2012) with small modifications, as detailed in supplemental experimental procedures. RAW264.7 macrophages ($1.5x10^6$ cells, 6 well plates) were moved to a cold room (at 4ºC), washed with ice-cold PBS pH 8.0 for 10 minutes, incubated with (1 mg/mL) Sulfo-NHS-LC-Biotin in PBS pH 8.0, according to the manufacturer's instructions. Dynasore (40 µM) was used during the biotinylation, to minimize internalization of the unreacted Biotin and biotinylated proteins. Following quenching with 50 mM Tris in PBS, cells were lysed for 10 minutes with TX-100 lysis buffer (1,10-phenathroline, protease inhibitors, 50 mM Tris), then biotin labelled surface proteins from post-nuclear supernatants were captured on neutravidin agarose resin at 4°C overnight. The resin was washed 3 times, 10 minutes, with TX-100 lysis buffer containing 450 mM NaCl at 4ºC. Samples were eluted with 1.5X sample buffer and incubated at 65ºC for 15 minutes, before loading.

Glycoprotein enrichment using Concanavalin A

To improve the detection of TACE, MEFs were lysed in TX-100 lysis buffer supplemented with 1 mM EDTA, 1 mM $MnCl_2$, 1 mM $CaCl_2$ and glycoproteins were captured using Concanavalin A (ConA) Agarose. Beads were washed twice in the same buffer and eluted by heating for 15 min at 65ºC in sample buffer

supplemented with 15% sucrose.

TNF ELISA in iRhom2 KO bone marrow-derived macrophages expressing iRhom2-HA

On day 4 post-isolation, iRhom2 KO BMDM were plated (1.2 x 10^5 cells, 12 well plate). The following day, cells were transduced with 10 μL of 300X concentrated retrovirus mixed with polybrene 8 μg/mL. Culture media were changed every second day and cells were stimulated at day 10 with 1μg/mL LPS for 3 hours. The supernatant was collected to measure TNF release using ELISA according to the manufacturer's instructions.

Table S1. iRhom2 (mouse) phosphorylation sites identified in large proteomic screens and in the current study

	S60	S83	S85	S87	S99	S147	Y199	T258	S293	S295	S298	S310	S357	S359	S360	T361
Proteomics screens	+	+	+	+	+	+	+	+	+	+	+	-	+	+	+	+
This study	+	+	-	-	-	+	-	-	-	-	+	+	-	+	-	-

Table S2. Phosphorylation-dependent changes in the iRhom2 interactome

iRhom2 interactors with altered binding upon PMA stimulation					
	# proteins	%			
WT PMA > WT	9	3,3			
WT PMA < WT	2	0,7			
WT PMA = WT	262	95,6			
iRhom2 interactors with altered binding in the WT compared to the Dead mutant					
DMSO			PMA		
	# proteins	%		# proteins	%
WT > EV	274	100	WT > EV	272	100
Dead < WT	15	5,5	Dead < WT	13	4,8
Dead > WT	4	1,5	Dead > WT	5	1,8
Dead = WT	255	93,1	Dead = WT	254	93,4

Supplementary References

Adrain, C., Zettl, M., Christova, Y., Taylor, N., and Freeman, M. (2012) Tumor Necrosis Factor Signaling Requires iRhom2 to Promote Trafficking and Activation of TACE. Science, *335*, 225–228.

Bileck, A., Kreutz, D., Muqaku, B., Slany, A., and Gerner, C. (2014) Comprehensive assessment of proteins regulated by dexamethasone reveals novel effects in primary human peripheral blood mononuclear cells. J Proteome Res, *13*, 5989–6000.

Bruning, U., Fitzpatrick, S.F., Frank, T., Birtwistle, M., Taylor, C.T., and Cheong, A. (2012) NFκB and HIF display synergistic behaviour during hypoxic inflammation. Cell. Mol. Life Sci., *69*, 1319–1329.

Cavadas, M.A.S., and Cheong, A. (2014) Monitoring of Transcriptional Dynamics of HIF and NFκB Activities. C.E. Badr, ed. (Totowa, NJ: Humana Press), pp. 97–105.

Christova, Y., Adrain, C., Bambrough, P., Ibrahim, A., and Freeman, M. (2013) Mammalian iRhoms have distinct physiological functions including an essential role in TACE regulation. EMBO Rep,

Madeira, F., Tinti, M., Murugesan, G., Berrett, E., Stafford, M., Toth, R., Cole, C., MacKintosh, C., and Barton, G.J. (2015) 14-3-3-Pred: improved methods to predict 14-3-3-binding phosphopeptides. Bioinformatics, *31*, 2276–2283.

Maecker, H.T., Frey, T., Nomura, L.E., and Trotter, J. (2004) Selecting fluorochrome conjugates for maximum sensitivity. Cytometry, *62A*, 169–173.

Masters, S.C., and Fu, H. (2001) 14-3-3 proteins mediate an essential anti-apoptotic signal. J Biol Chem, *276*, 45193–45200.

Naviaux, R.K., Costanzi, E., Haas, M., and Verma, I.M. (1996) The pCL vector system: rapid production of helper-free, high-titer, recombinant retroviruses. J Virol, *70*, 5701–5705.

Obradovic, Z., Peng, K., Vucetic, S., Radivojac, P., and Dunker, A.K. (2005) Exploiting heterogeneous sequence properties improves prediction of protein disorder. Proteins, *61 Suppl 7*, 176–182.

Sahin, U., Weskamp, G., Zheng, Y., Chesneau, V., Horiuchi, K., and Blobel, C.P. (2006) A sensitive method to monitor ectodomain shedding of ligands of the epidermal growth factor receptor. Methods Mol Biol, *327*, 99–113.

Slany, A., Bileck, A., Kreutz, D., Mayer, R.L., Muqaku, B., and Gerner, C. (2016) Contribution of Human Fibroblasts and Endothelial Cells to the Hallmarks of Inflammation as Determined by Proteome Profiling. Mol Cell Proteomics, *15*, 1982–1997.

Wiśniewski, J.R., Zougman, A., Nagaraj, N., and Mann, M. (2009) Universal sample preparation method for proteome analysis. Nat Methods, *6*, 359–362.

Zheng, Y., Schlondorff, J., and Blobel, C.P. (2002) Evidence for regulation of the tumor necrosis factor alpha-convertase (TACE) by protein-tyrosine phosphatase PTPH1. J Biol Chem, *277*, 42463–42470.
