# Supplementary material for: Phosphorylation of iRhom2 Controls Stimulated Proteolytic Shedding by the Metalloprotease ADAM17/TACE
Source: Cell Rep. 2017 Oct 17;21(3):745–57. doi: 10.1016/j.celrep.2017.09.074 (PMC5656746; doi:10.1016/j.celrep.2017.09.074)
Supplement: Document S2. Article plus Supplemental Information [file mmc2.pdf]

Cell Reports

Article

Phosphorylation of iRhom2 Controls Stimulated Proteolytic Shedding by the Metalloprotease ADAM17/TACE

Graphical Abstract

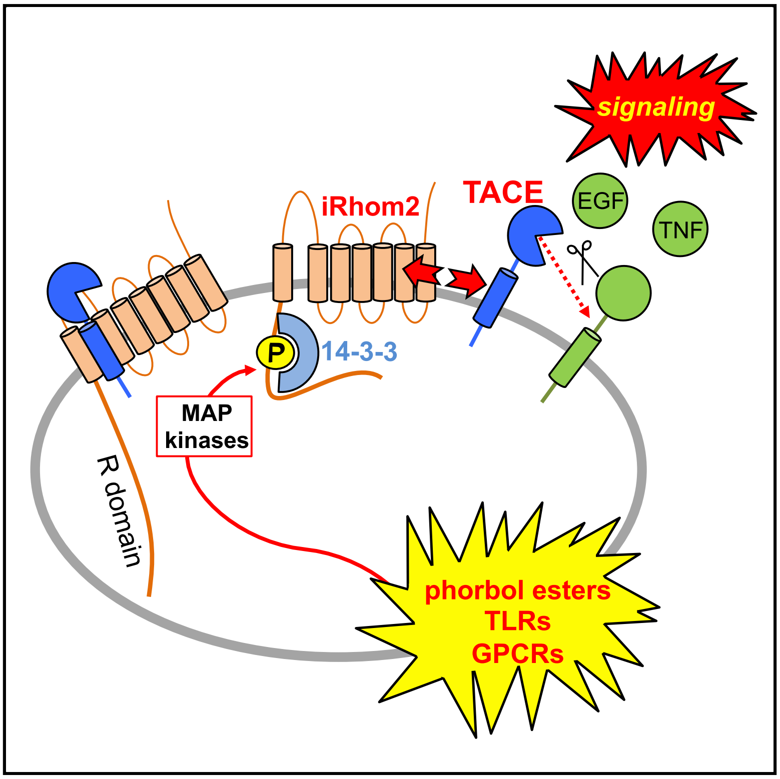

Highlights

- iRhom2 is phosphorylated in response to stimuli that activate the sheddase TACE
- Blocking iRhom phosphorylation represses TACE stimulated shedding
- Phosphorylated iRhom2 recruits 14-3-3 and dissociates from TACE, enabling shedding
- iRhom2 is thus a signal integrator and transducer of stimulated TACE shedding

Authors

Miguel Cavadas, Ioanna Oikonomidi, Catarina J. Gaspar, ..., Pedro M. Domingos, Alex von Kriegsheim, Colin Adrain

Correspondence

cadrain@igc.gulbenkian.pt

In Brief

Cavadas et al. examine how the metalloprotease TACE is stimulated to shed its substrates, observing that iRhom2, a molecule essential for TACE trafficking, is phosphorylated in response to stimulants (PMA, TLRs, and GPCRs). iRhom phosphorylation requires MAPKs and recruits 14-3-3, which causes iRhom2/TACE dissociation, enabling TACE to cleave its substrates.

Cavadas et al., 2017, Cell Reports *21*, 745–757
October 17, 2017 © 2017 The Authors.
https://doi.org/10.1016/j.celrep.2017.09.074

CrossMark
CellPress

OPEN ACCESS
CellPress

Phosphorylation of iRhom2 Controls Stimulated Proteolytic Shedding by the Metalloprotease ADAM17/TACE

Miguel Cavadas,[1] Ioanna Oikonomidi,[1] Catarina J. Gaspar,[1,2] Emma Burbridge,[1] Marina Badenes,[1] Inês Félix,[1] Alfonso Bolado,[3] Tianyi Hu,[1] Andrea Bileck,[4,5] Christopher Gerner,[4] Pedro M. Domingos,[2] Alex von Kriegsheim,[3] and Colin Adrain[1,6,*]
[1]Membrane Traffic Lab, Instituto Gulbenkian de Ciência (IGC), Oeiras, Portugal
[2]Instituto de Tecnologia Química e Biológica (ITQB-NOVA), Oeiras, Portugal
[3]Edinburgh Cancer Research UK Centre, Institute of Genetics and Molecular Medicine, University of Edinburgh, Edinburgh, UK
[4]Institut für Analytische Chemie, Universität Wien, Währinger Strasse 38, 1090 Vienna, Austria
[5]Present address: Department of Clinical Research, Department of Nephrology and Hypertension, Bern University Hospital, University of Bern, Bern, Switzerland
[6]Lead Contact
*Correspondence: cadrain@igc.gulbenkian.pt
https://doi.org/10.1016/j.celrep.2017.09.074

SUMMARY

Cell surface metalloproteases coordinate signaling during development, tissue homeostasis, and disease. TACE (TNF-α-converting enzyme), is responsible for cleavage ("shedding") of membrane-tethered signaling molecules, including the cytokine TNF, and activating ligands of the EGFR. The trafficking of TACE within the secretory pathway requires its binding to iRhom2, which mediates the exit of TACE from the endoplasmic reticulum. An important, but mechanistically unclear, feature of TACE biology is its ability to be stimulated rapidly on the cell surface by numerous inflammatory and growth-promoting agents. Here, we report a role for iRhom2 in TACE stimulation on the cell surface. TACE shedding stimuli trigger MAP kinase-dependent phosphorylation of iRhom2 N-terminal cytoplasmic tail. This recruits 14-3-3 proteins, enforcing the dissociation of TACE from complexes with iRhom2, promoting the cleavage of TACE substrates. Our data reveal that iRhom2 controls multiple aspects of TACE biology, including stimulated shedding on the cell surface.

INTRODUCTION

A major mechanism of cellular communication involves "shedding": the stimulated proteolytic release of signaling molecules from the plasma membrane. The TNF-α-converting enzyme (TACE), also called ADAM17 (a disintegrin and metalloprotease-17), is a prominent sheddase with more than 80 cellular substrates (Gooz, 2010). Mutant mouse studies emphasize the essential role of TACE in inflammation: it sheds the inflammatory cytokine, tumor necrosis factor (TNF) (Horiuchi et al., 2007). TACE also plays an essential physiological role in growth factor signaling, by shedding multiple activating ligands of the epidermal growth factor receptor (EGFR), a receptor important for epithelial development, homeostasis, and cancer (Peschon et al., 1998).

TACE is synthesized as a catalytically inactive precursor in the endoplasmic reticulum (ER). To be proteolytically active, TACE must undergo a maturation step, which occurs in the *trans*-Golgi network, where pro-protein convertases cleave off TACE's inhibitory N-terminal prodomain, rendering it basally active (Schlöndorff et al., 2000). The work of several groups, including ours, recently identified polytopic membrane proteins called iRhoms as essential regulators of TACE maturation (Adrain et al., 2012; Siggs et al., 2012; Christova et al., 2013). In iRhom null cells, TACE is retained in the ER, fails to undergo prodomain removal, and is consequently proteolytically inactive.

As overexpressed iRhoms are predominantly ER localized (Zettl et al., 2011), the current working hypothesis is that iRhoms mediate the ER-to-Golgi trafficking of TACE (Adrain and Freeman, 2012). However, several observations are incongruent with this model: first, cross-linking experiments show that iRhom binds efficiently to mature TACE, indicating that the molecules still interact following prodomain removal in the *trans*-Golgi network (Adrain et al., 2012). Second, endogenous iRhom2 contains endoglycosidase H-insensitive glycans, indicating that it traffics beyond the ER (Adrain et al., 2012). Moreover, overexpressed iRhoms localize to the plasma membrane (Maney et al., 2015).

The cell surface sheddase activity of TACE is subject to another important layer of regulation: stimulation by various signaling pathways. TACE stimulation is involved in inflammation, tissue damage, and cancer; stimulatory agents include the phorbol ester phorbol 12-myristate 13-acetate (PMA) (Arribas et al., 1996), cytokine receptors (Hall and Blobel, 2012),Toll-like receptors (Brandl et al., 2010), and G protein-coupled receptors (Prenzel et al., 1999; Wetzker and Böhmer, 2003). Many stimuli that activate TACE converge on the cytoplasm, activating kinases, including members of the MAP (mitogen-activated protein kinase) kinase family, that control

CrossMark

This is an open access article under the CC BY license (http://creativecommons.org/licenses/by/4.0/).

OPEN
ACCESS
CellPress

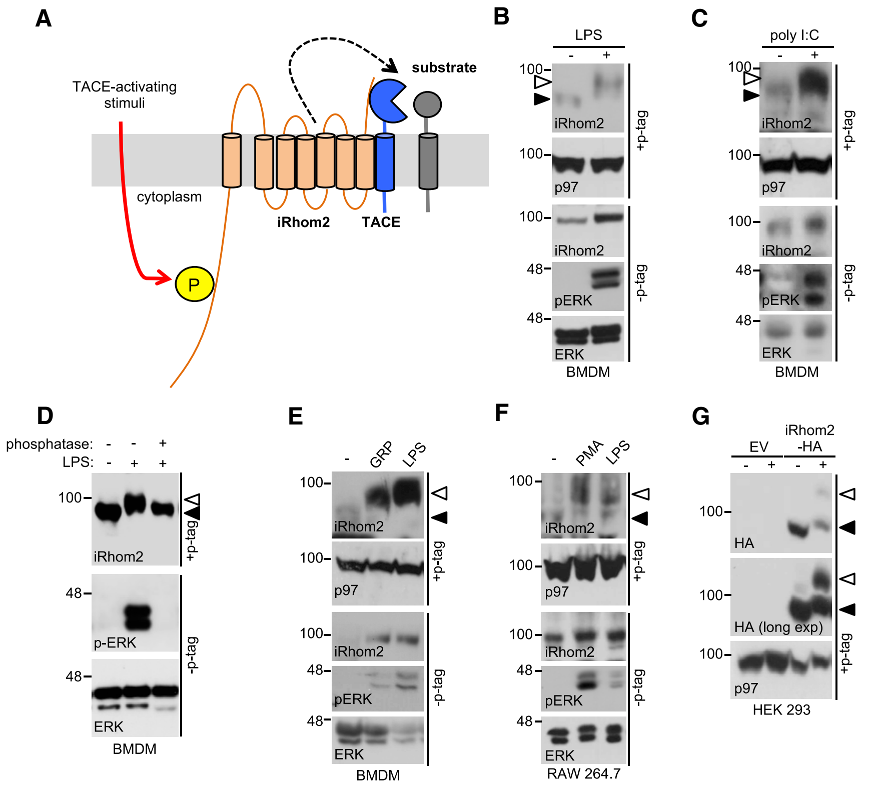

Figure 1. iRhom2 Is Phosphorylated in Response to TACE-Activating Stimuli

(A) Schematic envisaging iRhom2 phosphorylation as a signal integrator for TACE-activating stimuli.

(B and C) Phos-tag gels show that endogenous iRhom2 is phosphorylated in bone marrow-derived macrophages (BMDMs) stimulated with LPS (B) and (C) poly(I:C).

(D) The mobility shift in iRhom2 is reverted by phosphatase treatment.

(E) Endogenous iRhom2 is phosphorylated in BMDMs stimulated with the G protein-coupled receptor ligand gastrin-releasing peptide (GRP).

(F) Endogenous iRhom2 is phosphorylated in response to PMA and LPS in RAW264.7 macrophages.

(G) HA-tagged mouse iRhom2, stably expressed in HEK293ET cells, is phosphorylated in response to PMA.

Throughout, a white arrowhead denotes phosphorylated iRhom2 and a black arrowhead non-phosphorylated iRhom2. p97 immunoblots are a loading control and pERK (phosphorylated ERK) a positive control for stimulation. Here and throughout, cells were stimulated for 15 min with LPS (1 μg/mL), GRP (200 ng/mL), and PMA (1 μM) and for 60 min with poly(I:C) (1 μg/mL). See also Figure S1.

release of several endogenous TACE substrates, including TNF and EGFR ligands (Díaz-Rodríguez et al., 2002; Rousseau et al., 2008; Xu and Derynck, 2010; Scott et al., 2011; Sommer et al., 2016). There is an emerging consensus that TACE-activating stimuli enforce rearrangements in the ectodomain of TACE, resulting in enhanced TACE substrate cleavage (Le Gall et al., 2010). This process is negatively regulated by cell surface protein disulfide isomerases, and positively regulated by the externalization of phosphatidylserine (PS) to the outer leaflet of the plasma membrane (Düsterhöft et al., 2013; Sommer et al., 2016).

A complication in envisaging how stimuli are transduced to TACE is the conflicting evidence for the importance of the TACE cytoplasmic tail. The cytoplasmic tail of TACE is phosphorylated in response to shedding stimulants, but there is no consensus concerning the impact of these phosphorylation events (Fan and Derynck, 1999; Díaz-Rodríguez et al., 2002; Fan et al., 2003; Soond et al., 2005; Xu and Derynck, 2010; Scott et al., 2011; Xu et al., 2012). Indeed, several studies have shown that the TACE cytoplasmic tail is not required for TACE stimulation (Le Gall et al., 2010; Hall and Blobel, 2012), suggesting that another transmembrane protein containing a cytoplasmic tail transduces cytoplasmic stimulatory signals to the extracellular protease domain of TACE.

iRhom2 has a long N-terminal cytoplasmic tail that is predicted to be intrinsically disordered, decorated with signatures of a signaling hub, including predicted 14-3-3 binding sites, predicted ERK (extracellular signal-regulated kinase) kinase docking sites (Roux and Blenis, 2004) (Figure S1A), and multiple potentially phosphorylated residues (Figure 1A; Table S1). Recently, Maney et al. (2015) showed that TACE activity was enhanced in cells expressing mutants of iRhom truncated within their cytoplasmic tails, but the mechanistic basis of the phenomenon was unexplored. Additional evidence for a regulatory role of the iRhom2 N terminus comes from the identification of gain of function mutations in the N terminus of human iRhom2 that enhance TACE sheddase activity (Maney et al., 2015) and cause tylosis with esophageal cancer, a keratinocyte hyperproliferative condition (Blaydon et al., 2012).

Here, we identify that phosphorylation of the iRhom2 cytoplasmic tail plays a central and essential role in the regulation of TACE stimulation.

RESULTS

iRhom2 Is Phosphorylated in Response to Physiological TACE-Activating Stimuli

To test the hypothesis that iRhom2 is an essential co-factor for TACE stimulation (Figures 1A and S1A), we analyzed iRhom2 phosphorylation in response to a panel of physiological TACE-activating stimuli. To detect phosphorylation of iRhom2, we supplemented SDS-PAGE gels with Phos-tag, a compound that slows the electrophoretic mobility of phosphorylated proteins. As shown in Figures 1B and 1C, treatment of cells with the Toll-like receptor ligands lipopolysaccharide (LPS) and poly(I:C), stimuli that provoke the shedding of TNF, induced phosphorylation of endogenous iRhom2 in primary murine bone marrow-derived macrophages. Phosphatase treatment of lysates from

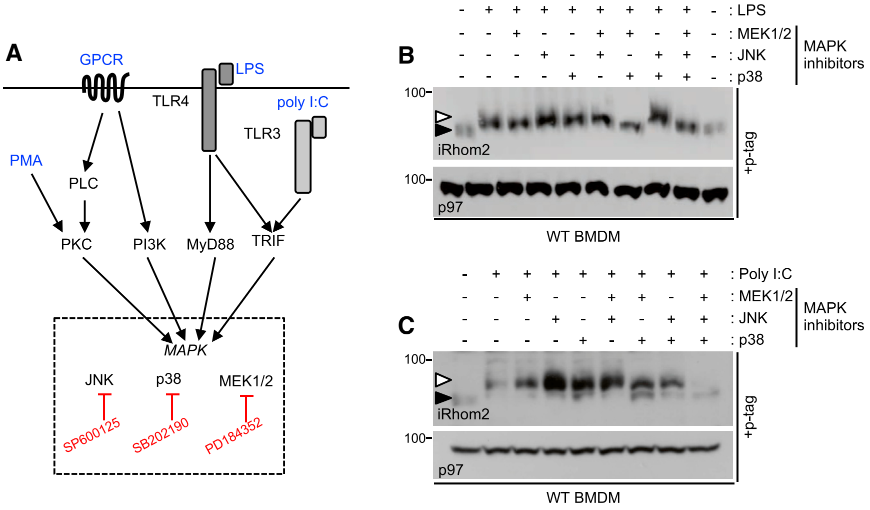

Figure 2. iRhom2 Phosphorylation Depends on MAP Kinases Downstream of Toll-like Receptor Signaling

(A) Schematics illustrating how signaling downstream of TLR3/4, GPCRs, and PMA converges on MAPK activation. Inhibitors are highlighted in red. Adapted from Goldsmith and Dhanasekaran (2007); Steinberg (2008), and Oda and Kitano (2006).

(B) A combination of ERK- and p38-MAPK inhibitors prevent endogenous iRhom2 phosphorylation in LPS-stimulated BMDMs.

(C) MAPK inhibitors (MEK1/2, JNK, and p38) prevent endogenous iRhom2 phosphorylation in poly(I:C)-stimulated BMDM.

Throughout, cells were pre-treated for 30 min with inhibitors (2.5 μM PD184352 [MEK1/2 inhibitor], 5 μM SP600125 [JNK inhibitor], 1 μM SB 202190 [p38 inhibitor]) followed by LPS (15 min) or poly(I:C) (60 min) treatment.

LPS-stimulated macrophages confirmed that the slower migration of iRhom2 was indeed caused by phosphorylation (Figure 1D). We next looked at activation of a G protein-coupled receptor, a pathway linked to EGFR transactivation (Wetzker and Böhmer, 2003). We found that the gastrin-releasing peptide receptor, which is implicated in EGFR-associated cancers, also triggered iRhom2 phosphorylation (Figure 1E). The phorbol ester PMA, a stimulus commonly used to trigger TACE-mediated substrate shedding, also induced endogenous iRhom2 phosphorylation in RAW264.7 macrophages (Figure 1F) and HEK293ET cells stably expressing HA-tagged mouse iRhom2 (Figure 1G). In summary, iRhom2 is phosphorylated in response to range of physiological stimuli known to rapidly induce TACE sheddase activity.

iRhom2 Phosphorylation Is Dependent on MAP Kinases

We next addressed which kinases were potentially involved in iRhom2 phosphorylation. The major part of iRhom2 that is exposed to the cytoplasm, accessible to kinases, is its extended N-terminal cytoplasmic tail (Figure 1A). As MAP kinases can be activated by the TACE stimuli (Díaz-Rodríguez et al., 2002; Yamamoto et al., 2002; Scott et al., 2011; Czepielewski et al., 2012) that we here show trigger iRhom2 phosphorylation (Figure 1), we focused on this kinase family. LPS activation of Toll-like receptor-4 converges on MAP kinases (Figure 2A). To examine the potential role of these kinases in iRhom2 phosphorylation, we used a pool of inhibitors targeting MEK1/2, whose downstream kinase targets are ERK1/2, plus inhibitors for JNK or p38 (Figures 2A and 2B). Although no single inhibitor blocked phosphorylation, a combination of p38 and MEK1/2 inhibitors abolished LPS-induced phosphorylation of endogenous iRhom2 (Figure 2B). In contrast, inhibitors of all three MAPK were required to block iRhom2 phosphorylation in response to Toll-like receptor-3 activation by poly(I:C) (Figure 2C). We conclude that MAP kinases are required for phosphorylation of endogenous iRhom2 in response to Toll-like receptor activation, stimuli that promote TNF shedding.

Rapid Stimulation of TACE Shedding Requires Phosphorylation of iRhom2

In contrast to the slow rate of TACE trafficking from the ER to the Golgi (Schlöndorff et al., 2000), TACE stimulation occurs with rapid kinetics, potentially acting mechanistically to control rearrangements in the extracellular domains of TACE or the access of TACE's active site to substrates (Le Gall et al., 2010). We next investigated the impact of iRhom2 phosphorylation on its ability to regulate TACE activity. Notably, several high throughput phosphoproteomic studies detected phosphorylated residues (Table S1) within the iRhom2 cytoplasmic tail, triggered in response to LPS and to G protein-coupled receptor agonists known to stimulate TACE activity (Christensen et al., 2010; Weintz et al., 2010). As these phosphorylation events were not confirmed biochemically, nor was their functional relevance explored, we examined this in depth.

iRhom1/iRhom2 double-knockout (DKO) mouse embryonic fibroblasts (MEFs) are devoid of TACE activity and cannot support stimulated TACE shedding (Christova et al., 2013). To examine the functional impact of blocking iRhom2 phosphorylation on the regulation of TACE, we constructed a compound mutant in which all 15 putative phosphorylation sites identified by the phosphoproteomic screens (Table S1) were mutated to alanine to block the ability of the protein to be phosphorylated. This phosphorylation-dead mutant (hereafter referred to as iRhom2 Dead for simplicity) could not be phosphorylated in response to PMA, indicating that the relevant sites were among the engineered mutations (Figure 3A).

To examine the ability of the iRhom2 Dead mutant to rescue TACE stimulation, we assessed its capacity to support shedding of the EGFR ligand transforming growth factor alpha fused to alkaline phosphatase (TGF-α-AP) into culture medium (Sahin et al., 2004). A time-course analysis of PMA-stimulated TGF-α-AP shedding revealed that, as expected (Le Gall et al., 2010), induction of TACE shedding occurred rapidly, within 5–20 min (Figure 3B). Notably, although TGF-α-AP shedding was rescued by expression of wild-type (WT) iRhom2, the Dead mutant was significantly impaired in its ability to rescue

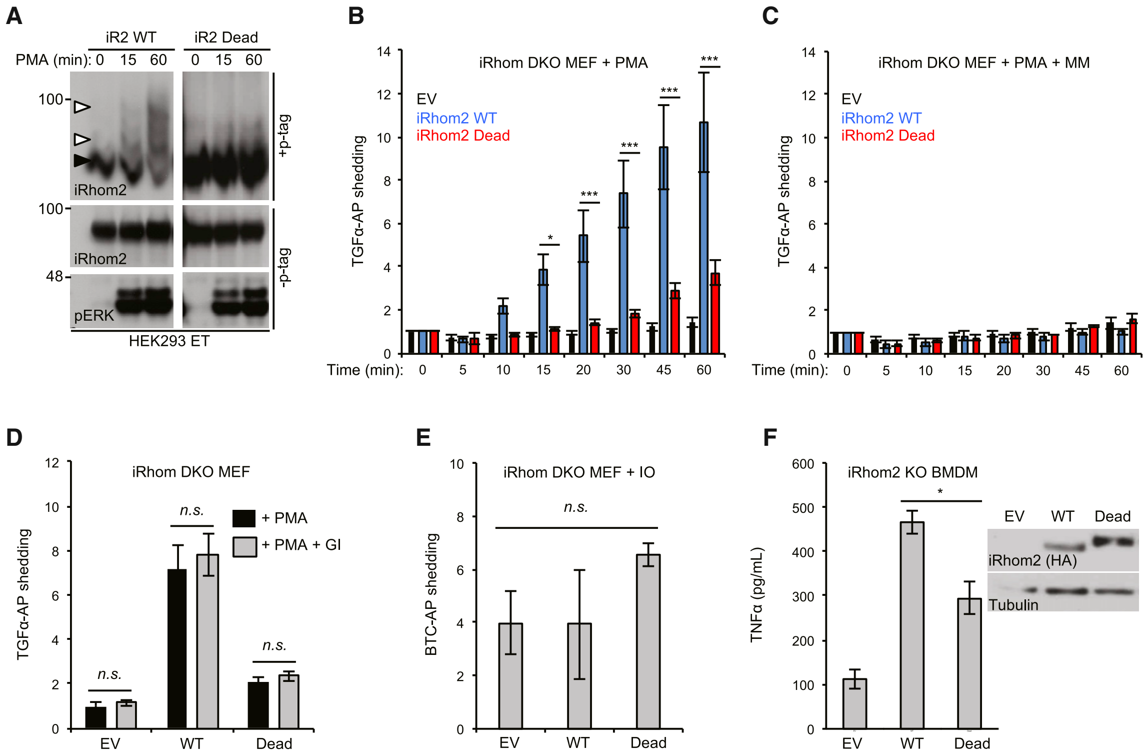

Figure 3. iRhom2 Cytoplasmic Tail Phosphorylation Is Required for Rapid Induction of TACE-Dependent Shedding

(A) iRhom2 Dead mutant stably expressed in HEK293ET cells is not phosphorylated in response to PMA.

(B) PMA-stimulated TGF-α-AP shedding requires iRhom2 phosphorylation. iRhom1/2 DKO MEFs were transduced with iRhom2 WT, iRhom2 Dead mutant, or empty vector (EV) retrovirus.

(C) Marimastat (MM; 5 μM) was used to inhibit shedding by metalloproteases.

(D) The ADAM10-specific inhibitor GI254023X (GI; 1 μM, 60 min) did not affect TGF-α-AP shedding in rescue assays in iRhom DKO MEFs expressing WT iRhom2 versus the Dead mutant or empty vector.

(E) Ionomycin (IO; 2.5 μM, 60 min) stimulated shedding of betacellulin-AP (BTC-AP) was unaffected by iRhom2 phosphorylation.

(F) iRhom2 KO BMDMs transduced with iRhom2 Dead retrovirus are defective in the shedding of TNF after 3 hr of LPS stimulation.

Data are presented as mean ± SEM. See also Table S1.

shedding in iRhom DKO MEFs, at all time points (Figure 3B). At the later time points, chronic exposure of cells to PMA may activate iRhom-independent shedding pathways, explaining the incomplete loss of shedding in the Dead mutant. Importantly, the effect observed was specific to TACE: TGF-α-AP shedding was blocked by the metalloprotease inhibitor Marimastat (MM) (Figure 3C) but not by the ADAM10-selective inhibitor GI254023X (Ludwig et al., 2005) (Figure 3D). Moreover, ionomycin (IO)-triggered shedding of alkaline-phosphatase fused to betacellulin (BTC), a substrate of ADAM10, the metalloprotease closely related to TACE, was unaffected by the iRhom2 Dead mutant (Figure 3E). In summary, our results reveal that iRhom2 phosphorylation is essential for the rapid activation of TACE.

We validated our findings using an alternative and physiologically relevant paradigm of TACE activation. In contrast to models of TACE shedding that rely on rapid cleavage of pre-existing substrate, in macrophages, the shedding of TNF is biologically distinct: it first requires TNF transcription and translation, in response to Toll-like receptor activation. As iRhom2 is necessary and sufficient for TACE maturation in macrophages (Adrain et al., 2012), we used retrovirus to transduce iRhom2 WT, or the Dead mutant, into primary macrophages of iRhom2 knockout (KO) mice. Similar to the MEF experiments, expression of the iRhom2 Dead mutant was less efficient in restoring LPS-triggered TNF shedding than WT iRhom2 (Figure 3F). In summary, phosphorylation of iRhom2 is crucial for the rapid stimulation of TACE activity by phorbol esters and for the more delayed release of endogenous TNF following Toll-like receptor stimulation.

iRhom2 Phosphorylation at Ser 83 Recruits 14-3-3 Proteins, Controlling TACE Stimulation

Proteins are often phosphorylated at multiple positions, sometimes by several kinases (Cohen, 2000). To learn more about which residues within iRhom2 promote TACE sheddase activity, we deconvoluted the iRhom2 Dead compound mutant into three separate mutant clusters: 1–130, 131–212, and 213–381, on an otherwise WT, full-length iRhom2 background (Figure 4A). In TGF-α-AP-shedding assays in DKO MEFs, expression of the 1–130 mutant, which contains the N-terminal five potential phosphorylation sites, still failed to restore TACE activity, indicating

OPEN ACCESS
CellPress

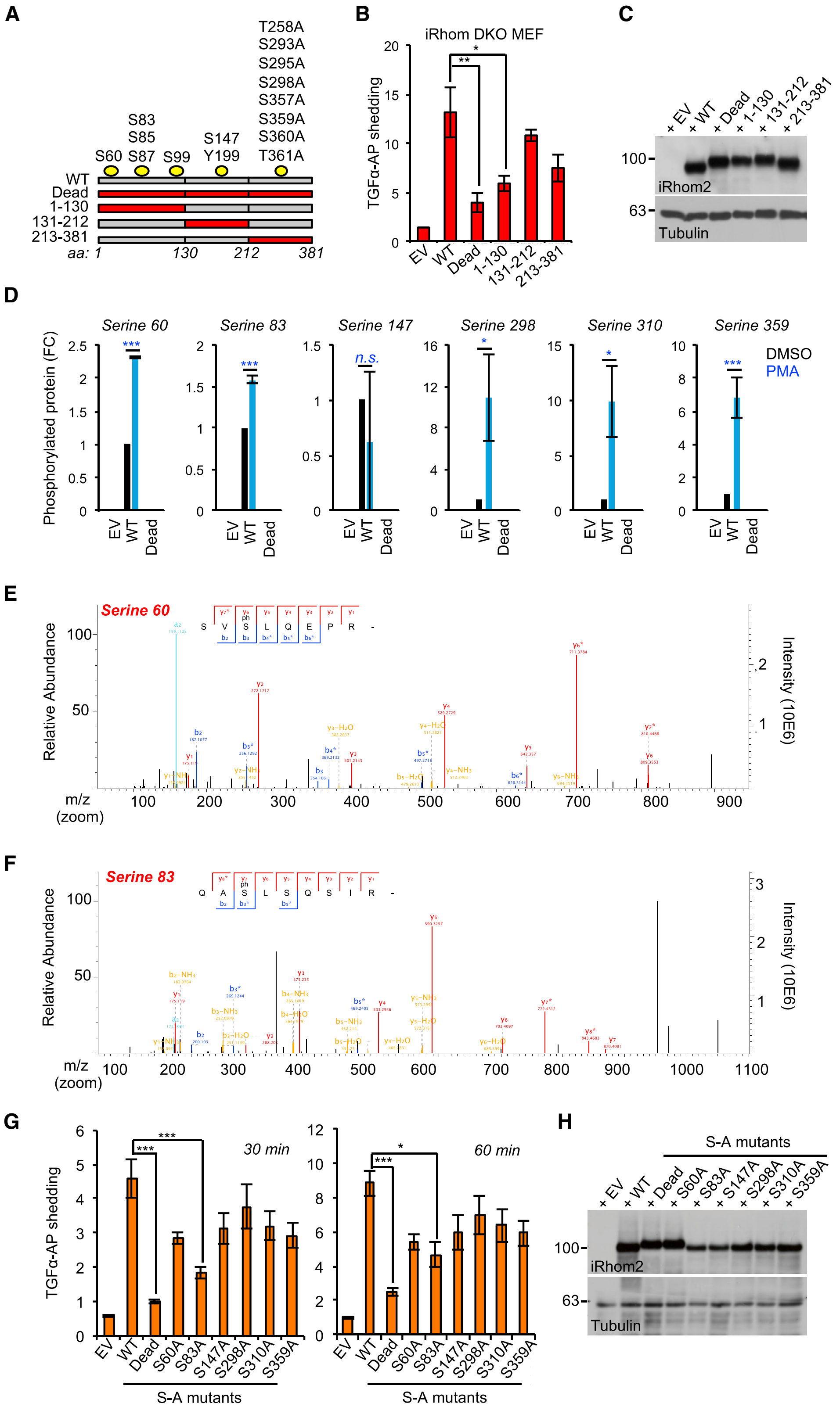

(legend on next page)

that it contained the functionally relevant phosphorylation sites (Figure 4B). The 131–212 and 213–381 mutants exhibited a reduced, although not statistically significant, ability to support shedding compared with WT iRhom2, although all mutants were expressed at equivalent levels (Figure 4C).

To obtain deeper insights into the specific residues within the iRhom2 cytoplasmic tail that were phosphorylated in response to PMA in our model, we performed mass spectrometry on immunoprecipitates from HEK293ET cells expressing HA-tagged WT mouse iRhom2 versus the iRhom2 Dead mutant. This revealed that iRhom2 was phosphorylated at multiple serines upon PMA stimulation (Figure 4D), including two residues, S60 (Figure 4E) and S83 (Figure 4F), that mapped within the 1–130 mutant cluster that significantly impaired PMA-induced shedding (Figure 4B). When we examined each identified phosphorylation site individually, we found that only blocking phosphorylation at S83 significantly impaired shedding (Figure 4G). In contrast, the other mutants including S60A, impaired shedding only modestly, indicating that, at least when scrutinized individually, these residues play a minor role in shedding (Figures 4G and 4H).

To examine the functional impact of iRhom2 phosphorylation, we performed mass spectrometry analysis to determine whether the cohort of iRhom2-interacting proteins changed upon phosphorylation. These experiments revealed 11 proteins, whose binding to iRhom2 was altered upon PMA stimulation, including several 14-3-3 protein isoforms, that function as molecular switches, whose recruitment is often contingent on phosphorylation of their binding proteins (Figures 5A and S1B). The binding of 14-3-3 proteins to iRhom2 appeared plausible because 14-3-3 recruitment motifs (Madeira et al., 2015) are found at phosphoserines S60, S83, and S359 within the iRhom2 cytoplasmic tail (Figure S1A). Co-immunoprecipitation experiments confirmed that 14-3-3 proteins were recruited to iRhom2 upon PMA stimulation (Figure 5B) dependent on phosphorylation at S83 (Figure 5C). As S83 is important for shedding (Figure 4G), this highlights a connection between the phosphorylation-dependent recruitment of 14-3-3 and TACE stimulation.

To test directly the role of 14-3-3 proteins in TACE stimulation, we constructed a mutant in which the well-characterized 14-3-3 recruitment motif R18 (Masters and Fu, 2001) was added to the N terminus of the cytoplasmic tail of the iRhom2 Dead mutant (Figure 5D). Interestingly, this mutant, which bound to 14-3-3 proteins constitutively in the absence of PMA stimulation (Figure 5E), exhibited a significantly increased capacity to facilitate TACE shedding activity (Figure 5F), without increasing the pool of mature TACE (Figure 5G). Together, our data confirm that 14-3-3 protein recruitment is central to the mechanism whereby iRhom2 phosphorylation at S83 controls TACE stimulation.

iRhom2 Phosphorylation Does Not Control iRhom/TACE Trafficking to the Cell Surface

The recruitment of 14-3-3 proteins can exert distinct effects on the trafficking fate of membrane proteins, including facilitating their exit from the ER, or regulating their endocytosis (O'Kelly et al., 2002; Gabriel et al., 2012). To investigate whether 14-3-3 protein recruitment to phosphorylated iRhom2 affected TACE maturation (which requires its progression into the *trans*-Golgi apparatus) or iRhom2 trafficking itself, we began by examining the ability of iRhom2 phosphorylation mutants to promote TACE trafficking to the *trans*-Golgi apparatus, where the prodomain of TACE is normally cleaved off by furin (Schlöndorff et al., 2000; Adrain et al., 2012). Notably, TACE maturation was not impaired in iRhom DKO MEFs expressing a panel of non-phosphorylatable mutants (Figures 6A and 6B), confirming that iRhom2 phosphorylation does not control TACE trafficking from the ER to the *trans*-Golgi.

Our observations indicate that iRhom2 fulfills two important, but separable, functions: its canonical role in the anterograde trafficking of TACE, versus a distinct function, dependent on iRhom2 phosphorylation, that controls the rapid stimulation of TACE activity. Assuming that the effect elicited by iRhom2 phosphorylation impinges directly on TACE, the corollary is that iRhom2 should be found on the cell surface, where TACE exerts its sheddase activity. Immunofluorescent experiments describe iRhom2 to be an intracellular, predominantly ER-localized protein (Zettl et al., 2011; Maney et al., 2015; Grieve et al., 2017). However, two recent studies have found that overexpressed forms of iRhom localize to the cell surface (Maney et al., 2015; Grieve et al., 2017). We confirmed this result in flow cytometry experiments, in unpermeabilized RAW267.4 murine macrophages stably expressing HA-tagged iRhom2 (Figure 6C). As overexpression can risk artifactual spillover of membrane proteins into other compartments, we investigated this phenomenon more stringently, using a cell-impermeable biotinylation reagent to detect endogenous iRhom2 on the cell surface (Figure 6D). Inhibiting dynamin-dependent endocytosis using dynasore blocked iRhom2 degradation (Figures 6E and 6F), supporting the hypothesis that, like TACE (Lorenzen et al., 2016), the stability of iRhom2 is controlled by the endolysosomal system.

Our observations suggest that iRhom2 hence has the potential to regulate TACE throughout the secretory pathway. However,

Figure 4. Phosphorylation of iRhom2 at Serine 83 Is Essential for TACE Shedding Activity

(A) Schematic of the putative iRhom2 phosphorylated residues that were mutated to alanine.

(B) The putative phosphorylated residues located within amino acids 1–130 of iRhom2 are required for TACE shedding of TGF-α-AP in rescue assays in iRhom1/2 DKO MEFs stably expressing the specified mutants. PMA (60 min) was used to stimulate shedding. EV, empty vector.

(C) The iRhom2 non-phosphorylatable mutants (used in B) had equivalent expression levels in iRhom1/2 DKO MEFs.

(D) Mass spectrometry analysis identified six phosphorylation sites in mouse iRhom2-HA, stably expressed in HEK293ET cells. Five of those identified are PMA inducible (15 min PMA). FC, fold change relative to WT cells treated with DMSO. n = 2, Student's t test.

(E and F) Mass spec analysis of iRhom2 phosphorylation at S60 (E) or S83 (F).

(G) iRhom1/2 DKO MEFs transduced with iRhom2 retrovirus encoding the indicated serine-to-alanine (S/A) single-point mutations PMA (30 and 60 min) was used to stimulate shedding of TGF-α-AP.

(H) The iRhom2 S/A non-phosphorylatable mutants (used in G) had equivalent expression levels in iRhom1/2 DKO MEFs.

Data are presented as mean ± SEM.

OPEN
ACCESS
CellPress

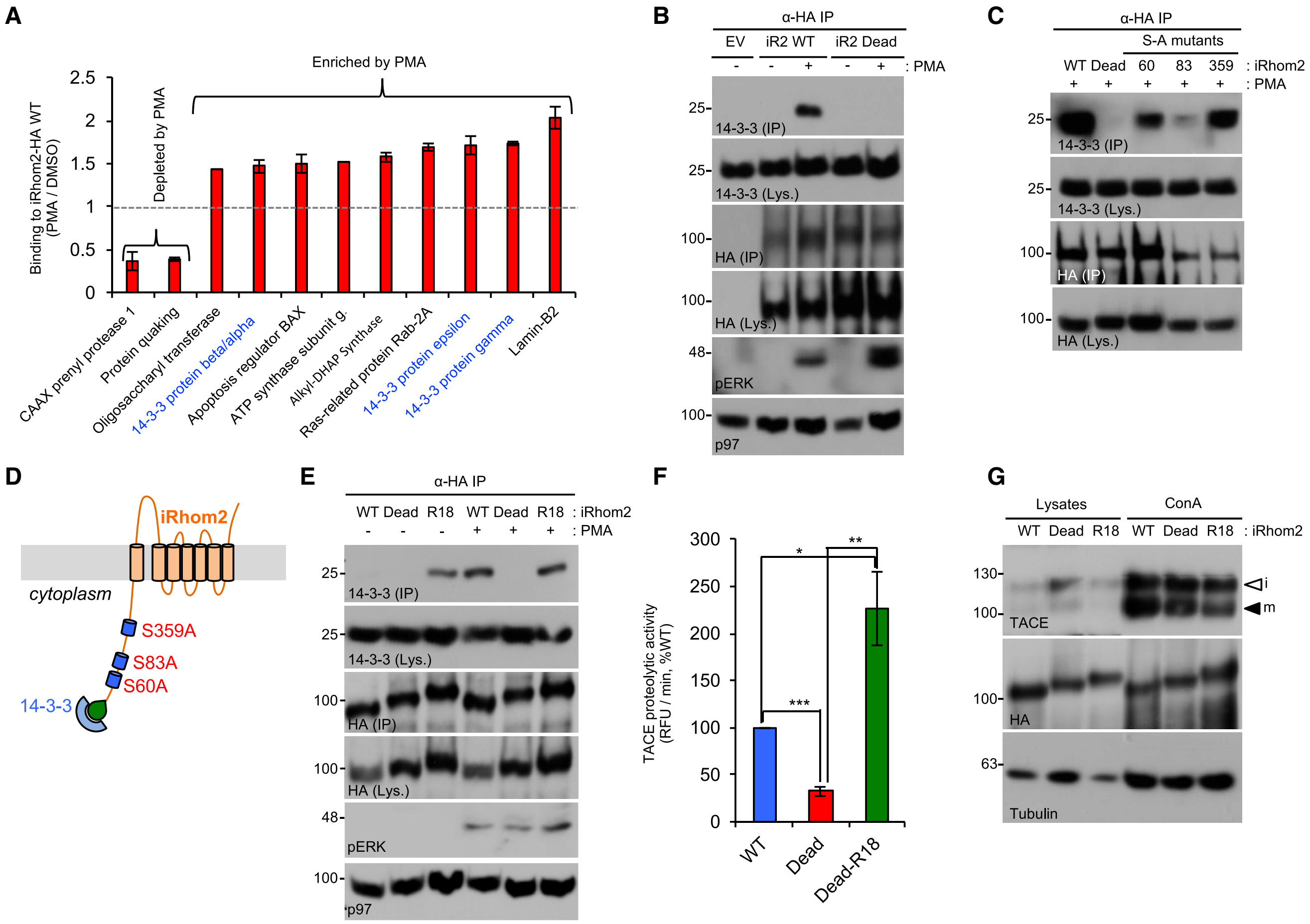

Figure 5. Phosphorylation-Dependent Recruitment of 14-3-3 to iRhom2 Induces Increased TACE Activity

(A) iRhom2-interacting proteins with altered binding upon PMA stimulation, detected by mass spectrometry, in iRhom2-HA immunoprecipitates; 14-3-3 proteins are highlighted in blue. See Figure S1B for a more comprehensive view of this dataset (n = 2).

(B) Validation of PMA-inducible 14-3-3 binding to iRhom2. HEK293ET cells expressing an empty vector (EV) control, WT, or iRhom2-Dead-HA were stimulated (PMA, 30 min). iRhom2-HA was immunoprecipitated and endogenous 14-3-3 binding detected with a pan-14-3-3 antibody.

(C) S83 is the major phosphorylated residue that recruits 14-3-3 proteins. HEK293ET cells expressing iRhom2-HA serine-to-alanine (S/A) mutants at the three putative 14-3-3 binding sites (S60, S83, and S359) were stimulated with PMA (30 min), iRhom2-HA was immunoprecipitated and endogenous 14-3-3 binding detected.

(D) Schematic of the R18-iRhom2 Dead mutant. The R18 peptide (PHCVPRDLSWLDLEANMCLP) was fused to the N terminus of iRhom2 Dead-HA mutant, to confer phosphorylation independent binding of 14-3-3 proteins.

(E) The R18-iRhom2 Dead-HA mutant binds constitutively to endogenous 14-3-3 proteins. HEK293ET cells expressing the indicated plasmids were stimulated (PMA, 30 min).

(F) 14-3-3 binding induces TACE cell surface proteolytic activity. HEK293ET cells overexpressing the indicated plasmids were left untreated. TACE proteolytic activity on the cell surface was determined by measuring its ability to cleave a fluorogenic substrate. RFU, relative fluorescent units. Student's t test.

(G) TACE maturation is not affected by 14-3-3 binding. Lysates from iRhom1/2 DKO MEFs stably expressing the indicated plasmids were ConA enriched and immunoblotted for TACE.

Data are presented as mean ± SEM. See also Figure S1.

the amounts of cell surface iRhom2 and TACE appeared equivalent in cells expressing WT versus the Dead mutant (Figures S2A–SC), suggesting that iRhom2 phosphorylation does not control the trafficking of iRhom2 or TACE to the cell surface. Moreover, the rate of PMA-triggered loss of TACE from the cell surface was not significantly different (Figures S2A and S2B), and dynasore did not rescue shedding in the iRhom2 Dead mutant, confirming that defective endocytosis or sorting to the lysosome could not account for the TACE shedding defect (Figure S2D).

An important implication of these results is that the phosphorylation mutants of iRhom2 are not misfolded, because the iRhom2 Dead mutant was fully capable of supporting the trafficking of itself and TACE throughout the secretory pathway. By contrast, if iRhom2 phosphorylation mutants were misfolded, their retention in the ER and degradation by ER-associated

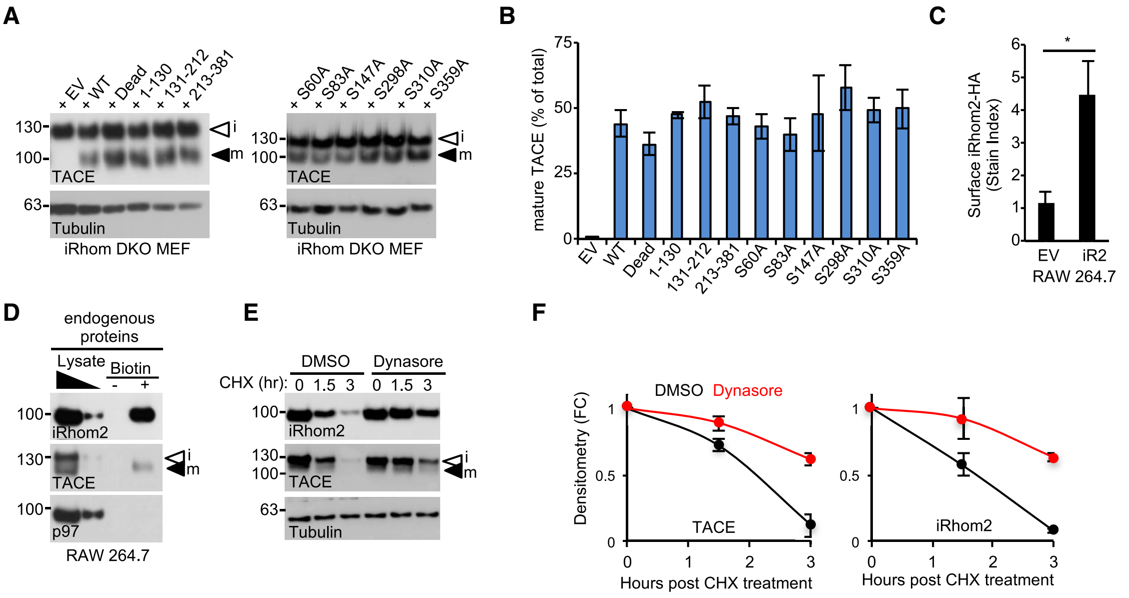

Figure 6. iRhom2 Phosphorylation Regulates TACE Beyond the ER

(A) TACE maturation is not affected by iRhom2 phosphorylation in iRhom1/2 DKO MEFs stably expressing the indicated iRhom2 mutants.

(B) Densitometric scans illustrating the proportion of mature TACE in iRhom DKO MEFs expressing the indicated iRhom2 mutants.

(C) Flow cytometric detection of overexpressed cell surface iRhom2-HA in non-permeabilized RAW264.7 cells. Student's t test. EV, empty vector.

(D) Cell surface biotinylation assays detecting endogenous cell surface iRhom2 in RAW264.7 macrophages.

(E) Consistent with cell surface localization, endogenous iRhom2 degradation upon treatment with a protein synthesis inhibitor (CHX, 50 μg/mL) is rescued by the inhibitor of dynamin-dependent endocytosis dynasore (80 μM, co-treatment with CHX).

(F) Densitometry of (D).

Data are presented as mean ± SEM. See also Figure S2 and Table S2.

degradation (ERAD) would cause the loss of their essential TACE trafficking function, blocking TACE maturation. Further evidence that the iRhom2 Dead mutant is properly folded came from mass spectrometry analysis of iRhom2 interacting proteins, revealing that, apart from 14-3-3 proteins (as anticipated), the majority (>93%) of proteins that interact with WT iRhom2 also bound the iRhom2 Dead mutant (Table S2; Figures S1C and S1D). Hence, blocking iRhom2 phosphorylation causes a specific defect in the biology of TACE stimulation, without affecting the biogenesis or trafficking of iRhom2 and TACE.

iRhom2 Phosphorylation Enhances TACE Activity Independently of Substrate Recruitment by Promoting Dissociation of TACE from iRhom2

As previously proposed (Lemberg and Adrain, 2016), our model implies that iRhom2 remains associated with TACE at the cell surface. As it has been suggested that stimulation controls exposure of TACE's active site (Le Gall et al., 2010), we hypothesized that iRhom2 phosphorylation may be the mechanism that facilitates TACE stimulation by exposing the TACE proteolytic site to its substrates. To test this, we used an assay using a fluorogenic TACE substrate added to the media of PMA-treated cells. The assay specifically measured TACE activity upon iRhom2 overexpression, because a TACE-specific short hairpin RNA (shRNA) abrogated peptide cleavage, whereas knockdown of ADAM10, TACE's closest relative, did not affect peptide hydrolysis (Figures S3A and S3B). Moreover, the assay was sensitive to inhibition by the metalloprotease inhibitor BB94, but not GI254023X, which has a 100-fold preference for ADAM10 over TACE (Figures S3C and S3D). Compared with cells expressing WT iRhom2, TACE activity in cells expressing the iRhom2 Dead mutant were defective in stimulated TACE proteolytic activity (Figures 7A and 7B). This is significant because it rules out the possibility that iRhom2 phosphorylation is required for the recruitment of transmembrane TACE substrates.

To assess the basis for how phosphorylation of iRhom2 rendered TACE more accessible to substrates in more detail, we focused on the iRhom2 Dead mutant containing the "R18" 14-3-3 recruitment motif, which constitutively binds to 14-3-3 (Figure 5E) and rescues the loss of function phenotype associated with the iRhom2 Dead mutant (Figure 5F). Similar to a recent observation that iRhom phosphorylation promotes dissociation of TACE from iRhom2 on the cell surface (Grieve et al., 2017), we observed substantially reduced binding of mature TACE to the R18-iRhom2 Dead mutant (Figure 7C). We also observed a trend suggesting that the Dead mutant retained TACE more efficiently than WT iRhom2 in response to PMA stimulation (Figure 7C).

We next focused on the mechanism whereby 14-3-3 recruitment triggers dissociation of TACE from iRhom2. As Grieve et al. (2017) proposed that the cytoplasmic tail of iRhom2 was specifically required for the binding to mature TACE, we examined which portions of TACE are needed for interaction with

OPEN ACCESS
CellPress

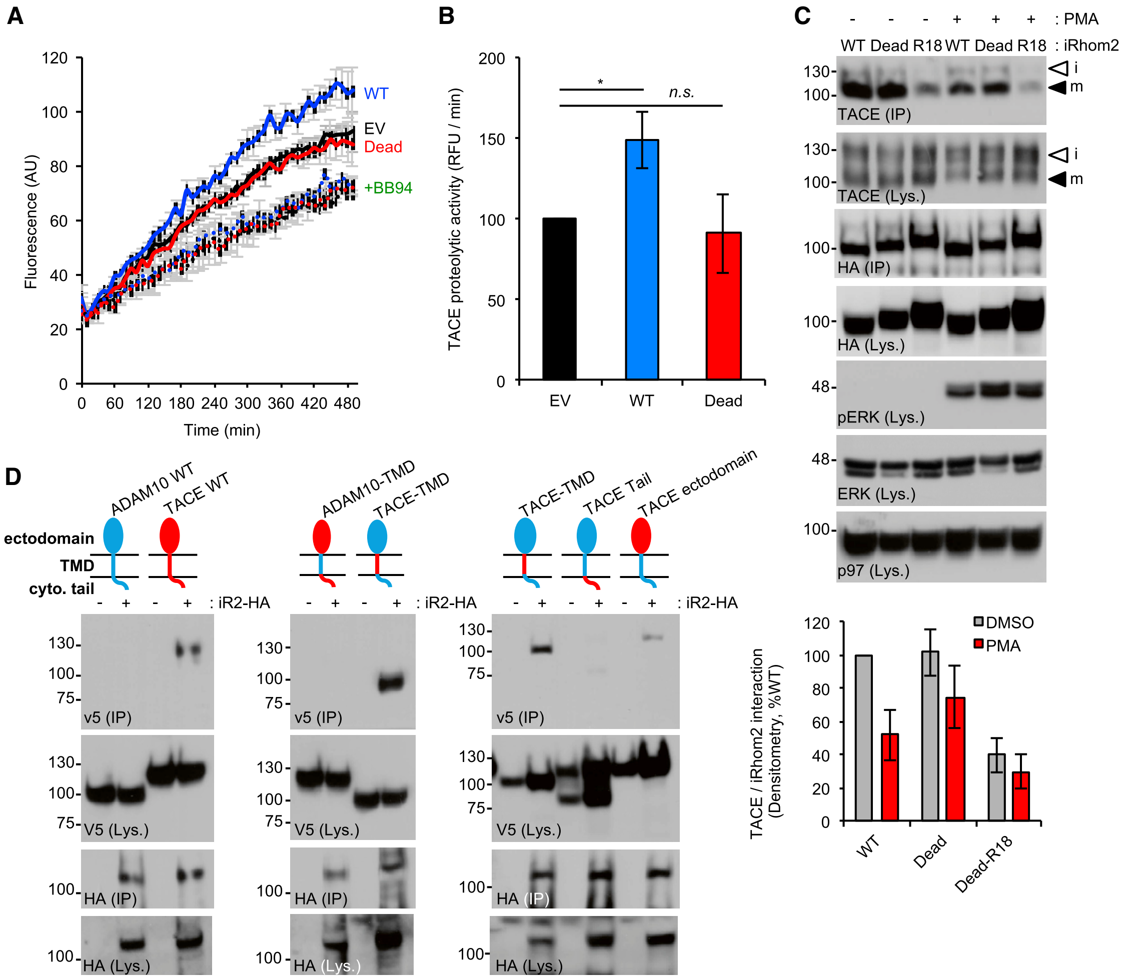

Figure 7. iRhom2 Phosphorylation Regulates TACE Proteolytic Activity at the Cell Surface Independently of Substrate Delivery

(A) iRhom2-HA WT overexpression in HEK293ET cells, but not iRhom2-HA Dead, increases the ability of TACE to cleave a fluorogenic substrate added to the culture media of PMA-stimulated cells. The metalloprotease inhibitor Batimastat (BB94) demonstrates the component of TACE activity conferred by iRhom2 overexpression. AU, arbitrary units.

(B) TACE cell surface proteolytic activity expressed as the rate of peptide cleavage (relative fluorescent units [RFU] per minute). Student's t test.

(C) 14-3-3 binding induces dissociation of the iRhom2/TACE complex. HEK293ET cells expressing the indicated plasmids were stimulated (PMA, 30 min). Dissociation of iRhom2/TACE was assessed by α-HA immunoprecipitates. Bottom: densitometric quantification of the interaction between TACE and iRhom2, as a percentage of binding to iRhom2 WT under non-stimulated conditions.

(D) Plasmids encoding WT V5 tagged TACE, ADAM10, or the indicated domain swap chimeras of TACE (red) and ADAM10 (blue) were transfected into HEK293ET together with iRhom2-HA (+) or empty vector (–). Binding of TACE, ADAM10, or chimeric constructs to iRhom2-HA was detected using anti-V5 antibody on anti-HA IPs.

Data are presented as mean ± SEM. See also Figure S3.

iRhom2 in co-immunoprecipitations. Significantly, we found that the TACE transmembrane domain was the principal determinant of binding to iRhom2: replacing the ADAM10 transmembrane domain with that of TACE conferred binding of this chimeric construct to iRhom2 (Figure 7D). By contrast, an ADAM10 chimera containing the TACE cytoplasmic tail could not bind to iRhom2, implying, albeit indirectly, that the iRhom2 tail does not play a major role in binding to the TACE tail (Figure 7D). Together, we conclude that iRhom2 phosphorylation recruits 14-3-3 proteins, enforcing dissociation of iRhom from TACE, via a mechanism dependent on transmembrane domain interactions, and rendering the active site of TACE more accessible to its substrates.

DISCUSSION

Our data highlight the important regulatory role of the iRhom cytoplasmic tail and suggests that it is an important signaling hub. It is interesting to note that the iRhom2 cytoplasmic tail contains predicted disordered segments (Figure S1A). Such regions are often rich in protein interaction surfaces, including 14-3-3 binding sites, and are highly sensitive to input

from signaling pathways, including phosphorylation-induced conformational changes (Bozoky et al., 2013).

During the revision of our manuscript, Grieve et al. (2017) published findings that reach similar conclusions to ours, although our data differ from, and extend, those observations in several ways. Our work establishes a physiological context in which iRhom phosphorylation occurs, placing it within the settings of infection (activation of Toll-like receptors by bacteria and viruses) and cancer (EGFR transactivation by G protein-coupled receptors). Our objective identification of the phosphorylation sites within iRhom2 is complementary to Grieve et al. (2017): whereas our work maps the phosphorylated residues induced upon TACE stimulation in mouse iRhom2, Grieve et al. (2017) documented basal phosphorylation of residues within human iRhom2.

Grieve et al. (2017) propose that the cytoplasmic tail of iRhom2 interacts with mature TACE. Arguing against this, our data show that the transmembrane domain of TACE is necessary and sufficient for binding to iRhom2, whereas the TACE cytoplasmic tail is not. Moreover, although the TACE cytoplasmic tail is dispensable for stimulation, its transmembrane domain cannot be substituted (Le Gall et al., 2010); single-point mutants within TACE transmembrane domain impair stimulated shedding (Li et al., 2017). Our interpretation is that recruitment of 14-3-3 proteins to the iRhom2 tail triggers a cue that is transduced through iRhom2, to weaken transmembrane interactions between TACE and iRhom2, releasing TACE from the complex, facilitating its access to substrates.

It is important to relate our observations to the mechanism of TACE stimulation proposed by Sommer et al. (2016), who showed that the membrane proximal domain of TACE has an affinity for the phospholipid PS. TACE-activating stimuli trigger PS externalization; the TACE membrane proximal domain binds PS, provoking a conformational change, drawing TACE in closer juxtaposition to its substrates (Sommer et al., 2016). Notably, as blocking this PS-dependent mechanism impairs the cleavage of transmembrane TACE substrates, but does not affect peptide substrate hydrolysis (Sommer et al., 2016), it implies that our iRhom-dependent mechanism is distinct from this. An additional potential mechanism that has been proposed to explain the stimulation mechanism involves the stimulus-induced dissociation of the metalloprotease inhibitor TIMP3 from TACE. However, as the extent of TACE stimulation is identical in TIMP3 null cells as in WT cells (Le Gall et al., 2010), we consider this possibility unlikely.

There is evidence that iRhoms can govern the substrate specificity of TACE (Maretzky et al., 2013), and Grieve et al. (2017) noted the possibility that phosphorylation of iRhom2 could influence substrate selection. We do not rule out the possibility that some of the multiple phosphorylation sites within iRhoms may influence substrate recruitment. However, because our phosphorylation-defective iRhom2 Dead mutant cannot support TACE cleavage of a peptide substrate, it suggests that a failure to recruit transmembrane substrate to TACE is not the basis of the defect.

Our observation that iRhom2 is at the core of the cell surface shedding regulatory machinery reconciles the ambiguity that TACE stimulation involves p38 and ERK kinases (Díaz-Rodríguez et al., 2002; Fan et al., 2003; Scott et al., 2011; Soond et al., 2005; Xu et al., 2012; Fan and Derynck, 1999), yet its tail is dispensable for stimulation (Le Gall et al., 2010; Hall and Blobel, 2012). Consistent with a potential role for MAP kinases, the cytoplasmic tail of iRhom2 contains two predicted ERK-D boxes (Figure S1A). Similarly, our immunoprecipitation and mass spectrometry experiments (Figure S4) detected the binding of kinases to iRhom1 and iRhom2, including ERK1, ERK2, and p38α, as well as binding of RSK1 and RSK2 to iRhom2. However, we cannot conclude that direct phosphorylation of iRhom2 by MAP kinases is key to the TACE stimulation mechanism. From our analyses, the single most important residue in iRhom2 for stimulated shedding is serine 83, which harbors the site [K/R]-XX-[S/T] recognized by the AGC kinase family members, such as RSK kinases, that are activated downstream of ERK/p38 (Roux and Blenis, 2004; Pearce et al., 2010). iRhom2 is phosphorylated in multiple residues; presumably several phosphorylation sites collectively make additive contributions, as demonstrated by the iRhom2 Dead mutant, whose function is more profoundly blocked than the S83A point mutant. Overall, we do not exclude the possibility that phosphorylation of iRhom2 by several kinase families control stimulated TACE shedding.

Notably, our mass spectrometry experiments identified that several residues within iRhom2, including S83, exhibit a degree of basal phosphorylation in the absence of stimulation (Figure 4D). This raises the possibility that the basal activity of TACE may also be regulated by iRhom2 phosphorylation.

Our observation that iRhom2 is phosphorylated is an example of the dynamic regulation of rhomboid like proteins in the secretory pathway by signaling pathways. A prior belief, from studies that demonstrated that expression of rhomboid proteases prefigures signaling (Wasserman and Freeman, 1998), was that rhomboid proteins are not subject to post-transcriptional regulation. However, our work reveals that iRhoms are highly regulated molecules. Interestingly, there are many parallels between the regulation of iRhoms by their cytoplasmic tails and the regulation of the important chloride channel, cystic fibrosis transmembrane conductance regulator (CFTR). Like iRhom, CFTR contains a cytoplasmic loop called the "R domain" (regulatory domain) that is highly disordered, contains sites for phosphorylation and 14-3-3 binding, and plays a key role in regulating CFTR ion channel activity (Bozoky et al., 2013). Because of these striking similarities, we propose to name the cytoplasmic tail of iRhoms the regulatory (R) domain (Figure S5). Interestingly, the CFTR R domain has been proposed to act as signaling hub not only for pathways that regulate CFTR but as a general signaling platform. It will be interesting to determine whether the same applies for iRhoms.

The discovery that endogenous iRhom2 localizes to the cell surface, and that its phosphorylation is required for shedding, requires rethinking of the model for iRhom function. Rather than a trafficking role restricted to the ER-to-Golgi transport of TACE, the data now support a model whereby iRhom and TACE traffic to the cell surface as a complex, allowing iRhom to fulfill a role in sensing and transducing shedding stimuli to TACE (Figure S5). Rather than acting as a TACE trafficking factor, iRhom's role could be regarded as analogous to the relationship between the accessory subunits of multi-membrane protein complexes,

for example, gamma secretase, the T cell receptor, or MHC class I. Similar to the examples above, loss of iRhom results in the ER retention of TACE. Additionally, iRhom could be viewed as an allosteric regulator of the TACE complex.

EXPERIMENTAL PROCEDURES

Mice

Experiments with mice were performed in accordance with protocols approved by the IGC Ethics Committee and the National Entity Direção Geral de Alimentação e Veterinária, in accordance with the Portuguese (Decreto-Lei no. 113/2013) and European (directive 2010/63/EU) legislation related to housing, husbandry, and animal welfare. iRhom2$^{-/-}$ (KO) mice were previously reported (Adrain et al., 2012).

Phos-tag Gels and Protein Dephosphorylation

Phos-tag gels were performed as described in Supplemental Experimental Procedures.

Mass Spectrometry Analysis of iRhom2 Phosphorylation and Phosphorylation-Dependent Interactome

HEK293ET cells expressing empty vector, iRhom2-HA WT, or iRhom2 Dead-HA were serum-starved overnight and stimulated with PMA (1 μM) for 15 min. Immunoprecipitations, sample digestion, and mass spectrometry are described in Supplemental Experimental Procedures.

Cell Surface Biotinylation

Biotinylation was performed as previously described for bone marrow-derived macrophages (BMDMs) (Adrain et al., 2012) with small modifications, detailed in Supplemental Experimental Procedures.

Flow Cytometry

RAW264.7 macrophages (1 $\times$ 10^6) expressing empty vector or iRhom2-HA were detached with a cell scraper, Fc-blocked, and stained with an anti-HA primary antibody and Alexa 633-labeled secondary antibody. HEK293ET cells expressing iRhom2-HA WT or iRhom2 Dead-HA were stained with a phycoerythrin-conjugated anti-TACE primary antibody or isotype control phycoerythrin-conjugated IgG1. Analysis details are provided in Supplemental Experimental Procedures.

TACE Enzymatic Assay on Live Cells

The TACE enzymatic assay used an internally quenched fluorogenic peptide based on the TNF cleavage site. Full experimental details are provided in Supplemental Experimental Procedures.

Shedding Assays

MEFs were transfected with plasmids encoding alkaline phosphatase-tagged EGFR ligands TGF-α and BTC (Sahin et al., 2006), as described in Supplemental Experimental Procedures.

Statistical Methods

Unless otherwise stated, all experiments were performed at least three times, and statistical significance was determined using one-way ANOVA with Tukey's multiple comparison testing. Asterisks indicate statistical significance: *$p < 0.05$, **$p < 0.01$, and ***$p < 0.001$.

SUPPLEMENTAL INFORMATION

Supplemental Information includes Supplemental Experimental Procedures, five figures, and two tables and can be found with this article online at https://doi.org/10.1016/j.celrep.2017.09.074.

AUTHOR CONTRIBUTIONS

M.C., I.O., A.K., and C.A. designed experiments. M.C., I.O., E.B., C.J.G., C.G., M.B., T.H., A. Bolado, A. Bileck, A.K., I.F., and P.M.D. carried out experiments and interpreted results. M.C., I.O., and C.A. contributed to the overall design of the study. M.C. and C.A. wrote and all authors edited the manuscript. C.A. supervised the project.

ACKNOWLEDGMENTS

We thank the IGC animal house and flow cytometry facilities. We thank Matthew Freeman for the iRhom2 KO mice and for exchange of reagents and ideas and Haian Fu, Shigeki Higashiyama, Andreas Ludwig, Richard Mulligan, and Anne Ridley for plasmids. We thank Carl Blobel for helpful advice. We thank Tiago Cordeiro and Margarida Amaral for helpful discussions about disordered protein segments. C.A. acknowledges the support of Fundação Calouste Gulbenkian; Worldwide Cancer Research (14-1289); a Marie Curie Career Integration Grant (project no. 618769); Fundação para a Ciência e Tecnologia (FCT) (grants SFRH/BCC/52507/2014 and PTDC/BEX-BCM/3015/2014); and the European Crohn's and Colitis Organization, and COST (BM1406). P.M.D. was supported by FCT grants LISBOA-01-0145-FEDER-007660, FCT-ANR/NEU-NMC/0006/2013, PTDC/NEU-NMC/2459/2014, and IF/00697/2014. M.C. acknowledges the support of the FCT (grant SFRH/BPD/117216/2016).

Received: April 8, 2017
Revised: July 12, 2017
Accepted: September 22, 2017
Published: October 17, 2017

REFERENCES

Adrain, C., and Freeman, M. (2012). New lives for old: evolution of pseudoenzyme function illustrated by iRhoms. Nat. Rev. Mol. Cell Biol. *13*, 489–498.

Adrain, C., Zettl, M., Christova, Y., Taylor, N., and Freeman, M. (2012). Tumor necrosis factor signaling requires iRhom2 to promote trafficking and activation of TACE. Science *335*, 225–228.

Arribas, J., Coodly, L., Vollmer, P., Kishimoto, T.K., Rose-John, S., and Massagué, J. (1996). Diverse cell surface protein ectodomains are shed by a system sensitive to metalloprotease inhibitors. J. Biol. Chem. *271*, 11376–11382.

Blaydon, D.C., Etheridge, S.L., Risk, J.M., Hennies, H.C., Gay, L.J., Carroll, R., Plagnol, V., McRonald, F.E., Stevens, H.P., Spurr, N.K., et al. (2012). RHBDF2 mutations are associated with tylosis, a familial esophageal cancer syndrome. Am. J. Hum. Genet. *90*, 340–346.

Bozoky, Z., Krzeminski, M., Chong, P.A., and Forman-Kay, J.D. (2013). Structural changes of CFTR R region upon phosphorylation: a plastic platform for intramolecular and intermolecular interactions. FEBS J. *280*, 4407–4416.

Brandl, K., Sun, L., Neppl, C., Siggs, O.M., Le Gall, S.M., Tomisato, W., Li, X., Du, X., Maennel, D.N., Blobel, C.P., and Beutler, B. (2010). MyD88 signaling in nonhematopoietic cells protects mice against induced colitis by regulating specific EGF receptor ligands. Proc. Natl. Acad. Sci. U S A *107*, 19967–19972.

Christensen, G.L., Kelstrup, C.D., Lyngsø, C., Sarwar, U., Bøgebo, R., Sheikh, S.P., Gammeltoft, S., Olsen, J.V., and Hansen, J.L. (2010). Quantitative phosphoproteomics dissection of seven-transmembrane receptor signaling using full and biased agonists. Mol. Cell. Proteomics *9*, 1540–1553.

Christova, Y., Adrain, C., Bambrough, P., Ibrahim, A., and Freeman, M. (2013). Mammalian iRhoms have distinct physiological functions including an essential role in TACE regulation. EMBO Rep. *14*, 884–890.

Cohen, P. (2000). The regulation of protein function by multisite phosphorylation—a 25 year update. Trends Biochem. Sci. *25*, 596–601.

Czepielewski, R.S., Porto, B.N., Rizzo, L.B., Roesler, R., Abujamra, A.L., Pinto, L.G., Schwartsmann, G., Cunha, Fde.Q., and Bonorino, C. (2012). Gastrin-releasing peptide receptor (GRPR) mediates chemotaxis in neutrophils. Proc. Natl. Acad. Sci. U S A *109*, 547–552.

Díaz-Rodríguez, E., Montero, J.C., Esparís-Ogando, A., Yuste, L., and Pandiella, A. (2002). Extracellular signal-regulated kinase phosphorylates tumor necrosis factor alpha-converting enzyme at threonine 735: a potential role in regulated shedding. Mol. Biol. Cell *13*, 2031–2044.

Düsterhöft, S., Jung, S., Hung, C.W., Tholey, A., Sönnichsen, F.D., Grötzinger, J., and Lorenzen, I. (2013). Membrane-proximal domain of a disintegrin and metalloprotease-17 represents the putative molecular switch of its shedding activity operated by protein-disulfide isomerase. J. Am. Chem. Soc. *135*, 5776–5781.

Fan, H., and Derynck, R. (1999). Ectodomain shedding of TGF-alpha and other transmembrane proteins is induced by receptor tyrosine kinase activation and MAP kinase signaling cascades. EMBO J. *18*, 6962–6972.

Fan, H., Turck, C.W., and Derynck, R. (2003). Characterization of growth factor-induced serine phosphorylation of tumor necrosis factor-alpha converting enzyme and of an alternatively translated polypeptide. J. Biol. Chem. *278*, 18617–18627.

Gabriel, L., Lvov, A., Orthodoxou, D., Rittenhouse, A.R., Kobertz, W.R., and Melikian, H.E. (2012). The acid-sensitive, anesthetic-activated potassium leak channel, KCNK3, is regulated by 14-3-3β-dependent, protein kinase C (PKC)-mediated endocytic trafficking. J. Biol. Chem. *287*, 32354–32366.

Goldsmith, Z.G., and Dhanasekaran, D.N. (2007). G protein regulation of MAPK networks. Oncogene *26*, 3122–3142.

Gooz, M. (2010). ADAM-17: the enzyme that does it all. Crit. Rev. Biochem. Mol. Biol. *45*, 146–169.

Grieve, A.G., Xu, H., Künzel, U., Bambrough, P., Sieber, B., and Freeman, M. (2017). Phosphorylation of iRhom2 at the plasma membrane controls mammalian TACE-dependent inflammatory and growth factor signalling. eLife *6*, 6.

Hall, K.C., and Blobel, C.P. (2012). Interleukin-1 stimulates ADAM17 through a mechanism independent of its cytoplasmic domain or phosphorylation at threonine 735. PLoS ONE *7*, e31600.

Horiuchi, K., Kimura, T., Miyamoto, T., Takaishi, H., Okada, Y., Toyama, Y., and Blobel, C.P. (2007). Cutting edge: TNF-alpha-converting enzyme (TACE/ADAM17) inactivation in mouse myeloid cells prevents lethality from endotoxin shock. J. Immunol. *179*, 2686–2689.

Le Gall, S.M., Maretzky, T., Issuree, P.D., Niu, X.D., Reiss, K., Saftig, P., Khokha, R., Lundell, D., and Blobel, C.P. (2010). ADAM17 is regulated by a rapid and reversible mechanism that controls access to its catalytic site. J. Cell Sci. *123*, 3913–3922.

Lemberg, M.K., and Adrain, C. (2016). Inactive rhomboid proteins: new mechanisms with implications in health and disease. Semin. Cell Dev. Biol. *60*, 29–37.

Li, X., Maretzky, T., Perez-Aguilar, J.M., Monette, S., Weskamp, G., Le Gall, S., Beutler, B., Weinstein, H., and Blobel, C.P. (2017). Structural modeling defines transmembrane residues in ADAM17 that are crucial for Rhbdf2/ADAM17-dependent proteolysis. J. Cell Sci. *130*, 868–878.

Lorenzen, I., Lokau, J., Korpys, Y., Oldefest, M., Flynn, C.M., Künzel, U., Garbers, C., Freeman, M., Grötzinger, J., and Düsterhöft, S. (2016). Control of ADAM17 activity by regulation of its cellular localisation. Sci. Rep. *6*, 35067.

Ludwig, A., Hundhausen, C., Lambert, M.H., Broadway, N., Andrews, R.C., Bickett, D.M., Leesnitzer, M.A., and Becherer, J.D. (2005). Metalloproteinase inhibitors for the disintegrin-like metalloproteinases ADAM10 and ADAM17 that differentially block constitutive and phorbol ester-inducible shedding of cell surface molecules. Comb. Chem. High Throughput Screen. *8*, 161–171.

Madeira, F., Tinti, M., Murugesan, G., Berrett, E., Stafford, M., Toth, R., Cole, C., MacKintosh, C., and Barton, G.J. (2015). 14-3-3-Pred: improved methods to predict 14-3-3-binding phosphopeptides. Bioinformatics *31*, 2276–2283.

Maney, S.K., McIlwain, D.R., Polz, R., Pandyra, A.A., Sundaram, B., Wolff, D., Ohishi, K., Maretzky, T., Brooke, M.A., Evers, A., et al. (2015). Deletions in the cytoplasmic domain of iRhom1 and iRhom2 promote shedding of the TNF receptor by the protease ADAM17. Sci. Signal. *8*, ra109.

Maretzky, T., McIlwain, D.R., Issuree, P.D., Li, X., Malapeira, J., Amin, S., Lang, P.A., Mak, T.W., and Blobel, C.P. (2013). iRhom2 controls the substrate selectivity of stimulated ADAM17-dependent ectodomain shedding. Proc. Natl. Acad. Sci. USA. *110*, 11433–11438.

Masters, S.C., and Fu, H. (2001). 14-3-3 Proteins mediate an essential anti-apoptotic signal. J. Biol. Chem. *276*, 45193–45200.

O'Kelly, I., Butler, M.H., Zilberberg, N., and Goldstein, S.A. (2002). Forward transport. 14-3-3 binding overcomes retention in endoplasmic reticulum by dibasic signals. Cell *111*, 577–588.

Oda, K., and Kitano, H. (2006). A comprehensive map of the toll-like receptor signaling network. Mol. Syst. Biol. *2*, 2006.0015.

Pearce, L.R., Komander, D., and Alessi, D.R. (2010). The nuts and bolts of AGC protein kinases. Nat. Rev. Mol. Cell Biol. *11*, 9–22.

Peschon, J.J., Slack, J.L., Reddy, P., Stocking, K.L., Sunnarborg, S.W., Lee, D.C., Russell, W.E., Castner, B.J., Johnson, R.S., Fitzner, J.N., et al. (1998). An essential role for ectodomain shedding in mammalian development. Science *282*, 1281–1284.

Prenzel, N., Zwick, E., Daub, H., Leserer, M., Abraham, R., Wallasch, C., and Ullrich, A. (1999). EGF receptor transactivation by G-protein-coupled receptors requires metalloproteinase cleavage of proHB-EGF. Nature *402*, 884–888.

Rousseau, S., Papoutsopoulou, M., Symons, A., Cook, D., Lucocq, J.M., Prescott, A.R., O'Garra, A., Ley, S.C., and Cohen, P. (2008). TPL2-mediated activation of ERK1 and ERK2 regulates the processing of pre-TNF alpha in LPS-stimulated macrophages. J. Cell Sci. *121*, 149–154.

Roux, P.P., and Blenis, J. (2004). ERK and p38 MAPK-activated protein kinases: a family of protein kinases with diverse biological functions. Microbiol. Mol. Biol. Rev. *68*, 320–344.

Sahin, U., Weskamp, G., Kelly, K., Zhou, H.M., Higashiyama, S., Peschon, J., Hartmann, D., Saftig, P., and Blobel, C.P. (2004). Distinct roles for ADAM10 and ADAM17 in ectodomain shedding of six EGFR ligands. J. Cell Biol. *164*, 769–779.

Sahin, U., Weskamp, G., Zheng, Y., Chesneau, V., Horiuchi, K., and Blobel, C.P. (2006). A sensitive method to monitor ectodomain shedding of ligands of the epidermal growth factor receptor. Methods Mol. Biol. *327*, 99–113.

Schlöndorff, J., Becherer, J.D., and Blobel, C.P. (2000). Intracellular maturation and localization of the tumour necrosis factor alpha convertase (TACE). Biochem. J. *347*, 131–138.

Scott, A.J., O'Dea, K.P., O'Callaghan, D., Williams, L., Dokpesi, J.O., Tatton, L., Handy, J.M., Hogg, P.J., and Takata, M. (2011). Reactive oxygen species and p38 mitogen-activated protein kinase mediate tumor necrosis factor α-converting enzyme (TACE/ADAM-17) activation in primary human monocytes. J. Biol. Chem. *286*, 35466–35476.

Siggs, O.M., Xiao, N., Wang, Y., Shi, H., Tomisato, W., Li, X., Xia, Y., and Beutler, B. (2012). iRhom2 is required for the secretion of mouse TNFα. Blood *119*, 5769–5771.

Sommer, A., Kordowski, F., Büch, J., Maretzky, T., Evers, A., Andrä, J., Düsterhöft, S., Michalek, M., Lorenzen, I., Somasundaram, P., et al. (2016). Phosphatidylserine exposure is required for ADAM17 sheddase function. Nat. Commun. *7*, 11523.

Soond, S.M., Everson, B., Riches, D.W., and Murphy, G. (2005). ERK-mediated phosphorylation of Thr735 in TNFalpha-converting enzyme and its potential role in TACE protein trafficking. J. Cell Sci. *118*, 2371–2380.

Steinberg, S.F. (2008). Structural basis of protein kinase C isoform function. Physiol. Rev. *88*, 1341–1378.

Wasserman, J.D., and Freeman, M. (1998). An autoregulatory cascade of EGF receptor signaling patterns the Drosophila egg. Cell *95*, 355–364.

Weintz, G., Olsen, J.V., Frühauf, K., Niedzielska, M., Amit, I., Jantsch, J., Mages, J., Frech, C., Dölken, L., Mann, M., and Lang, R. (2010). The phosphoproteome of toll-like receptor-activated macrophages. Mol. Syst. Biol. *6*, 371.

Wetzker, R., and Böhmer, F.-D. (2003). Transactivation joins multiple tracks to the ERK/MAPK cascade. Nat. Rev. Mol. Cell Biol. *4*, 651–657.

Xu, P., and Derynck, R. (2010). Direct activation of TACE-mediated ectodomain shedding by p38 MAP kinase regulates EGF receptor-dependent cell proliferation. Mol. Cell *37*, 551–566.

Xu, P., Liu, J., Sakaki-Yumoto, M., and Derynck, R. (2012). TACE activation by MAPK-mediated regulation of cell surface dimerization and TIMP3 association. Sci. Signal. *5*, ra34.

Yamamoto, M., Sato, S., Hemmi, H., Sanjo, H., Uematsu, S., Kaisho, T., Hoshino, K., Takeuchi, O., Kobayashi, M., Fujita, T., et al. (2002). Essential role for TIRAP in activation of the signalling cascade shared by TLR2 and TLR4. Nature *420*, 324–329.

Zettl, M., Adrain, C., Strisovsky, K., Lastun, V., and Freeman, M. (2011). Rhomboid family pseudoproteases use the ER quality control machinery to regulate intercellular signaling. Cell *145*, 79–91.

Cell Reports, Volume *21*

Supplemental Information

Phosphorylation of iRhom2 Controls Stimulated Proteolytic Shedding by the Metalloprotease ADAM17/TACE

Miguel Cavadas, Ioanna Oikonomidi, Catarina J. Gaspar, Emma Burbridge, Marina Badenes, Inês Félix, Alfonso Bolado, Tianyi Hu, Andrea Bileck, Christopher Gerner, Pedro M. Domingos, Alex von Kriegsheim, and Colin Adrain

Supplemental Figures

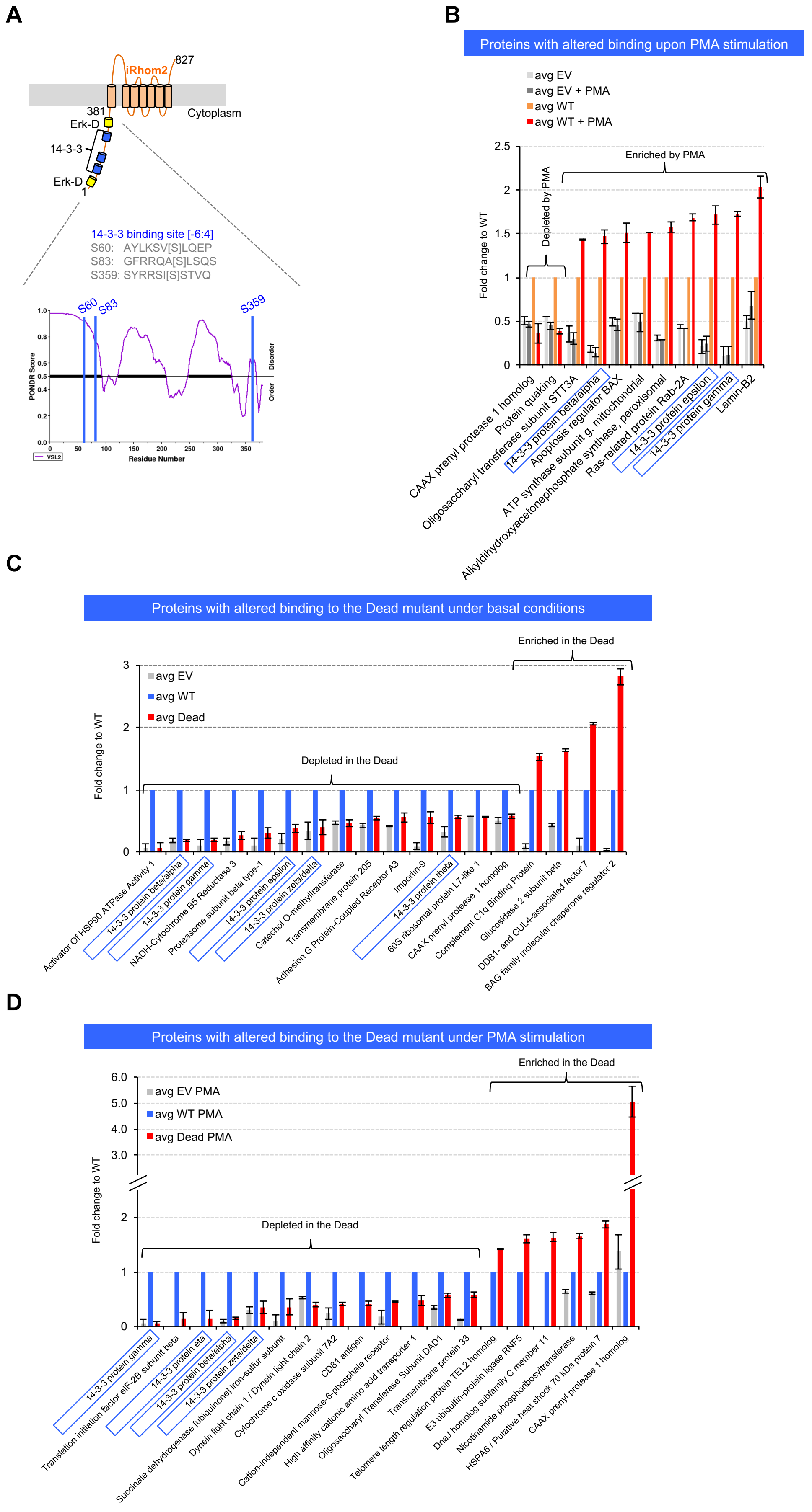

Figure S1. Features within the iRhom2 N-terminus and PMA-stimulated iRhom2-interacting proteins identified by immunoprecipitation/mass spectrometry. Related to Figures 1 and 5. (**A**) The iRhom2 N-terminus has a predicted highly disordered character and contains two predicted ERK docking sites (ERK-D domains), indicated in yellow (aa 19-29 and 334-345). The iRhom2 N-terminus also contains three predicted 14-3-3 binding sites, indicated in blue. Several large-scale phosphoproteomic studies have identified phosphopeptides within the iRhom2 N-terminus, (see Experimental procedures and Table S1 for the specific residues). Several phosphopeptides have been identified, but the kinases responsible are unknown, nor are the functional implications. Details of the prediction algorithms used to characterize features in the iRhom N-terminus are described in Experimental Procedures. (**B**) iRhom2-interacting proteins with altered binding upon PMA stimulation. 14-3-3 proteins are highlighted by a blue box. αHA immunoprecipitates from HEK293 ET cells expressing HA tagged iRhom2-WT or empty vector (EV), were subjected to mass-spec analysis. Interacting proteins whose binding to iRhom2 WT was statistically significantly increased (t-test) over background binding to the immunoprecipitation beads are shown. To increase the stringency, only changes greater than 40% were considered. The same criterion was used to define the interacting proteins whose binding was altered upon PMA stimulation. Proteins with altered binding to the iRhom2 Dead mutant, compared to WT iRhom2 WT, under basal (**C**) and PMA stimulated (**D**) conditions. 14-3-3 proteins are highlighted by a blue box. Anti-HA immunoprecipitates from HEK293 ET cells expressing HA tagged iRhom2 WT or iRhom2 Dead, or empty vector (EV), were subjected to mass-spec analysis. Interacting proteins whose binding to the iRhom2 Dead mutant was enriched or depleted over the WT are shown (using the same criteria as described in the legend of Figure S1B, only hits that were statistically significant and changed at least 40% were considered). Of note, some proteins that were not detected in the WT above the EV levels, were highly enriched in the iRhom2 Dead mutant immunoprecipitates. To identify these hits, datasets were interrogated for proteins with increased binding to the iRhom2 Dead compared to the WT and EV. Throughout, data are presented as mean ± s.e.m. n=2.

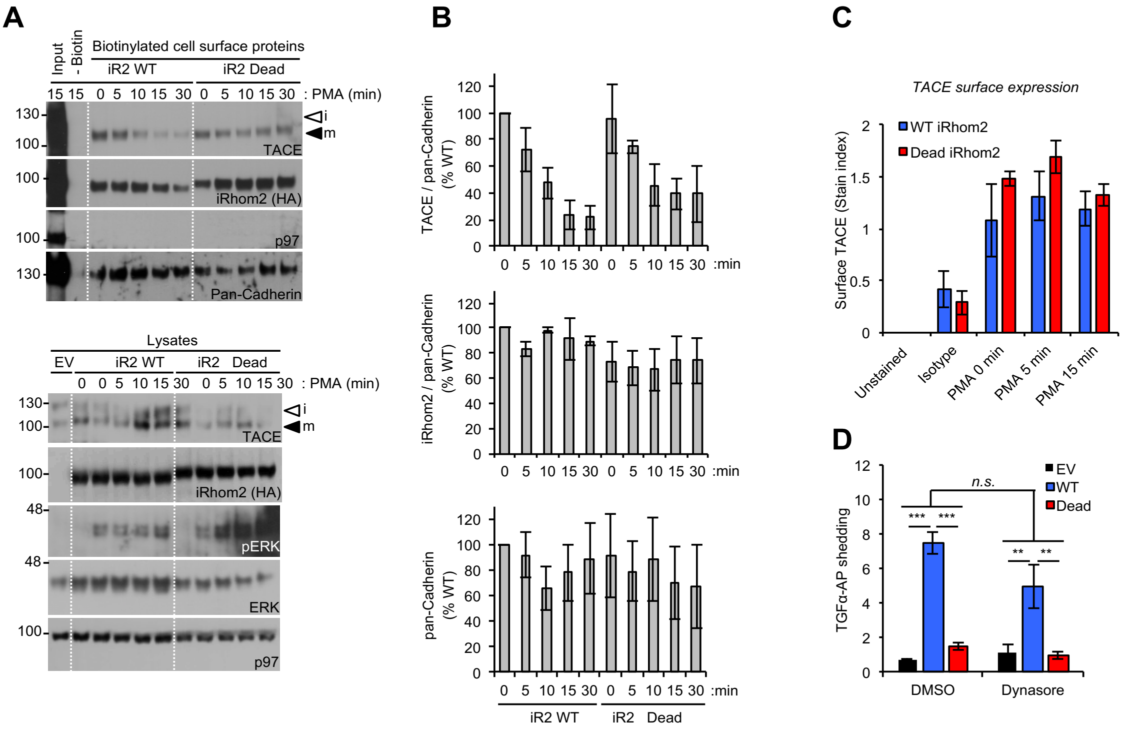

Figure S2. Cell surface expression and PMA induced internalization of TACE are not affected by iRhom2 phosphorylation. Related to Figure 6. (**A**) Biotinylation assays, show that iRhom2 phosphorylation did not affect PMA (1 µM) induced internalization of TACE, in HEK293 ET cells stably expressing iRhom2-HA WT or Dead mutant. (**B**) Densitometry of TACE, iRhom2 and pan-Cadherin bands from 4 replicates of the experiment shown in (A). (**C**) Flow cytometry of non-permeabilized HEK293 ET overexpressing iRhom2-HA WT and Dead constructs to assess cell surface levels of TACE. Cells were stimulated with PMA (1 µM for the indicated periods). (**D**) Defective TGFα-AP shedding in DKO MEFs expressing the iRhom2 Dead mutant is not rescued by the dynamin-dependent endocytosis inhibitor, dynasore. *p<0.05, **p<0.01, ***p<0.001; one-Way ANOVA followed by Tukey's multiple comparisons test. Data are shown as mean ± s.e.m, of at least 3 independent experiments.

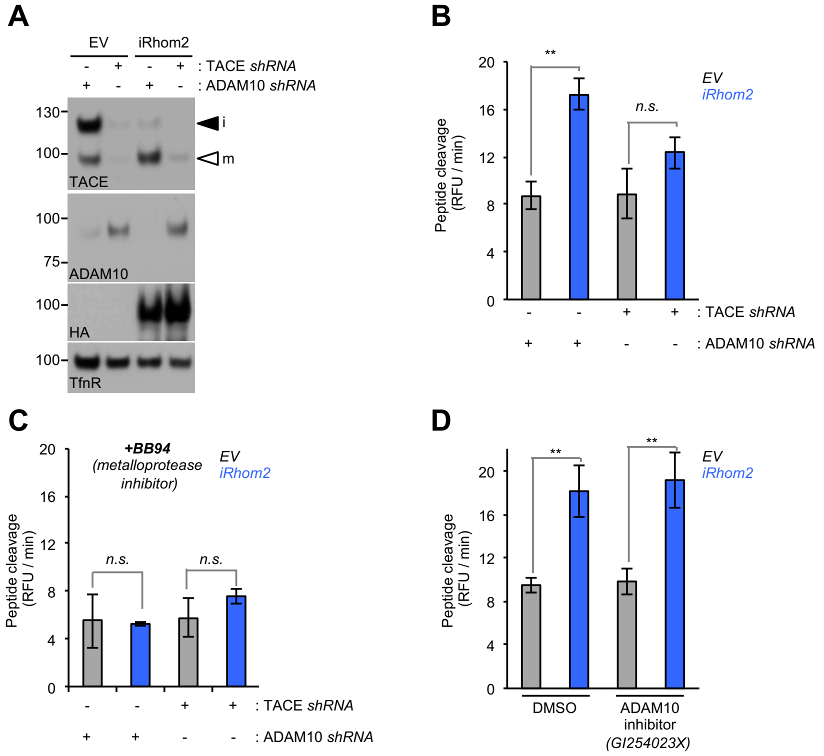

Figure S3. TACE, but not ADAM10, is required for iRhom2-dependent cleavage of a fluorogenic TACE peptide substrate on the cell surface. Related to Figure 7. (**A**) The knockdown of TACE and ADAM10 by lentivirus-delivered shRNA, in HEK293 ET cells expressing empty vector or overexpressing iRhom2-HA, was confirmed by western blotting of ConA enriched cell lysates. The transferrin receptor (TfnR) was used as loading control. (**B**) iRhom2-HA WT overexpression in HEK293 ET cells increases the cleavage of a fluorogenic TACE peptide substrate. The effect is unimpaired by shRNA-mediated knockdown of ADAM10 but is abrogated by knockdown of TACE. (**C**) The metalloprotease inhibitor Batimastat (BB94) inhibits the increased peptide hydrolysis caused by iRhom2-HA overexpression. (**D**) The ADAM10-selective inhibitor GI254023X had no effect on iRhom2 induced peptide cleavage. In B-D, the peptide was added to the culture media of PMA (1 µM) stimulated cells in the presence of BB94 (10 µM) or GI254023X (1 µM) where indicated. Peptide cleavage was expressed as the rate of peptide cleavage (RFU/minute). Data are presented as mean ± s.e.m. **P<0.01, *n.s.* not significant; student's t-test. n =3 independent experiments.

A

	Kinase motif	Kinase group	Kinase family
mouse S60	MLPERRKNPAYLKSV**S**LQEPRGRWQEGAEKR		
	S/T-X-X-E	CMGC	CK2
	K/R-X-X-S/T	AGC	
mouse S83	WQEGAEKRPGFRRQA**S**LSQSIRKSTAQWFGV		
	S-X-X-X-S	CMGC	GSK
	R-X-X-S/T	CAMK	CAMK2
	K/R-X-X-S/T	AGC	
mouse S147	LELPSQEVPSFQGTE**S**PKPCKMPKIVDPLAR		
	S/T-P-K/R	CMGC	CDK
	K/R-X-X-S/T	AGC	
mouse S298	SASYFRGVPHSASPV**S**PDGVHIPLKEYSGGR		
	S-X-X-S/T	CK1	CK1
	P-X-S/T-P	CMGC	MAPK
mouse S310	SPVSPDGVHIPLKEY**S**GGRALGPGTQRGKRI		
	K/R-X-X-S/T	AGC	
mouse S359	GVVGNWLNRSYRRSI**S**STVQRQLESFDSHRP		
	R-X-X-S/T	CAMK	CAMK2
	R-R/S/T-X-S/T-X-S/T	AGC	AKT

B

Kinase group	AGC			CMGC		
Kinase family	RSK		GRK	MAPK	MAPK	MAPK
Protein	RSK1	RSK2	GPCR kinase 6	ERK1	ERK2	p38α
Gene	RPS6KA1	RPS6KA3	GRK6	MAPK3	MAPK1	MAPK14
EV	-	-	-	-	-	-
iR1	+	-	-	+	+	+
iR2	+	+	+	+	+	+

Figure S4. iRhom2 interacts with MAPK and AGC-family kinases. Related to Discussion. (**A**) Kinase motifs present at each phosphorylation site identified in PMA-stimulated HEK293 ET iRhom2-HA expressing cells. (**B**) Kinases interacting with iRhom1 and iRhom2. Data was obtained from mass-spec analysis of immunoprecipitations from HEK293 ET cells expressing HA-tagged iRhom1, iRhom2 versus empty vector (EV) as negative control for binding.

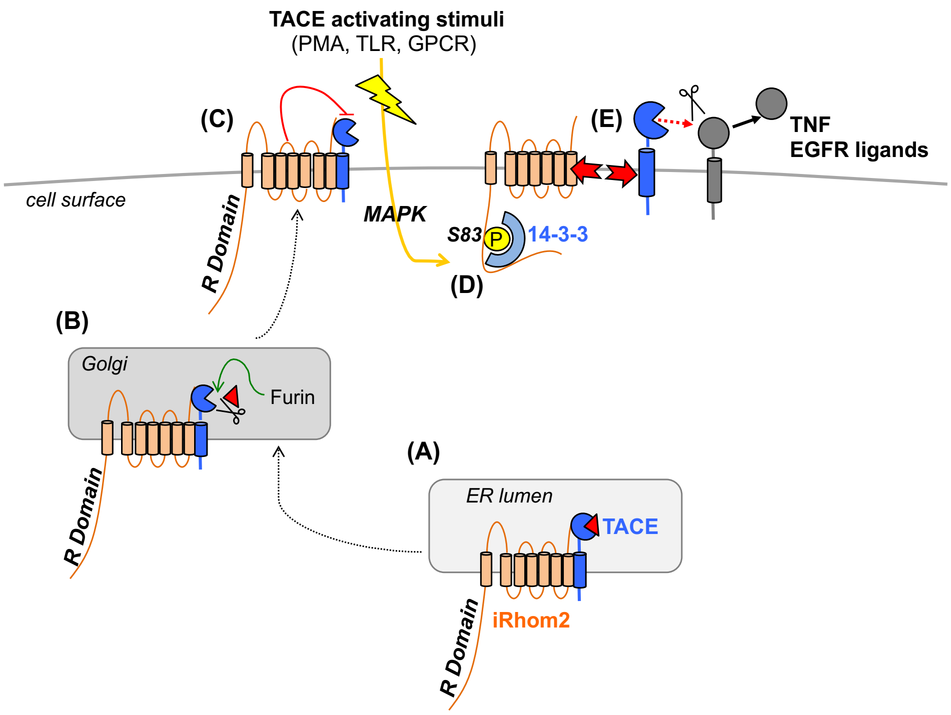

Figure S5. iRhom2 regulates TACE via two distinct mechanisms. Related to Discussion. The schematic depicts iRhom2 with an extended, disordered N-terminal cytoplasmic tail. In light of its important regulatory properties, we propose to name this module the regulatory domain (R domain). Our previous work (Adrain et al., 2012) showed that iRhom2 is essential for the trafficking of TACE from the ER (**A**) to the *trans*-Golgi apparatus, where the proprotein convertase Furin cleaves off TACE's inhibitory prodomain, rendering TACE basally active (**B**). iRhom2 and TACE then traffic to the cell surface (**C**) where the interaction with iRhom2 ensures that TACE has only basal proteolytic activity because of reduced access to its substrates. Under stimulation conditions (e.g., PMA, GPCR activation, or TLR signaling) kinases phosphorylate iRhom2 within its R domain (**D**), which triggers the recruitment of 14-3-3 proteins to the iRhom2/TACE complex. 14-3-3 binding to the R domain of iRhom2 leads to dissociation of the transmembrane helices of the iRhom2/TACE complex (**E**). This enables increased TACE cleavage of its substrates, potentially mediated by increased exposure of TACE to its substrates upon detachment from iRhom2; dissociation may also induce conformational changes in TACE's extracellular domains that increases its capacity to cleave substrates.

Supplemental Experimental Procedures

Features within the iRhom2 N-terminus

Putative 14-3-3 binding sites were determined using the online 14-3-3-Pred tool (Madeira et al., 2015) (see http://www.compbio.dundee.ac.uk/1433pred/run/job=s2948603276). Putative phosphorylated residues on iRhom2 from large scale phosphoproteomics studies were retrieved using the online Phosphosite tool (see http://www.phosphosite.org/proteinAction.do?id=10848&showAll-Sites=true for a detailed overview). Analysis of disordered regions in the iRhom2 N-terminus were performed with the prediction algorithm PONDR (Obradovic et al., 2005) (http://www.pondr.com/).

Reagents

The following reagents were used: 1,10-phenanthroline (Sigma), Batimastat (BB94, Calbiochem), Marimastat (MM, Calbiochem), GI254023X (GI, Sigma), PD184352 (Sigma), Dynasore (Calbiochem), SB202190 (Calbiochem), SP600125 (Calbiochem), Lipopolysaccharide, E. coli 055:B5 (sc-221855A, Santa Cruz), poly(I:C) (11C21-MM, InvivoGen), Phorbol 12-myristate 13-acetate (PMA, Sigma), Ionomycin (IO, Cayman), Gastrin Releasing Peptide (GRP, mouse, Phoenix Pharmaceuticals), Cycloheximide (sc-3508, Santa Cruz), 1-Step™ PNPP Substrate (PIER37621, Termo Fisher), TACE fluorogenic peptide (ANASPEC, 72085), Alkaline Phosphatase (MBIEF0651, Fermentas), Phos-tag Acrylamide (AAL-107, Wako Chemicals), Dithiobis (succinimidyl proprionate) (DSP, 10731945, Pierce), Sulfo-NHS-LC-Biotin (Thermo Scientific, 21335), Neutravidin agarose resin (Thermo Scientific, 29200), mouse TNF ELISA (558534, BD Biosciences), Concanavalin A Agarose (786-216, G-biosciences), cell viability stain 7AAD (420403, Biolegend).

Antibodies

The following antibodies were used: TACE (Ab39162, Abcam), alpha-Tubulin (sc-8035, Santa Cruz), phospho-ERK (9102, Cell Signalling Technology), total-ERK (9106, Cell Signalling Technology), p97 (Thermo, MA1-21412), HA-HRP (clone 3F10, Roche), V5-HRP (R961-25, Life Technologies). 14-3-3 (pan-specific, 8312, Cell Signalling Technology), ADAM10 (19026, Millipore), Transferrin receptor (13-6800, Life Technologies). Anti-iRhom2 polyclonal antibodies specific to the mouse iRhom2 N-terminus (amino acids 1-373) or raised against the iRhom homology domain, were previously described (Adrain et al., 2012). For immuno-precipitations, magnetic beads were used: anti-HA (15222405, Pierce), MagnaBind Goat Anti-Mouse IgG Beads (2135, 4, Thermo Scientific), MagnaBind Goat Anti-Rabbit IgG Beads (21356, Thermo Scientific). The following antibodies were used specifically for flow cytometry: Fc-block (clone 2.4G2, BD Biosciences), anti-HA (Ab9110, Abcam), Alexa 633-labeled secondary antibody (A-21070, Invitrogen), phycoerythrin conjugated anti-TACE (FAB9301P, R&D Systems), or isotype control IgG1-Phycoerythrin conjugated (IC062P, R&D Systems).

Cell culture

HEK293 ET, RAW264.7 and iRhom1$^{-/-}$ 2$^{-/-}$ double KO (DKO) MEFs (previously described in Christova et al., 2013), untransformed and stably expressing iRhom2, were maintained under standard conditions.

BMDM were isolated from 12-16 weeks old WT and iRhom2$^{-/-}$ (iRhom2 KO) mice, and cultured as previously described (Adrain et al., 2012).

Retroviral transduction

HEK293 ET packaging cells (1 x 10^6) were transfected with pCL (-Eco, or 10A1) packaging plasmids (Naviaux et al., 1996) plus pM6P.BLAST empty vector (kind gift of F. Randow, Cambridge, UK) or pM6P containing mouse iRhom2 (WT or phospho-mutants) cDNA fused to a C-terminal HA tag. Cells transduced with the viral supernatant supplemented with polybrene 8 μg/mL, and selected with blasticidin (5 μg/mL) to generate stable cell lines. To transduce primary bone marrow-derived macrophages (BMDM) from iRhom2 KO mice, retroviruses were prepared as follows: HEK 293ET cells (24 x 10^6) were transfected with pMD-VSVG envelope plasmid, pMD-OGP helper plasmid (gift of Richard Mulligan), and empty vector pM6P.BLAST, or pM6P containing the iRhom2 (WT or non-phosphorylatable mutants) cDNA fused to a C-terminal HA tag. The viral supernatants were concentrated 300-fold using ultracentrifugation (90,000 g) at 4°C for 4h, followed by re-suspension in 0.1 % BSA in PBS. Cells were transduced with the concentrated virus, supplemented with 8 μg/ml polybrene. For the production of TACE and ADAM10 shRNA lentivirus, viral particles were concentrated 150-fold by ultracentrifugation of supernatants from HEK293 ET packaging cells transfected with pLVTHM-GFP plasmids encoding shRNAs specific to human ADAM10 or TACE (gift of Andreas Ludwig) plus pMD-VSVG and psPAX2.

Phos-tag gels and protein de-phosphorylation

All buffers used during sample preparation were devoid of EDTA (and 1,10-phenanthroline) to prevent chelation of the manganese ions required for phospho-protein binding to the Phos-tag acrylamide. Phosphate containing reagents were excluded to prevent competitive binding to the Phos-tag acrylamide. Cells were washed twice with ice-cold phos-tag wash buffer (10 mM Tris-HCl pH 7.4, 100 mM NaCl) before lysis in T-X100 lysis buffer supplemented with protease inhibitor cocktail, and phosphatase inhibitors (1.5 mM sodium orthovanadate, 10 mM sodium fluoride). Samples were run on 6 or 8 % acrylamide Tris-Glycine gels (prepared according to manufacturer instructions) supplemented with 5 μM Phos-tag acrylamide and 1 mM manganese chloride. Gels were run at 80 volts for approximately 5 hours (until the 75 KDa marker was at the bottom of the gel), and incubated in transfer buffer supplemented with 1 mM EDTA for 20 minutes to chelate manganese, followed by a second wash in transfer buffer. From this point onwards standard western blot protocols for transfer and antibody incubation were used. Where indicated, protein lysates (120 μg) were de-phosphorylated according to the manufacturer's instructions using 40 units of alkaline phosphatase and incubated at 37ºC for 1 hour prior to electrophoresis.

Flow Cytometry

After washing, the viability stain 7AAD was added and the samples were analyzed using a Cyan ADP analyzer (Beckman Coulter) and the FlowJo software, version 10.2. Cells were first gated based on size (SS vs FS) and then on singularity (Pulse Width vs FS). This was followed by 7AAD exclusion to gate for live cells (FL2 vs FL4). Live cells were gated on expression of HA or TACE (FL2 vs FL8). Unstained (Fc-blocked cells) were used as negative population. The staining index was calculated as: (fluorescence

intensity test population – fluorescence intensity negative population) / (2 x SD negative population) (Maecker et al., 2004).

TACE enzymatic assay on live cells

HEK293ET cells expressing empty vector, iRhom2-HA WT or iRhom2 Dead-HA were plated on 24 well plates ($1x10^6$ cells). The next day cells were washed in Optimem; for experiments involving PMA stimulation (Figure 7A,B) cells were starved for 6 hours in Optimem supplemented with 25 mM Hepes, the media was then changed to Optimem (25 mM Hepes) containing 0.75 μL of the fluorogenic TACE substrate and the drug treatments. Fluorescence was measured on a Victor 3 plate reader over 8 hours, at 37ºC, according to manufacturer instructions (ANASPEC SensoLyte 520 TACE Activity Assay Kit, 72085). TACE activity (Figure 5F, 7B), expressed as change in raw fluorescence units (RFU) per minute was calculated using linear regression fit of the raw fluorescence data. For all experimental conditions, TACE activity was background corrected for non-metalloprotease dependent cleavage of the peptide using BB94 treated samples. Time resolved fluorescence (AU, arbitrary units) shown in Figure 7A, is expressed as RFU for each experimental condition divided by the sum of the total RFU for each experiment; this step is a mathematical processing of raw data commonly used to minimize variability in absolute intensity values from experiment to experiment, and has been previously described for time-resolved luminescence from luciferase experiments (Bruning et al., 2012; Cavadas and Cheong, 2014).

Shedding assays

MEFs (3×10^5 in 6 well-plates) were transfected with 1 μg cDNA of TGF ✔-AP or BTC-AP using 3 μL Fugene. One day following transfection, cells were washed 3 times with serum free media before incubation for 1 hour in 1 mL Optimem (for basal shedding), followed by 1 hour with 1 mL Optimem containing 1 μM PMA or 2.5 μM ionomycin (for stimulated shedding), in the presence of metalloprotease inhibitors (BB94) and ADAM10 inhibitor (GI) where indicated. Supernatants from each incubation step were collected and incubated with the AP substrate p-nitrophenyl phosphate at room temperature. AP activity measured using a 96-well plate spectrophotometer (405 nm). Results are presented as PMA or IO "Stimulated shedding" calculated by dividing the PMA or IO-stimulated AP shedding by the constitutive shedding, as described previously (Sahin et al., 2006; Zheng et al., 2002).

Cloning of iRhom2-HA phospho-mutant constructs, R18-Dead mutant and TACE/ADAM10 domain swaps

The mouse iRhom2 Dead mutant fused to a C-terminal HA tag was inserted into empty vector pM6P.BLAST. This iRhom2 Dead DNA containing the 15 alanine mutated residues (Figure 4A) was prepared by gene synthesis (Gene Art, Life Technologies). To generate the 1-130, 131-212, 213-381, PCR was used to amplify the relevant fragments from the WT or iRhom2 Dead constructs, and Gibson cloning was used to assemble the final construct. Single serine to alanine phospho-mutants (Figure 4B,C,G,H) were generated by site-directed mutagenesis using KOD polymerase. To generate the R18-iRhom2 Dead mutant (Figure 5D-G), the R18 peptide sequence from the pSCM138 plasmid (Masters and Fu, 2001) was inserted into the N-terminus of the iRhom2 Dead mutant using Gibson cloning. TACE and ADAM10 V5 tagged

pcDNA6 constructs (Christova et al., 2013) were used to generate domain swap mutants of the ectodomain, transmembrane domain and cytoplasmic tails (Figure 7 D,E). DNA sequencing was used to confirm correct assembly of all constructs.

Co-immunoprecipitations

HEK293ET cells stably expressing empty vector, iRhom2-HA WT, Dead, phosphomutants, or the R18 mutant, were serum starved and stimulated with PMA (1 μM) for the indicated times (Figs. 5,7) before anti-HA immunoprecipitations. Alternatively, HEK293 ET cells were transfected with TACE/ADAM10 domain swap mutants and an empty vector or iRhom2-HA WT (Figure 7). Full details are provided in supplemental experimental procedures.Twenty-four hours after transfection cells were lysed for 10 minutes on ice in TX-100 lysis buffer (1% Triton X-100, 150 mM NaCl, 50 mM Tris-HCl, pH 7.4) containing complete protease inhibitor cocktail (Roche), 10 mM 1,10-phenanthroline (to inhibit TACE autoproteolysis) and phosphatase inhibitors 10 mM sodium fluoride and 1.5 mM sodium orthovanadate. Post-nuclear supernatants were pre-cleared with unconjugated magnetic beads for 90 minutes with rotation, followed by overnight incubation with anti-HA magnetic beads. Beads were washed for 10 minutes, 4 times, at 4ºC with Triton X-100 lysis buffer (with 1,10-phenanthroline and phosphatase inhibitors) supplemented with NaCl to 300 mM. Samples were eluted with 1.5 X sample buffer and incubated at 65ºC for 15 minutes before loading.

Mass spectrometry

Cells were washed twice with ice-cold PBS, and lysed for 10 minutes on ice in TX-100 lysis buffer containing complete protease inhibitor cocktail and phosphatase inhibitors. Post-nuclear supernatants were pre-cleared with unconjugated magnetic beads for 90 minutes with rotation, followed by 90 minutes incubation with anti-HA magnetic beads at 4ºC. Beads were washed for 10 minutes, 4 times, at 4ºC with 150 mM NaCl, 50 mM Tris-HCl, pH 7.4, supplemented with phosphatase inhibitors. For the data in Table 1, Table S1, Figure 4, Figure 5, Figure S1 and Figure S3, collected in Edinburgh, the following protocol was used. Immunoprecipitated samples were subjected to an on-beads digestion, peptide purification and mass spectrometry analysis as previously described (Turriziani et al., 2014). Briefly, the proteins bound to the beads were digested with mass spectrometry-grade porcine trypsin (Promega) overnight at room temperature. The proteolytic peptides were reduced and alkylated with DTT and Iodoacetamide, desalted and analysed on a Q-Exactive+ mass spectrometer coupled to a nano uHPLC (both Thermo Fisher Scientific). Analysis of phosphorylation sites were performed with MaxQuant software version 1.5.5.1, using human and mouse databases, Carbamylation as fixed and phospho (STY), N-terminal acetylation (protein) and oxidation (M) as variable modifications. The abundance of the different phosphopeptides was determined as the ratio obtained by dividing the intensity of the phosphopeptides by the intensity of the corresponding protein in each sample. N=2 biological replicates each. Data presented in Figure S3B, was collected from the mass-spec data from Edinburgh, and an additional set of experiments (2 biological replicates) performed in Vienna in iRhom2 immunoprecipitates, prepared as described above, with the addition of a cell permeable crosslinker (DSP).

For the data collected in Vienna (Figure S3B), a slightly different protocol was used, in brief, immunoprecipitates were enzymatically digested on 3 kD MWCO filters (Pall Austria Filter GmbH) using

an adaption of the FASP protocol as described previously (Bileck et al., 2014; Slany et al., 2016). After pre-concentration of the samples, protein reduction and alkylation was performed, then trypsin was added and incubated at 37°C for 18h. The digested peptide samples were dried and stored at -20°C until further LC-MS/MS analyses. Reconstitution of dried peptide samples was achieved by adding 5 µl 30% formic acid (FA) containing 10 fmol each of 4 synthetic standard peptides and further dilution with 40 µl mobile phase A (98% H_2O, 2% ACN, 0.1% FA), as described previously (Bileck et al., 2014; Wiśniewski et al., 2009). LC-MS/MS analyses were performed using a Dionex Ultimate 3000 nano LC-system coupled to a QExactive orbitrap mass spectrometer equipped with a nanospray ion source (Thermo Fisher Scientific). For LC-MS/MS analysis, 5µl of the peptide solution were loaded and pre-concentrated on a 2cm x75µm C18 Pepmap100 pre-column (Thermo Fisher Scientific) at a flow rate of 10 µl/min using mobile phase A. Following this pre-concentration, peptides were eluted from the pre-column to a 50cm x75µm Pepmap100 analytical column (Thermo Fisher Scientific) at a flow rate of 300nl/min and further separation was achieved using a gradient from 7% to 40% mobile phase B (80% ACN, 20% H_2O, 0.1% FA) over 85min including column washing and equilibration steps. For mass spectrometric analyses, MS scans were accomplished in the range from m/z 400-1400 at a resolution of 70000 (at m/z =200). Subsequently, data-dependent MS/MS scans of the 8 most abundant ions were performed using HCD fragmentation at 30% normalized collision energy and analyzed in the orbitrap at a resolution of 17500 (at m/z =200). Protein identification was achieved using Proteome Discoverer 1.4 (Thermo Fisher Scientific, Austria) running Mascot 2.5 (Matrix Science). Therefore, raw data were searched against the human proteome in the SwissProt Database (version 11/2015 with 20.193 entries) with a mass tolerance of 50 ppm at the MS1 level and 100 mmu at the MS2 level, allowing for up to two missed cleavages per peptide. Further search criteria included carbamidomethylation as fixed peptide modification and methionine oxidation as well as protein N-terminal acetylation as variable modifications.

Cell Surface Biotinylation

Biotinylation was performed as previously described for BMDM (Adrain et al., 2012) with small modifications, as detailed in supplemental experimental procedures. RAW264.7 macrophages (1.5x10^6 cells, 6 well plates) were moved to a cold room (at 4ºC), washed with ice-cold PBS pH 8.0 for 10 minutes, incubated with (1 mg/mL) Sulfo-NHS-LC-Biotin in PBS pH 8.0, according to the manufacturer's instructions. Dynasore (40 µM) was used during the biotinylation, to minimize internalization of the unreacted Biotin and biotinylated proteins. Following quenching with 50 mM Tris in PBS, cells were lysed for 10 minutes with TX-100 lysis buffer (1,10-phenathroline, protease inhibitors, 50 mM Tris), then biotin labelled surface proteins from post-nuclear supernatants were captured on neutravidin agarose resin at 4°C overnight. The resin was washed 3 times, 10 minutes, with TX-100 lysis buffer containing 450 mM NaCl at 4ºC. Samples were eluted with 1.5X sample buffer and incubated at 65ºC for 15 minutes, before loading.

Glycoprotein enrichment using Concanavalin A

To improve the detection of TACE, MEFs were lysed in TX-100 lysis buffer supplemented with 1 mM EDTA, 1 mM $MnCl_2$, 1 mM $CaCl_2$ and glycoproteins were captured using Concanavalin A (ConA) Agarose. Beads were washed twice in the same buffer and eluted by heating for 15 min at 65ºC in sample buffer

supplemented with 15% sucrose.

TNF ELISA in iRhom2 KO bone marrow-derived macrophages expressing iRhom2-HA

On day 4 post-isolation, iRhom2 KO BMDM were plated (1.2 x 10^5 cells, 12 well plate). The following day, cells were transduced with 10 µL of 300X concentrated retrovirus mixed with polybrene 8 µg/mL. Culture media were changed every second day and cells were stimulated at day 10 with 1µg/mL LPS for 3 hours. The supernatant was collected to measure TNF release using ELISA according to the manufacturer's instructions.

Table S1. iRhom2 (mouse) phosphorylation sites identified in large proteomic screens and in the current study

	S60	S83	S85	S87	S99	S147	Y199	T258	S293	S295	S298	S310	S357	S359	S360	T361
Proteomics screens	+	+	+	+	+	+	+	+	+	+	+	-	+	+	+	+
This study	+	+	-	-	-	+	-	-	-	-	+	+	-	+	-	-

Table S2. Phosphorylation-dependent changes in the iRhom2 interactome

iRhom2 interactors with altered binding upon PMA stimulation					
	# proteins	%			
WT PMA > WT	9	3,3			
WT PMA < WT	2	0,7			
WT PMA = WT	262	95,6			
**iRhom2 interactors with altered binding in the WT compared to the Dead mutant**					
DMSO			PMA		
	# proteins	%		# proteins	%
WT > EV	274	100	WT > EV	272	100
Dead < WT	15	5,5	Dead < WT	13	4,8
Dead > WT	4	1,5	Dead > WT	5	1,8
Dead = WT	255	93,1	Dead = WT	254	93,4

Supplementary References

Adrain, C., Zettl, M., Christova, Y., Taylor, N., and Freeman, M. (2012) Tumor Necrosis Factor Signaling Requires iRhom2 to Promote Trafficking and Activation of TACE. Science, *335*, 225–228.

Bileck, A., Kreutz, D., Muqaku, B., Slany, A., and Gerner, C. (2014) Comprehensive assessment of proteins regulated by dexamethasone reveals novel effects in primary human peripheral blood mononuclear cells. J Proteome Res, *13*, 5989–6000.

Bruning, U., Fitzpatrick, S.F., Frank, T., Birtwistle, M., Taylor, C.T., and Cheong, A. (2012) NFκB and HIF display synergistic behaviour during hypoxic inflammation. Cell. Mol. Life Sci., *69*, 1319–1329.

Cavadas, M.A.S., and Cheong, A. (2014) Monitoring of Transcriptional Dynamics of HIF and NFκB Activities. C.E. Badr, ed. (Totowa, NJ: Humana Press), pp. 97–105.

Christova, Y., Adrain, C., Bambrough, P., Ibrahim, A., and Freeman, M. (2013) Mammalian iRhoms have distinct physiological functions including an essential role in TACE regulation. EMBO Rep,

Madeira, F., Tinti, M., Murugesan, G., Berrett, E., Stafford, M., Toth, R., Cole, C., MacKintosh, C., and Barton, G.J. (2015) 14-3-3-Pred: improved methods to predict 14-3-3-binding phosphopeptides. Bioinformatics, *31*, 2276–2283.

Maecker, H.T., Frey, T., Nomura, L.E., and Trotter, J. (2004) Selecting fluorochrome conjugates for maximum sensitivity. Cytometry, *62A*, 169–173.

Masters, S.C., and Fu, H. (2001) 14-3-3 proteins mediate an essential anti-apoptotic signal. J Biol Chem, *276*, 45193–45200.

Naviaux, R.K., Costanzi, E., Haas, M., and Verma, I.M. (1996) The pCL vector system: rapid production of helper-free, high-titer, recombinant retroviruses. J Virol, *70*, 5701–5705.

Obradovic, Z., Peng, K., Vucetic, S., Radivojac, P., and Dunker, A.K. (2005) Exploiting heterogeneous sequence properties improves prediction of protein disorder. Proteins, *61 Suppl 7*, 176–182.

Sahin, U., Weskamp, G., Zheng, Y., Chesneau, V., Horiuchi, K., and Blobel, C.P. (2006) A sensitive method to monitor ectodomain shedding of ligands of the epidermal growth factor receptor. Methods Mol Biol, *327*, 99–113.

Slany, A., Bileck, A., Kreutz, D., Mayer, R.L., Muqaku, B., and Gerner, C. (2016) Contribution of Human Fibroblasts and Endothelial Cells to the Hallmarks of Inflammation as Determined by Proteome Profiling. Mol Cell Proteomics, *15*, 1982–1997.

Wiśniewski, J.R., Zougman, A., Nagaraj, N., and Mann, M. (2009) Universal sample preparation method for proteome analysis. Nat Methods, *6*, 359–362.

Zheng, Y., Schlondorff, J., and Blobel, C.P. (2002) Evidence for regulation of the tumor necrosis factor alpha-convertase (TACE) by protein-tyrosine phosphatase PTPH1. J Biol Chem, *277*, 42463–42470.
